# Quantitative disease risk scores from EHR with applications to clinical risk stratification and genetic studies

**DOI:** 10.1038/s41746-021-00488-3

**Published:** 2021-07-23

**Authors:** Danqing Xu, Chen Wang, Atlas Khan, Ning Shang, Zihuai He, Adam Gordon, Iftikhar J. Kullo, Shawn Murphy, Yizhao Ni, Wei-Qi Wei, Ali Gharavi, Krzysztof Kiryluk, Chunhua Weng, Iuliana Ionita-Laza

**Affiliations:** 1 Department of Biostatistics, Columbia University, New York, NY USA; 2 Division of Nephrology, Department of Medicine, Columbia University, New York, NY USA; 3 Department of Neurology and Neurological Sciences, Stanford University, Stanford, CA USA; 4 Quantitative Sciences Unit, Department of Medicine, Stanford University, Stanford, CA USA; 5 Department of Pharmacology and Center for Genetic Medicine, Northwestern University, Chicago, IL USA; 6 Department of Cardiovascular Medicine, Mayo Clinic, Rochester, MN USA; 7 Department of Biomedical Informatics, Harvard Medical School, Boston, MA USA; 8 Research Information Science and Computing, Mass General Brigham, Boston, MA USA; 9 Division of Biomedical Informatics, Cincinnati Children’s Hospital Medical Center, Cincinnati, OH USA; 10 Department of Biomedical Informatics, Vanderbilt University, Nashville, TN USA; 11 Department of Biomedical Informatics, Columbia University, New York, NY USA

**Keywords:** Translational research, Statistics

## Abstract

Labeling clinical data from electronic health records (EHR) in health systems requires extensive knowledge of human expert, and painstaking review by clinicians. Furthermore, existing phenotyping algorithms are not uniformly applied across large datasets and can suffer from inconsistencies in case definitions across different algorithms. We describe here quantitative disease risk scores based on almost unsupervised methods that require minimal input from clinicians, can be applied to large datasets, and alleviate some of the main weaknesses of existing phenotyping algorithms. We show applications to phenotypic data on approximately 100,000 individuals in eMERGE, and focus on several complex diseases, including Chronic Kidney Disease, Coronary Artery Disease, Type 2 Diabetes, Heart Failure, and a few others. We demonstrate that relative to existing approaches, the proposed methods have higher prediction accuracy, can better identify phenotypic features relevant to the disease under consideration, can perform better at clinical risk stratification, and can identify undiagnosed cases based on phenotypic features available in the EHR. Using genetic data from the eMERGE-seq panel that includes sequencing data for 109 genes on 21,363 individuals from multiple ethnicities, we also show how the new quantitative disease risk scores help improve the power of genetic association studies relative to the standard use of disease phenotypes. The results demonstrate the effectiveness of quantitative disease risk scores derived from rich phenotypic EHR databases to provide a more meaningful characterization of clinical risk for diseases of interest beyond the prevalent binary (case-control) classification.

## Introduction

The increasing availability of rich phenotype data from electronic health records (EHR), such as the multicenter Electronic Medical Records and Genomics (eMERGE) network^1,2^, BioVU^3^ from Vanderbilt University, the Geisinger Health System’s DiscovEHR in Pennsylvania^4^, the Harvard University/Partners Healthcare system i2b2 effort^5^, the United Kingdom Biobank (UKBB)^6^, and their linking to biobanks of human germline DNA samples provides great opportunities for genomic-based research^7–9^. However, inferring phenotypes from International Classification of Diseases (ICD) codes is not trivial, and many algorithms have already been proposed^10^. Although these algorithms can generate high-quality case/control labels for specific diseases, a main limitation is that they require extensive knowledge and involvement of human experts, are time-consuming, are not systematically applied, and can lead to inconsistencies of case definition for different algorithms^11^. Furthermore, they tend to perpetuate the view of common diseases as discrete entities rather than residing on a continuum. Indeed, there is a spectrum of any specific complex disease and whether an individual is labeled as a case can be arbitrary. We consider here an alternative view, namely that common diseases are the extreme tails of quantitative traits, and all of us are susceptible to specific diseases to a greater or lesser extent. Thinking quantitatively about common diseases could prove beneficial for genomic studies of phenotypes derived from EHR^12,13^.

Therefore an alternative approach to expert derived phenotype labels is to derive phenotypic risk scores that quantify the propensity of an individual to develop a disease. Recently, a method to compute phenotypic risk scores (PheRS) has been proposed in the context of rare Mendelian phenotypes^14^. This method is conceptually simple, and the authors have showed that it can be effective in identifying individuals with undiagnosed Mendelian phenotypes, and can pinpoint potentially relevant pathogenic mutations. However, the main disadvantage is that it requires a careful selection of phenotypic features for good performance, which limits its scalability and appeal, especially in applications to more complex diseases. Inspired by this approach, we propose to investigate almost unsupervised methods for phenotype risk prediction for more common diseases, that need minimal input from clinicians and can incorporate not only binary phenotypic features but also quantitative measurements such as laboratory values. In this paper, we have two main objectives: (1) derive quantitative disease risk scores for a given disease, and (2) integrate these newly derived continuous phenotypes with targeted sequencing data in eMERGE-seq to perform genetic association tests. As with the PheRS for Mendelian phenotypes, we demonstrate that the proposed quantitative disease risk scores are effective in pinpointing undiagnosed cases based on the phenotypic features in the EHR, and more generally in clinical risk stratification, and help improve the power of genetic association studies. Although we focus our applications on six complex traits in this paper, our methods are general and can be employed for clinical risk stratification and genetic association studies for any common disease of interest with minimal involvement of clinical expertize.

The score we propose can be viewed as a generalization of the simple PheRS score. Specifically, we develop a quantitative disease risk score as a weighted linear combination of multiple phenotypic features, these include phecodes, but can also include laboratory values, other clinical covariates, as well as natural language processing (NLP) features extracted from clinical notes when available. Our proposed approach is based on a linear combination of multiple principal components (LPC) of the phenotypic feature matrix. More details are given in the “Methods” section. We compare its performance with that of the previously proposed Phenotype Risk Score (PheRS), which combines binary phecodes in an individual^14^, with phecodes’ weights based on the inverse prevalence of the phecode in a given population. We also include comparisons with PheNorm, a recent phenotyping algorithm that does not require expert-labeled individuals for training^15^.

## Results

### Description of datasets and phenotypes

We focus here on the data from the eMERGE Network, containing 102,597 subjects, each with records in terms of ICD-9/ICD-10 codes, which the World Health Organization established to map health conditions to designated codes. In this paper we make use of phecodes, hierarchical groupings of ICD-9/ICD-10 codes, originally developed for phenome-wide association studies (PheWAS)^16^. The current version of phecodes (Version 1.2) has 1,866 phecodes, and 102,597 subjects in the eMERGE network have phecodes available. In addition to rich phenotypic data, 21,363 individuals have sequencing data in 109 genes chosen as part of the eMERGE specific sequencing platform^17^.

We describe here the datasets we used for training and testing purposes. Although we do not use a case-control label in the training, our approaches need to be trained on datasets enriched in cases for the disease under consideration, although as we explain later, only an approximate definition of case is required. That is why we refer to the proposed methods as almost unsupervised.

The Chronic Kidney Disease (CKD) case-control cohort was constructed from 98,486 subjects with available kidney function data excluding 4,111 patients with end-stage renal disease (ESRD). We considered 1,817 non-zero prevalence phecodes, which are divided into 18 categories (Supplementary Table 1). We took advantage of a CKD phenotyping algorithm recently developed within eMERGE to diagnose and place individuals on a CKD staging grid of albuminuria by estimated glomerular filtration rate (eGFR)^18^. Using this algorithm, the individuals were classified as having no CKD (controls) or having CKD of various severity, classified as G1, G2, G3a, G3b, and G4 stage. We divided the case-control dataset in Table 1 into two parts, and used 50% of the cases and 50% of controls for training purposes. We first use the entire set of phecodes to build the quantitative disease risk scores, without any pre-selection based on the disease under consideration. We compare performance with the scenario when phecodes are pre-selected based on their potential relevance to the disease of interest. Note that the selection of phecodes does not require expert knowledge, and highly automated approaches can be used for this purpose. We discuss the pre-selection of phecodes in more detail in the next section.

**Table 1:** Number of individuals (cases vs. controls) in training and test datasets.

Disease	#phecodes	Training		Testing	
		Control	Case	Control	Case
CKD	104	7,334	17,897	7,334	17,898
CAD	93	5,479	5,479	21,916	5,479
T2D	132	3,767	3,767	9,960	3,768
HF	90	1,724	1,724	10,806	1,724
Dementia	143	879	879	7,570	879
GERD	162	3,233	3,233	5,918	3,233
CKD cases	G1	G2	G3a	G3b	G4
	2,370	22,204	6,612	3,332	1,277

The number of cases at different CKD stages are also reported. The #phecodes is the number of pre-selected phecodes used for deriving the quantitative disease risk scores for validation purposes (excludes the case defining phecodes).

For consistency, we focused on the same set of 98,486 subjects as above in the analyses of additional phenotypes, including Coronary Artery Disease (CAD), Type 2 Diabetes (T2D), Heart Failure (HF), Dementia, and Gastroesophageal Reflux Disease (GERD). CAD case definition was based on a composite of myocardial infarction^19^. Myocardial infarction was based on self-report or hospital admission diagnosis. This included individuals with ICD-9 codes of 410.X, 411.0, 412.X, or 429.79, or ICD-10 codes of I21.X, I22.X, I23.X, I24.1, or I25.2 in hospitalization records. The case/control definitions of T2D, HF, Dementia, and GERD are based on the validated algorithms available at the Phenotype KnowledgeBase (PheKB)^20–23^. We used 50% of the cases and an equal number of controls for training, and the rest of the cases and controls as test set for performance evaluation. The number of cases and controls for each phenotype are listed in Table 1. Those individuals who are neither case nor control for a given phenotype are treated as having unknown status.

#### Pre-selection of phecodes

Although our quantitative disease risk scores can be based on all available phecodes, it is of interest to compare their performance to the situation when only a pre-selected set of phecodes that we deem possibly relevant to the disease under consideration are included in the computation of the scores. The relevance of a phecode can be determined based on clinical expert knowledge, but this is not necessary and highly automated approaches such as associations between phecodes and polygenic risk scores (PRS) for diseases of interest can be employed. Only for the purposes of validation and assessing predictive performance, we have excluded the case defining phecodes in Supplementary Table 2 from the computations (this is valid also for the scenario when all phecodes are being used as above), except for PheNorm which uses the case defining phecodes in the training (see “Methods” section for details). For CKD, we included 104 CKD phecodes (manually selected by experts), 93 CAD phecodes (among the ‘circulatory system’ category, those with *p*-values < 10^−5^ in logistic regression of each phecode against CAD PRS), 132 T2D phecodes (manually selected by experts among the significant phecodes with *p*-values < 10^−5^ in logistic regression of each phecode against T2D PRS), 90 HF phecodes (CAD feature phecodes with HF case defining phecodes removed), 143 Dementia phecodes (from the ‘mental disorder’ and ‘neurological’ categories) and 162 GERD phecodes (from the ‘digestive’ category). More details on the PRS calculation for the individuals in eMERGE are in the “Methods” section.

The selection of ‘relevant’ phecodes as described above is scalable to many common diseases given the general availability of genetic (e.g., GWAS) data for many such phenotypes. These existing GWAS datasets can serve as the discovery datasets for building the PRS. Then, PRS can be computed for individuals in large biobanks such as the UK biobank^24^, and the ‘relevant’ phecodes for a disease of interest can be determined based on the association between such PRS and individual phecodes. Therefore, pre-selection of phecodes using associations with PRS is possible without the need to have GWAS data for the individuals in the EHR under consideration. Nonetheless, we also provide the option to compute the quantitative disease risk scores based on all phecodes.

### LPC has improved prediction accuracy and robustness relative to PheRS and PheNorm

We trained the different approaches, including PheRS, PheNorm, and LPC, on the training datasets as explained before using the same phenotypic features for the three methods (with the difference that PheNorm uses the case defining phecodes in the training, whereas LPC and PheRS do not), and then computed the quantitative disease risk scores for the individuals in the test datasets. The weights (prevalences) for the PheRS for each phenotype were estimated based on the controls in the training datasets. For the proposed LPC approach with all phecodes, the Tracy-Widom test suggested 169, 148, 131, 112, 99, and 145 significant eigenvalues (PCs) of the covariance matrix of feature phecodes for CKD, CAD, T2D, HF, Dementia, and GERD, respectively. The number of PCs reduces to 9, 12, 14, 11, 8, and 17, with respective pre-selected phecodes for each studied phenotype. Note that the case-control labels for the individuals in the training datasets were only used in order to select the signs for the PCs in the linear combination approach, LPC (since the signs of the PCs are arbitrary). As we explain later, the choice of training set based on the gold/silver standard labels is not necessary, and weakly defined (soft) labels (e.g., based on presence of eGFR slope measurement for CKD, or a phecode of the target phenotype) are sufficient for training.

**Fig. 1:**
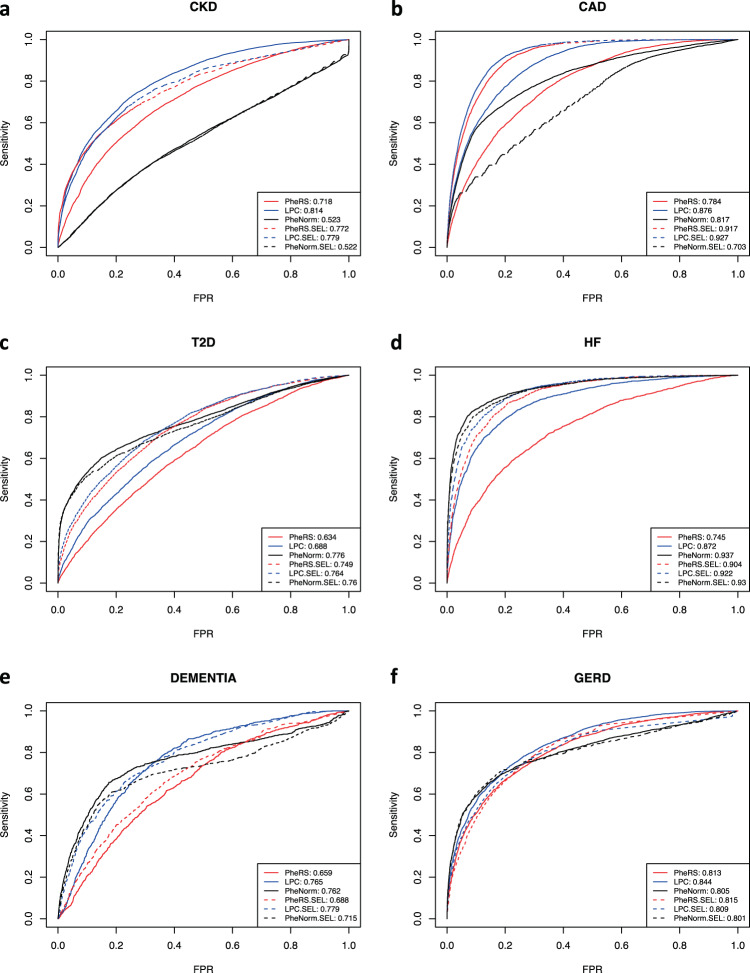
ROC curves cases vs. controls for six phenotypes. ROC curves of six quantitative disease risk scores along with their AUROCs for **a** CKD (cases including G1, G2, G3a/b, and G4 stages), **b** CAD, **c** T2D, **d** HF, **e** Dementia, and **f** GERD. Quantitative disease risk scores are derived based on all phecodes (PheRS, LPC, and PheNorm), or pre-selected feature phecodes (PheRS.SEL, LPC.SEL, and PheNorm.SEL).

We compared PheRS, PheNorm, and LPC scores using either all phecodes or the pre-selected phecodes in terms of area under the receiver operating characteristic curve (AUROC) and the area under the precision-recall curve (AUPRC) in the test datasets. Note that the labels used in the performance evaluation are derived using algorithms available in PheKB rather than gold-standard labels from chart review. We acknowledge this limitation to the results we present due to the current limited availability of such gold-standard labels for the diseases we considered. We observed that LPC with all phecodes has the largest AUROC for CKD, CAD, Dementia and GERD, and the second largest for T2D and HF compared with PheRS and PheNorm: 0.813 vs. 0.718 vs. 0.523 for CKD, 0.876 vs. 0.784 vs. 0.817 for CAD, 0.688 vs. 0.634 vs. 0.776 for T2D, 0.872 vs. 0.745 vs. 0.937 for HF, 0.765 vs. 0.659 vs. 0.762 for Dementia, and 0.844 vs. 0.813 vs. 0.805 for GERD (Fig. 1). Although PheNorm seems to perform better for T2D and HF, it is important to note that PheNorm uses the case defining phecodes in the training stage whereas they are not included in the LPC and PheRS calculations for these prediction accuracy assessments. LPC also has the largest AUPRC for CKD, CAD, and GERD (Supplementary Fig. 1). LPC with pre-selected phecodes exhibits the largest AUROC for all phenotypes except for HF when compared with PheRS and PheNorm: 0.779 vs. 0.772 vs. 0.522 for CKD, 0.927 vs. 0.917 vs. 0.702 for CAD, 0.764 vs. 0.749 vs. 0.760 for T2D, 0.922 vs. 0.904 vs. 0.930 for HF, 0.779 vs. 0.688 vs. 0.715 for Dementia, and 0.809 vs. 0.815 vs. 0.801 for GERD (Fig. 1). Similar results were obtained when comparing controls with cases at different CKD G stages (Supplementary Figs. 2 and 3). Note that the AUROC values are much higher when contrasting controls with more advanced stages of CKD (G3b: 0.903 and G4: 0.908 for LPC with all other phecodes). Overall, the proposed LPC score is fairly robust to the inclusion of noisy phecodes, while PheRS can have a substantial loss in accuracy when including all phecodes. For PheNorm, pre-selecting phecodes does not in general result in performance improvement.

We observe that PheNorm has poor performance for CKD relative to other phenotypes. PheNorm score derivation relies on the count of case defining phecodes (see details in “Methods” section), so its performance depends on the correlation between the counts of case defining phecodes and PheKB derived labels. We notice high correlations between the count of case defining phecodes and case/control labels for CAD, T2D, HF, Dementia, and GERD (Spearman *ρ* = 0.823, 0.826, 0.873, 0.545, and 0.899, respectively), while the correlation for CKD is only 0.272. This is probably due to the CKD algorithm being primarily based on lab test results. The denoising step of PheNorm regresses the normalized count of case defining phecodes on a randomly corrupted version of the features including the response variable itself and all other predictive features, with the intention to utilize the underlying association among all the features to recover the lost information of the response variable due to the random corruption. The pre-selected or all other phecodes that are included as additional features might only provide limited additional information of the presence of CKD in this denoising step, hence PheNorm may need to leverage information from features other than phecodes to better predict the CKD case/control status.

### LPC improves clinical risk stratification relative to PheRS and PheNorm

**Fig. 2:**
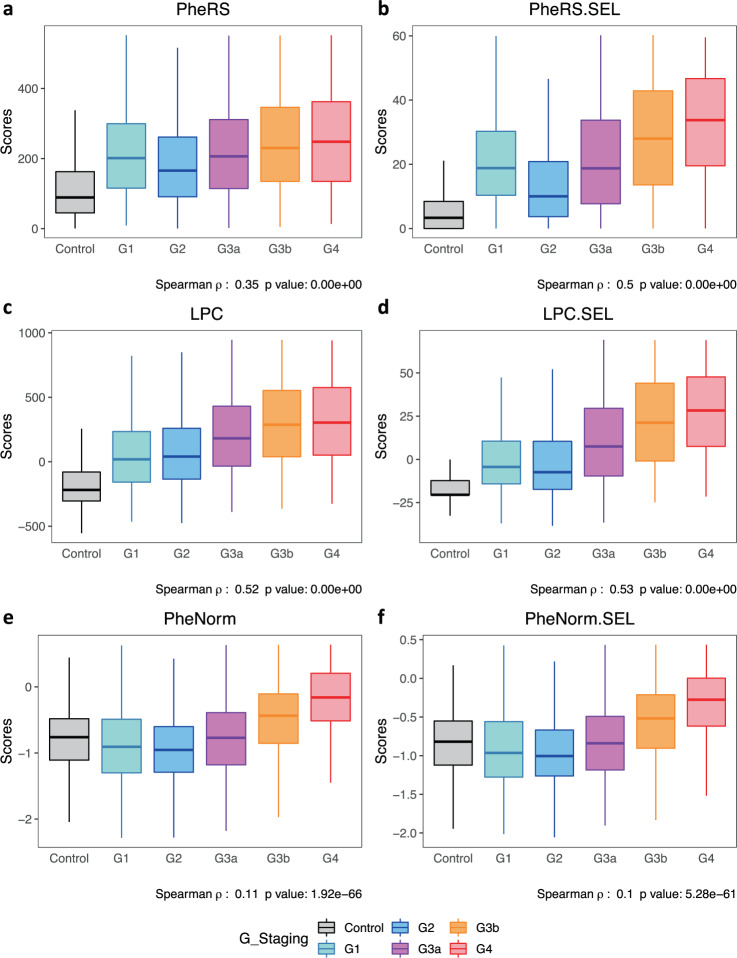
Quantitative disease risk scores vs. CKD G-staging. Boxplots of quantitative disease risk scores **a** PheRS, **b** PheRS.SEL, **c** LPC, **d** LPC.SEL, **e** PheNorm, and **f** PheNorm.SEL. Quantitative disease risk scores are derived based on all phecodes (PheRS, LPC, PheNorm), or 110 pre-selected CKD feature phecodes (PheRS.SEL, LPC.SEL, and PheNorm.SEL). The center line, lower and upper bounds of the box represent the median, first quartile (*Q*_1_, or 25th percentile), and third quartile (*Q*_3_, or 75th percentile) of the data, respectively. The whisker is drawn up (down) to the largest (smallest) observed point from the data that falls within 1.5 times the interquartile range (= *Q*_3_ − *Q*_1_) above (below) the *Q*_3_ (*Q*_1_).

The LPC risk score correlates very well with the CKD staging (Fig. 2), providing support to the use of LPC as a measure of disease severity (note that the distributions of risk scores for CKD Control and G1 stages are similar since G1 is defined as individuals who have normal renal function but have other abnormality that makes them classified as CKD). LPC shows the strongest correlation with the CKD staging compared with PheRS and PheNorm; specifically, LPC with all phecodes has the largest Spearman’s correlation coefficient *ρ* compared with PheRS and PheNorm, *ρ* = 0.52 (*p* ~ 0) vs. *ρ* = 0.35 (*p* ~ 0) vs. 0.11(*p* = 1.92E−66), respectively. When restricting to pre-selected phecodes, the correlation for PheRS becomes comparable to that for LPC (PheRS *ρ* = 0.5 (*p* ~ 0) vs. LPC *ρ* = 0.53 (*p* ~ 0)).

**Fig. 3:**
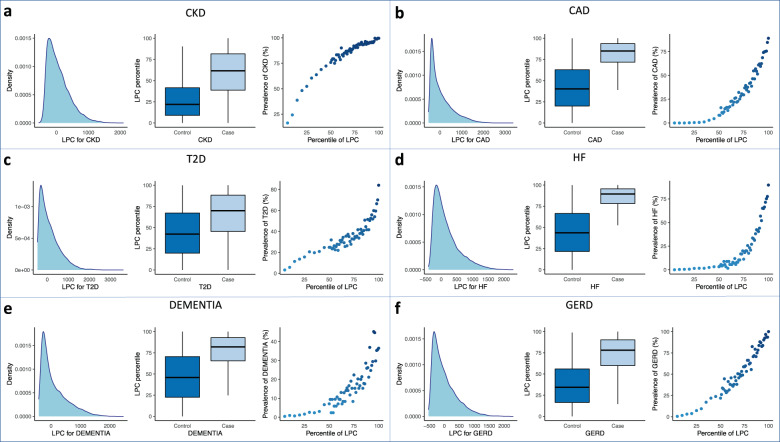
Distribution of final LPC scores for six phenotypes in the test set. LPC risk scores are derived based on **all** phecodes. Estimated density and distribution of LPC risk scores cases vs. controls for **a** CKD (cases including G1, G2, G3a/b, and G4 stages), **b** CAD, **c** T2D, **d** HF, **e** Dementia, and **f** GERD. For each phenotype: left, distribution of LPC risk scores in the test set. Middle, LPC risk score percentiles among cases vs. controls. Right, case prevalence in 60 bins according to the percentiles of LPC risk scores. The center line, lower and upper bounds of the box represent the median, first quartile (*Q*_1_, or 25th percentile), and third quartile (*Q*_3_, or 75th percentile) of the data, respectively. The whisker is drawn up (down) to the largest (smallest) observed point from the data that falls within 1.5 times the interquartile range (= *Q*_3_ − *Q*_1_) above (below) the *Q*_3_ (*Q*_1_).

Furthermore, we investigated the prevalence of cases in the test set among individuals at different percentiles of LPC risk score. The proportion of cases increases among the individuals with higher LPC scores, as expected (Fig. 3 and Supplementary Fig. 4). We note that the prevalence of Dementia cases among individuals with higher LPC score for Dementia is lower (29.8% ~ 44.7%) than for other diseases (CKD: 98.8% ~ 99.6%, CAD: 74.8% ~ 89.1%, T2D: 54.0% ~ 84.0%, HF: 66.4% ~ 89.7%, GERD: 92.4% ~ 100%), which may suggest that Dementia is a more difficult disease to diagnose than other diseases. The prevalence of cases among individuals with high LPC scores based on pre-selected phecodes has an overall similar or higher range compared to LPC (CKD: 97.6% ~ 100%, CAD: 80.0% ~ 96.4%, T2D: 80.3% ~ 98.6%, HF: 84.8% ~ 93.7%, Dementia: 52.4% ~ 71.8%, GERD: 87.0% ~ 97.8%). The PheRS and PheNorm show worse performance, with lower prevalences of cases among individuals with high PheRS/PheNorm scores relative to LPC, except for T2D and Dementia with high PheNorm scores (CKD: 92.1% ~ 97.6%/71.5% ~ 97.6%, CAD: 51.8% ~ 72.2%/55.8% ~ 86.9%, T2D: 46.0% ~ 60.1%/94.2% ~ 99.3%, HF: 40.8% ~ 59.5%/85.6% ~ 99.2%, Dementia: 10.7% ~ 24.7%/34.1% ~ 61.2%, GERD: 81.5% ~ 97.8%/91.3% ~ 97.8%). Restricting phenotypic features to pre-selected phecodes results in substantially higher prevalence of cases for high PheRS (CKD: 98.6% ~ 100%, CAD: 83.6% ~ 94.5%, T2D: 68.6% ~ 98.5%, HF: 73.8% ~ 91.3%, Dementia: 29.4% ~ 36.5%, GERD: 83.7% ~ 95.7%), but small improvement for high PheNorm (CKD: 74.6% ~ 76.7%, CAD: 58.3% ~ 89.4%, T2D: 90.5% ~ 99.3%, HF: 79.2% ~ 100%, Dementia: 36.5% ~ 55.2%, GERD: 92.4% ~ 96.7%) (Supplementary Figs. 5–8).

**Fig. 4:**
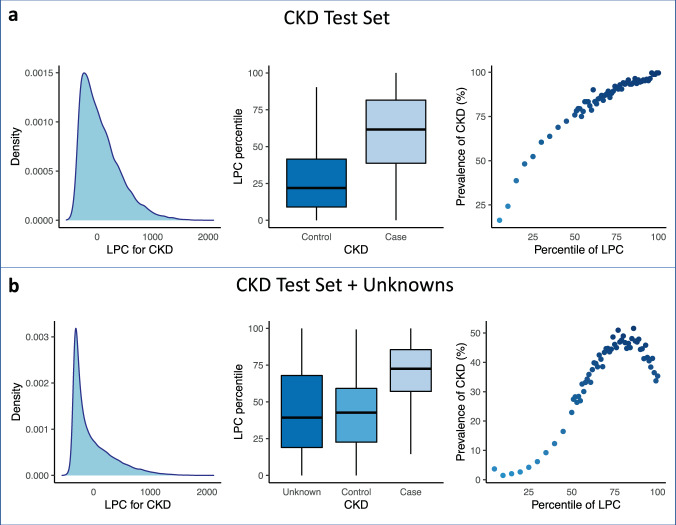
Distribution of CKD LPC risk scores in the test set vs. test set + individuals with unknown status. Estimated density and distribution of LPC risk scores cases vs. controls in the **a** CKD test set and **b** with unknown status individuals added. LPC risk scores are derived based on **all** phecodes. Left, distribution of LPC risk scores. Middle, LPC risk score percentiles among cases vs. controls. Right, the prevalence of phenotype in 60 bins according to the percentiles of LPC risk scores. The center line, lower and upper bounds of the box represent the median, first quartile (*Q*_1_, or 25th percentile), and third quartile (*Q*_3_, or 75th percentile) of the data, respectively. The whisker is drawn up (down) to the largest (smallest) observed point from the data that falls within 1.5 times the interquartile range (= *Q*_3_ − *Q*_1_) above (below) the *Q*_3_ (*Q*_1_).

We have repeated the analyses above by considering a less stringent control definition, namely including in addition to controls defined by the algorithm also those with unknown status. For CKD, we have noticed an interesting pattern. For the most extreme values of the LPC quantitative disease risk scores, we noticed a sudden decrease in prevalence, suggesting that there are individuals with high quantitative disease risk scores that have unknown case status (Fig. 4). Similar results are obtained when using the other scores (PheRS, and PheNorm), with all or selected phecodes (Supplementary Figs. 9–13). This emphasizes the difficulties in obtaining accurate phenotypic labels, and the potential of the quantitative disease risk scores as discussed here to identify undiagnosed cases.

### LPC improves weighting of the disease-relevant phecodes relative to PheRS and PheNorm

**Fig. 5:**
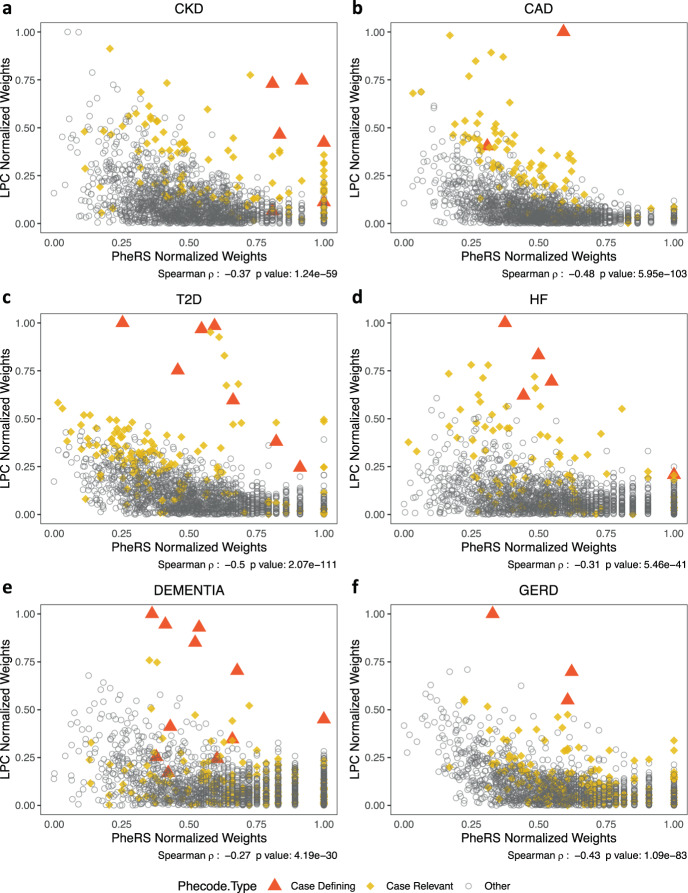
Weights for all phecodes used to build PheRS and LPC for six phenotypes. Scatter plots of weights for **a** CKD (cases including G1, G2, G3a/b, and G4 stages), **b** CAD, **c** T2D, **d** HF, **e** Dementia, and **f** GERD. The weights for the case defining and pre-selected phecodes are highlighted.

We show here that the weights for the disease ‘relevant’ phecodes are significantly higher compared with those of the rest of the phecodes (the ‘irrelevant’ ones) for the proposed LPC score (Table 2). The weights here are derived based on the corresponding training dataset for each disease, including all phecodes. We also show that case defining phecodes and pre-selected phecodes tend to have higher weights in LPC relative to PheRS and PheNorm (Fig. 5 and Supplementary Fig. 14). Although the PheRS weights are also significantly higher for the relevant phecodes, the *p*-values from the Wilcoxon rank-sum test are much larger than those for the LPC score. PheNorm does not perform well in selecting the relevant phecodes, likely due to the approximate L2 penalty that dropout training implies^25^. We observe that LPC has the smallest rank-sum of pre-selected phecodes and the largest percentage of relevant phecodes among top-ranked phecodes for all phenotypes but Dementia, and the smallest rank-sum of case defining phecodes for all phenotypes except CKD (Supplementary Fig. 15). Note that the case defining phecodes have no individual weights in PheNorm, since the case defining phecodes are used together in constructing the response variable (i.e., the number of case defining phecodes) in the training component of PheNorm (see the “Methods” section for more details).

**Table 2:** Weights for ‘relevant’ phecodes vs. the rest of the phecodes.

Disease	PheRS	LPC	PheNorm
CKD	9.82E−05	1.42E−25	1.36E−05
CAD	2.41E−03	2.49E−33	7.36E−01
T2D	8.10E−08	3.54E−37	7.79E−01
HF	1.41E−02	4.66E−17	6.29E−01
Dementia	9.38E−01	1.04E−02	4.02E−02
GERD	4.15E−03	1.02E−04	1.54E−01

The ‘relevant’ phecodes include the case defining and pre-selected phecodes. Wilcoxon rank-sum test one-sided *p*-values comparing weights for ‘relevant’ phecodes vs. the rest of the phecodes are reported (with alternative hypothesis: ‘relevant’ phecodes have greater weights).

### Applications to genetic association studies using the eMERGE-seq dataset

The eMERGE Network created an eMERGE specific sequencing platform and sequenced a cohort of 25,000 participants (including 14,813 Non-Hispanic White (NHW), 3,110 African American, 1,437 Asian, and 1,301 Hispanic) with an eMERGE-seq panel that includes 109 actionable genes^17^. The 109 genes include 56 genes from the American College of Medical Genetics and Genomics (ACMG) published a recommendation for actionable findings and additional genes deemed as potentially actionable that were selected across all eMERGE sites^26^. Low-quality variant calls were filtered out based on GATK recommendations, which resulted in 57,398 variants. Among the 25,000 participants, 21,363 individuals have both sequencing data and quantitative disease risk scores. More details on quality control steps are given in the “Methods” section.

We have performed comprehensive association tests with both rare and common variants within each individual gene^27,28^. For each gene, we have combined several tests, as follows:

1. Burden and dispersion tests for common and low-frequency variants (MAF > 0.01) with Beta (MAF, 1, 25) weights, where Beta ( ⋅ ) is the probability density function of the beta distribution with shape parameters 1 and 25.
2. Burden and dispersion tests for rare variants (MAF < 0.01 and minor allele count (MAC) ≥ 5) with Beta (MAF,1, 25) weights.
3. Burden and dispersion tests for rare variants, weighted by functional annotations (CADD, PolyPhen).
4. Burden test for aggregation of ultra-rare variants with MAC < 5 (e.g., singletons, doubletons).
5. Single variant score tests for common, low-frequency, and rare variants in the gene.

We then applied the aggregated Cauchy association test^29^ to combine the *p*-values from 1 to 5 to compute the final *p*-value for a gene. We adjusted for age, gender, and ten principal components of genetic variation. The distribution of LPC scores is right-skewed (Fig. 3), therefore we assumed a generalized linear model (GLM) based on the inverse-Gaussian distribution.

We focused on those rare variants that are predicted to be deleterious, as follows. First, we identified rare variants that have allele frequency < 0.01 in the Genome Aggregation Database (gnomAD v2.1.1), which integrates a large population reference cohort spanning 125,748 exome sequences^30^. Next, variant functional consequences were annotated using ANNOVAR^31^ and RefSeq transcript data^32^. Moreover, the deleteriousness of protein-coding variants was assessed using PolyPhen-2^33^. The final set of selected qualifying variants includes missense variants predicted as probably damaging by PolyPhen-2 and loss-of-function (LoF) variants with putative functional effects such as frameshift insertion, frameshift deletion, splicing variant, start-loss, stop-gain, and stop-loss.

We report those genes with *p*-value < 0.05/18 000 = 2.78E−06 as exome-wide significant. We focus here on the results of the LPC score using pre-selected phecodes since these scores led to more powerful genetic analyses; the genetic analyses of the quantitative disease risk scores including all phecodes is less powerful likely due to the increased environmental variation that requires increased sample sizes for improved power. Indeed no significant results are found for the LPC (Supplementary Figs. 16–19), PheRS (Supplementary Figs. 20–23), and PheNorm scores with all phecodes (Supplementary Figs. 24–27).

**Fig. 6:**
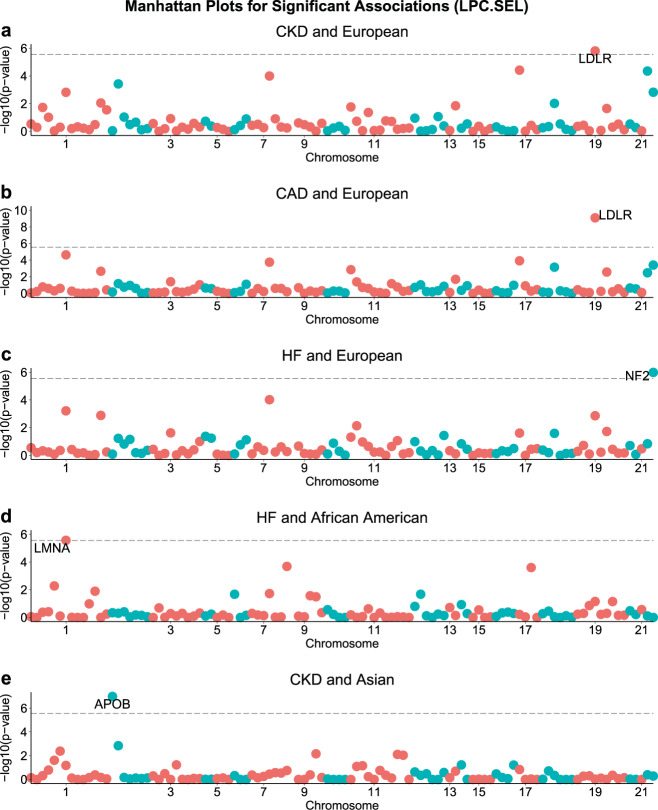
Exome-wide significant gene-based test results for 107 autosomal genes on the eMERGE-seq panel using LPC with pre-selected phecodes for six diseases. Results are shown for those phenotypes and ethnic groups with at least one exome-wide significant result: **a** CKD and European, **b** CAD and European, **c** HF and European, **d** HF and African American, **e** CKD and Asian. The horizontal line corresponds to the exome-wide significance level.

The significant results for LPC with pre-selected phecodes are shown in Fig. 6, and the results for the individual ethnic groups are shown in Supplementary Figs. 28–31. The associations we detected are driven by rare variants with MAF < 0.01. Indeed, for almost all significant associations the overall signal is driven by predicted deleterious variants with MAF < 0.01 and MAC ≥ 5 (Table 3 and Supplementary Table 6). The exception is *LMNA* and CAD, with the association driven by ultra-rare variants with MAC < 5. We show for each significant gene the individual variants that contribute to the overall signal along with their MAC (Supplementary Figs. 33–37). The results for the two other scores PheRS and PheNorm with pre-selected phecodes are shown in Table 3, Supplementary Table 6, and Supplementary Figs. 38, 39–48, and 49–52. There are no significant results for PheNorm. In contrast, several genes are exome-wide significant when using PheRS with pre-selected phecodes (Supplementary Fig. 38).

**Table 3:** Genes showing significant association with at least one disease using LPC and PheRS with pre-selected phecodes.

Ethnicity	Disease	Gene	p.all	p.common	p.rare
LPC with pre-selected phecodes					
European	CKD	LDLR	1.530E−06	NA	1.530E−06
European	CAD	LDLR	7.852E−10	NA	7.852E−10
European	HF	NF2	1.009E−06	NA	1.009E−06
African American	HF	LMNA	2.680E−06	NA	2.680E−06
Asian	CKD	APOB	1.006E−07	6.146E−01	7.317E−08
PheRS with pre-selected phecodes					
European	CKD	CHEK2	4.128E−07	NA	4.128E−07
European	CAD	LDLR	5.816E−07	NA	5.816E−07
European	CAD	CFTR	1.266E−06	NA	1.266E−06
European	HF	CFTR	1.189E−06	NA	1.189E−06
European	HF	LDLR	2.306E−06	NA	2.306E−06
African American	GERD	CACNA1S	1.907E−06	2.455E−01	1.387E−06

The *p*-values from the combined test (p.all), the tests including only common variants (p.common), and tests including only rare variants (p.rare). No exome-wide significant results were identified for PheNorm. Only the significant results are shown for combined tests. See Supplementary Table 6 for both combined and individual tests results.

We detected significant associations between the LPC score with pre-selected phecodes for CKD and rare variants in *LDLR* (*p* = 1.53E−06) in the European cohort. Recent large-scale multi-ethnic GWAS studies have demonstrated that *LDLR*, low-density lipoprotein receptor, is significantly associated with CAD^34,35^, LDL^36,37^, and total cholesterol^36,37^. Similarly, associations with rare variants in *LDLR* were also identified in whole-exome sequencing studies for LDL-C levels and risk of myocardial infarction (MI)^38–40^. Furthermore, mutations that affect *APOE* binding to LDL receptors (LDLR) render renal cells to be more susceptible to glomerular injury based on animal studies^41^. In fact, we also detected significant associations with rare variants in *LDLR* (*p* = 7.85E−10) and the LPC score for CAD, also within the European cohort. Rare variants in *LMNA* also show significant association with Heart Failure in the African American cohort (*p* = 2.68E−06). *LMNA* encodes the lamin proteins that compose the nuclear membrane. Mutations in *LMNA* can disrupt the reorganization of lamin-associated chromatin domains in cardiac myocytes through altered DNA methylation and dysregulated gene expression^42^. Recently, several studies have observed an association between *LMNA* mutations and high risk of various cardiac disorders, which include dilated cardiomyopathy that reduces the heart’s ability to supply blood and causes heart failure^42–44^. Finally, we have also detected a significant association between rare variants in *NF2* and Heart Failure (*p* = 1.0E−06). *NF2* encodes the neurofibromin 2 (merlin), which regulates Hippo signaling pathways in cardiomyocytes and potentially aggravates ischemia/reperfusion injury in the heart^45^.

Within the Asian group, we detected significant associations of rare variants in *APOB* and LPC score for CKD (*p* = 1.0E−07). *APOB* encodes the apolipoprotein B that is a major protein component of LDL. Higher serum levels of apolipoprotein A1 were associated with lower prevalence of CKD and higher eGFR in two multi-ethnic populations, while a higher apolipoprotein B/A1 ratio was significantly associated with lower eGFR^46^.

For the PheRS score with pre-selected phecodes, we found significant associations for CKD and rare variants in the checkpoint 2 gene (*CHEK2*, *p* = 4.13E−07). Variants in *CHEK2* have been found to be significantly associated with eGFR in the context of hypertension^47^. As with LPC, we have detected a significant association between rare variants in *LDLR* and CAD PheRS score with pre-selected phecodes (*p* = 5.82E−07). PheRS for Heart Failure also shows a significant association with rare variants in *LDLR* (*p* = 2.31E−06). Additionally, CAD and Heart Failure PheRS are significantly associated with rare variants in *CFTR* (*p* = 1.27E−06, *p* = 1.19E−06). *CFTR* was previously identified as a susceptibility locus for CAD in a European-ancestry GWAS study^48^. Within the African American cohort, we also detected a significant association between GERD PheRS and rare variants in *CACNA1S* (*p* = 1.38E−06).

#### Replication of significant association findings in other ethnic groups

We investigated if the significant associations discovered in a specific ancestry could be replicated in other ancestries. We report the *p*-values of the combined tests for each of the four populations for the five significant loci detected by LPC with pre-selected phecodes and their associated phenotypes in Supplcase defining phecodesementary Table 4. The significant association of *LDLR* and CKD in the European ancestry is nominally significant (*p* < 0.05) in the African American cohort (*p* = 1.43E−03). The association between *LMNA* and HF discovered in the African American ancestry is replicated in the European population (*p* = 5.99E−04). Among the six significant associations found by PheRS with pre-selected phecodes, three significant associations in the European ancestry, *CFTR* and CAD, *CFTR* and HF, and *LDLR* and HF, are replicated in the African American population with nominal significance (*p* = 3.53E−02, *p* = 1.95E−02, and *p* = 4,81E−02, respectively). These replications provide additional evidence for our genetic findings, and also reflect the increased power of the European and African American cohorts which have larger sample sizes relative to the two other ancestries.

#### Binary phenotypes defined based on phecodes

A common way to analyze phenotypes derived from EHR data is to use binary ICD codes for phenotypes of interest^49^. To compare with such a strategy, here we used the case defining phecodes (Supplementary Table 2) to identify cases and controls in each ethnic group, and then ran the same association tests as discussed above for the quantitative disease risk scores, focusing on data from Europeans since the European group is the only group with an adequate number of cases (Supplementary Table 5). Results are shown in Supplementary Fig. 53. We have not detected any significant associations between rare variants in 107 autosomal genes and the binary phenotypes, in contrast to several associations we have identified with the quantitative disease risk scores, as described above. This comparison with results from binary phecode-defined phenotypes illustrates the potential benefit for genetic association studies when using quantitative disease risk scores.

## Discussion

We have proposed an almost unsupervised method to derive a quantitative disease risk score, LPC, based on phenotypic features available in EHR from health systems. The proposed quantitative disease risk score has several advantages: (1) it can be derived on a large number of individuals using only minimal clinical input, (2) it can be derived with only weak labels, as opposed to limited gold/silver standard label information that may be available in the EHR, (3) it can help stratify individuals according to disease risk severity, and identify undiagnosed cases, (4) it can identify disease-relevant features, and (5) it can take advantage of biobanks linked to clinical information from EHR to perform potentially more powerful genetic association studies. Beyond these advantages, LPC, as a quantitative disease risk score, represents a different, promising direction in deriving phenotypes from EHR features, that can provide a more meaningful characterization of clinical risk for diseases of interest beyond the prevalent binary (case-control) classification.

Although we have focused here on using structured data such as phecodes, it is important to supplement these with other unstructured data including NLP features extracted from clinical notes, health information generated via mobile health devices, etc. to improve prediction accuracy. The proposed score can easily incorporate such features when available. In our limited analyses, incorporating lab measurements for CKD did not significantly improve the prediction accuracy. A possible reason may be that the presence of lab measurements is correlated to the presence of relevant phecodes/symptoms and that decreases the potential benefit from including them (Supplementary Fig. 54).

The different quantitative disease risk scores can be regarded as weighted linear combinations of phenotypic features, with different ways to derive the weights. The weighting scheme is particularly important when all phecodes are included, and as we have shown the proposed LPC score can upweight the disease-relevant phecodes, leading to higher accuracy relative to the comparison scores, PheRS and PheNorm. However, when the phecodes are pre-selected according to their potential relevance for the disease under investigation, the weighting strategy is less important, as expected.

The proposed score is easy to compute using features available in the EHR, is scalable to many diseases, and is almost unsupervised. This is important because labeling clinical data requires detailed knowledge from human experts, and can be time-consuming. Although LPC does not require explicit labels in the training stage, it does require some knowledge of case status, so training sets can be enriched in cases. An alternative to highly accurate labels are labels derived from simple filters, such as at least one relevant ICD-9 code for the phenotype of interest (or similar silver standard labels). We explored the performance when using such different training sets, and found minimal effect on the performance. Another important advantage of the proposed quantitative score is that it can serve as a measure of disease severity, and therefore helps identify individuals with higher severity, and even individuals without a clinical diagnosis. In particular, for CKD we have shown that LPC correlates very well with the CKD staging by estimated glomerular filtration rate (eGFR) and even helps identify individuals with very high LPC scores which are missed by the phenotyping algorithm because they miss laboratory-based eGFR measurement.

We have performed genetic associations with the derived quantitative disease risk scores, using targeted sequencing data from eMERGE-seq, and have shown that we can identify several significant associations with variants in potentially relevant genes for the diseases considered here. In contrast, using phecodes to define binary phenotypes as commonly done in genetic association studies based on EHR phenotypes has led to no significant genes in Europeans, the group with the largest number of cases for the phenotypes considered here, highlighting the increase in power afforded by quantitative disease risk scores. The availability of large EHR systems linked to biobanks such as the eMERGE and UK Biobank opens up the possibility to perform genetic associations with a large number of phenotypes genome-wide. One of the issues recently recognized in such studies is the potential for highly imbalanced datasets where the number of controls can be much higher than the number of cases for a particular phenotype^49^; conventional association tests, which do not adjust for case-control imbalance can lead to biased results and increases in false positives. The proposed approach by deriving quantitative disease risk scores for all individuals alleviates this bias.

In summary, we propose almost unsupervised methods to derive quantitative disease risk scores from information in EHR that require minimal input from domain experts, and that can improve the utility of using such EHR-derived phenotypes in genomics research. Future work in developing new quantitative disease risk scores could be beneficial for leveraging the rich phenotypic information in EHR.

## Methods

### Notations

Suppose we have *J* phenotypic features for a given set of *n* subjects; these include phecodes, but can also include laboratory values and other clinical covariates. Each phenotypic feature is centered and scaled by its sample standard deviation in the preprocessing step. The goal is, for a given (complex) disease phenotype, to construct a quantitative disease risk score (QRS) as a weighted linear combination of multiple phenotypic features, i.e., the score for subject *i* is defined as

1 $${\text{QRS}}_{i}=\mathop{\sum }\limits_{j=1}^{J}{w}_{j}{x}_{ij},$$

where *x*_*ij*_ is the standardized value of *j*th phenotypic feature for subject *i*. Here we consider several possible (almost) unsupervised methods to derive the set of weights *w*_*j*_’s. We denote by ***X***_*n*×*J*_ the matrix of *J* standardized phenotypic features for the *n* subjects. Let ***Q*** be the *J* × *J* sample correlation matrix of the *J* phenotypic features.

### Phenotype risk score (PheRS)

The phenotype risk score (PheRS) has been recently proposed to combine binary phecodes in an individual^14^, with phecodes’ weights based on the inverse prevalence of the phecode in the controls in the training dataset. Given a dataset of *N* subjects, the weight for phenotypic feature *j* is calculated as:

2 $${w}_{j}={\mathrm{log}}\,\frac{N}{{n}_{j}},$$

where *n*_*j*_ is the number of individuals with phenotypic feature *j*. Hence, less prevalent phenotypic features are given higher weight compared to the more common ones. The rationale behind this weighting scheme is that lower frequency phecodes are more likely to be related to the risk of disease in general, an assumption that may be reasonable. However, these weights are not related to the phenotype under consideration.

For subject *i*, the PheRS for a specific disease is calculated as in^14^:

3 $${\text{PheRS}}_{i}=\mathop{\sum }\limits_{j=1}^{J}{w}_{j}{{\mathbb{1}}}_{\{\text{subject}i\text{has phenotypic feature}j\}}.$$

### PheNorm

PheNorm is a phenotyping algorithm that does not require expert-labeled samples for training^15^. PheNorm relies on automated feature curation. To make it comparable to LPC and PheRS, we only trained PheNorm using phecode features. We implemented a version of PheNorm in two steps, as follows.

In the first step, we normalize the most predictive features such as the number of ICD codes or mentions of the target phenotype to resemble a normal mixture distribution. Specifically, we denote by *x*_PHECODE_ the number of case defining phecodes for a given disease. We use the health care utilization measure denoted by *x*_utl_ to normalize *x*_PHECODE_ since *x*_PHECODE_ tends to be high for subjects with more health care utilization regardless of their true disease status; the number of distinct age at observation in ICD code history is considered in our implementation. The distribution of the normalized count of phecodes ${z}_{\text{PHECODE}}={\mathrm{log}}\,(1+{x}_{\text{PHECODE}})-\alpha {\mathrm{log}}\,(1+{x}_{\text{utl}})$ with an appropriate choice of *α* is approximately a normal mixture distribution with *z*_PHECODE_∣*Y* ~ *N*(*μ*_*Y*_, *σ*^2^), where *Y* is an indicator of the true disease status. The optimal value of *α* is chosen to minimize the difference between the empirical distribution of *z* and its normal mixture approximation (see ref. ^15^ for more details). In the second step, we aggregate the information in the larger set of additional features (pre-selected or all other phecodes, excluding the case defining phecodes) with denoising self-regression via dropout training. The data matrix with columns of the normalized counts of case defining phecodes and set of candidate features *Z* = [*z*_PHECODE_, *z*_1_,…,*z*_*p*_], is randomly corrupted to obtain $\tilde{Z}$ with

4 $${\tilde{Z}}_{ij}={Z}_{ij}^{{W}_{ij}}{(\text{Mean}({Z}_{.j}))}^{1-{W}_{ij}},$$

where Mean (*Z*_.*j*_) is the mean of the *j*th column *Z* and {*W*_*ij*_} are independent and identically distributed Bernoulli random variables with drop out rate ${\mathbb{P}}({W}_{ij}=0)=r$ (*r* = 0.3 in our applications). Then *z*_PHECODE_ is predicted with $\tilde{Z}$ by ordinary least squares regression to obtain the regression coefficient vector ***β***. The final PheNorm score of subject *i* can be obtained by the weighted linear combination of health care utilization and candidate feature sets, with feature vector *z*_*i*_,

5 $${\text{PheNorm}}_{i}={z}_{i}^{\top }{\boldsymbol{\beta }}.$$

The actual implementation follows the scripts in R package sureLDA^50^, where random sampling with replacement of observations in each training set is used to form a bootstrap of size 10^5^.

### Principal component based score (LPC)

Principal component analysis (PCA) is a standard approach to reduce the dimension of the phenotypic feature space, and identify a small number of principal components (PCs). In particular, we have the spectral decomposition of ***Q***, ${\boldsymbol{Q}}=\mathop{\sum }\nolimits_{j = 1}^{J}{\lambda }_{j}{{\boldsymbol{u}}}_{j}{{\boldsymbol{u}}}_{j}^{\top }$, where *λ*_1_ ≥⋯≥ *λ*_*J*_ > 0 are eigenvalues of ***Q*** and ***u***_*j*_ is the *j*th eigenvector associated with the *j*th largest eigenvalue.

One possible composite score is based on using the entries of the top eigenvector ***u***_1_ (i.e., the loadings) as the weights for the *J* phenotypic features. Beyond the first principal component, it is possible that additional principal components are also informative^51,52^, and so we consider combining multiple PCs as a linear combination (LPC), especially when including a large number of phenotypic features. The general form of the LPC score for subject *i* is

6 $${\text{LPC}}_{i}=\mathop{\sum }\limits_{k=1}^{K}{\beta }_{k}{\text{PC}}_{ik},$$

where *K* ≤ *J* represents the number of PCs being included in the linear combination, and **PC** = ***XU*** is the PC score matrix (***U*** is the matrix of eigenvectors). *K* can be determined using the Tracy-Widom test^53^, a hypothesis test to identify significant eigenvalues of the covariance matrix.

There are several considerations with the LPC method. The first is the choice of the sign of individual PCs in the linear combination, since the signs of the PCs are arbitrary. We use the training data to help us identify the sign of each PC. Namely, the sign of each PC is adjusted so that the mean PC value of "cases” is higher than that for "controls”. We refer to the LPC approach as almost unsupervised, as we do use some approximate label information to determine the signs of individual PCs in the linear combination. However, we do not need to use gold or even silver standard labels, and weakly defined labels are sufficient (e.g., we could define as "cases” those individuals with some lab result present, e.g., the estimated glomerular filtration rate (eGFR) in the case of CKD). The second important consideration is the choice of weights. We choose here to use the corresponding eigenvalues *λ* as weights, so higher weight is assigned to those components with higher amount of variance explained. However the lower PCs (corresponding to smaller eigenvalues) can be as useful for prediction as the top PCs, and hence the approach here may not be optimal. If some amount of labeled data is available, weights can be learned by regression models, such as principal component regression and partial least squares.

Another possible approach would be to do non-negative matrix factorization (NMF)^54^. Unlike PCA, NMF constrains the factor loadings to be non-negative. Although we do not expect improved accuracy compared with the less constrained PCA approach, NMF can lead to better interpretability.

### Construction of feature matrix

Each individual in the eMERGE network has raw longitudinal records of ICD-9 and ICD-10 codes, which can be mapped to phecodes^55^, and the presence of a particular phecode is defined by at least two occurrences of the corresponding ICD-9 or ICD-10 codes in individual health records. Each phecode is used as a proxy of the corresponding condition. Note that here the absence of a phecode may be due to no assessment of the condition or no record of healthy condition. The feature matrix of 98,486 individuals is centered and scaled before splitting into training and test sets for the Eigen and PC-based approaches. The phecode features are on the original scale for PheRS derivation.

### Polygenic risk score calculation

We used the LDPred computational algorithm^56^ to derive a genome-wide polygenic risk score for CKD, CAD, and type 2 diabetes in the eMERGE cohort (*n* = 102, 138). We used the optimized GWAS summary statistics (weights) for these traits from^19,57,58^. More specifically, we calculated the PRS by summing the genotype of each risk allele carried by an individual and weighting each variant by its natural logarithm of the relative risk extracted from the GWAS. We then performed an association between these PRS scores and each phecode in turn.

### Quality control of eMERGE-seq panel variants

Quality control (QC) for the sequencing dataset is performed based on the quality metrics of variant and genotype calling according to GATK best practices recommendations^59^. Specifically, we filter out the low-quality SNVs with QD (quality by depth) < 2, MQ (root mean square mapping quality) < 40, FS (Fisher strand) > 60, SOR (strand odds ratio) > 3, MQRankSum (mapping quality rank-sum test) < −12.5, or ReadPosRankSum (read position rank-sum test) < −8. For indels, we exclude the variants that have QD < 2, ReadPosRankSum < −20, FS > 200, or SOR > 10. After QC, the resulting dataset includes 57,398 variants.

### Ethics statement

The study was approved by the Columbia University Institutional Review Board (IRB protocol numbers IRB-AAAP7926 and IRB-AAAO4154) and individual IRBs at all eMERGE-III network sites contributing human genetic and clinical data. All eMERGE participants provided informed consent to participate in genetic studies.

### Reporting summary

Further information on research design is available in the Nature Research Reporting Summary linked to this article.

## Supplementary information

Supplementary Information

Reporting Summary

## Data Availability

The development version of R package QDRS and documentation of quantitative disease risk scores from eMERGE dataset are available online (https://github.com/danqingxu/QDRS). The eMERGE-III genetic datasets with linked phenotypes are accessible through dbGAP (accession number: phs001584.v1.p1).
