# Supplementary material for: Quantitative disease risk scores from EHR with applications to clinical risk stratification and genetic studies
Source: NPJ Digit Med. 2021 Jul 23;4:116. doi: 10.1038/s41746-021-00488-3 (PMC8302667; doi:10.1038/s41746-021-00488-3)
Supplement: Supplementary file 1 — Supplementary Information [file 41746_2021_488_MOESM1_ESM.pdf]

Supplementary Materials

Quantitative disease risk scores from EHR with applications to clinical risk stratification and genetic studies

Danqing Xu[1], Chen Wang[1,2,*], Atlas Khan[2,*], Ning Shang[2], Zihuai He[3,4], Adam Gordon[5], Iftikhar J. Kullo[6], Shawn Murphy[7,8], Yizhao Ni[9], Wei-Qi Wei[10], Ali Gharavi[2], Krzysztof Kiryluk[2], Chunhua Weng[11,†], Iuliana Ionita-Laza[1,†]

[1] Department of Biostatistics, Columbia University, New York, NY, USA

[2] Division of Nephrology, Department of Medicine, Columbia University, New York, NY, USA

[3] Department of Neurology and Neurological Sciences, Stanford University, Stanford, CA 94305, USA

[4] Quantitative Sciences Unit, Department of Medicine, Stanford University, Stanford, CA, 94305, USA

[5] Department of Pharmacology and Center for Genetic Medicine, Northwestern University, Chicago, IL, USA

[6] Department of Cardiovascular Medicine, Mayo Clinic, Rochester, MN, USA

[7] Department of Biomedical Informatics, Harvard Medical School, Boston, MA, USA

[8] Research Information Science and Computing, Mass General Brigham, Boston, MA, USA

[9] Division of Biomedical Informatics, Cincinnati Children's Hospital Medical Center, Cincinnati, OH, USA

[10] Department of Biomedical Informatics, Vanderbilt University, Nashville, TN , USA

[11] Department of Biomedical Informatics, Columbia University, New York, NY, USA

[*] contributed equally to this work

[†] contributed equally to this work

Supplementary Figures

Boxplots are drawn to display variation and distribution of the quantitative disease risk scores. For the boxplots appear hereafter, the elements are defined as the following: the center line, lower and upper bounds of box represent the median, first quartile (Q_1, or 25th percentile), and third quartile (Q_3, or 75th percentile) of the data, respectively. The whisker is drawn up (down) to the largest (smallest) observed point from the data that falls within 1.5 times the interquartile range ($= Q_3 - Q_1$) above (below) the Q_3 (Q_1).

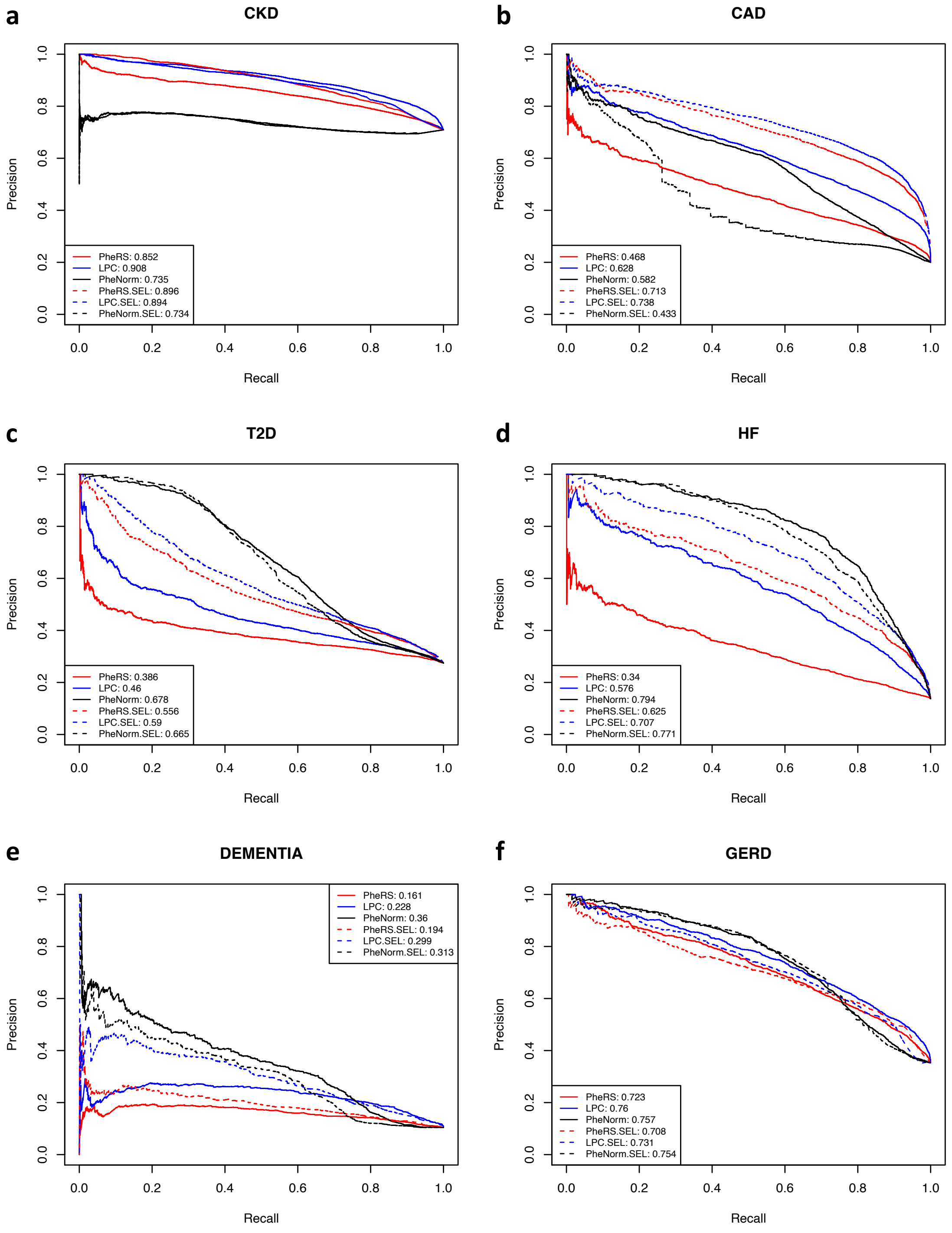

Supplementary Figure 1: **PR curves cases vs. controls for six phenotypes.** PR curves of six quantitative disease risk scores along with their AUPRCs for **a** CKD (cases including G1, G2, G3a/b, and G4 stages), **b** CAD, **c** T2D, **d** HF, **e** Dementia, and **f** GERD. Quantitative disease risk scores are derived based on all phecodes (PheRS, LPC, PheNorm), or pre-selected feature phecodes (PheRS.SEL, LPC.SEL, PheNorm.SEL).

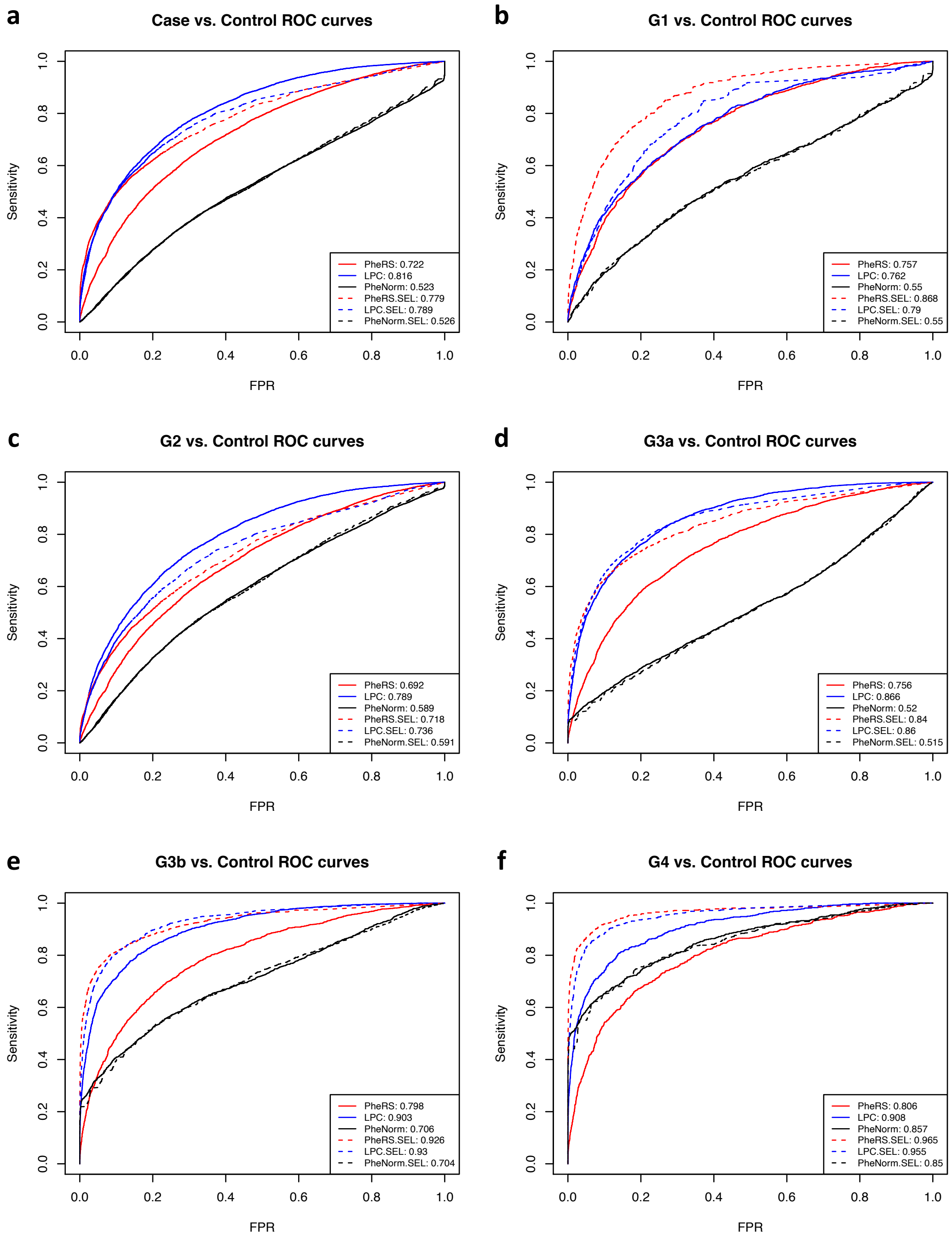

Supplementary Figure 2: **ROC curves for CKD cases vs. controls, and CKD cases at different G stages vs. controls.** ROC curves of six quantitative disease risk scores along with their AUROCs for **a** CKD cases (cases G1, G2, G3a/b, and G4 stages), **b** CKD G1, **c** CKD G2, **d** CKD G3a, **e** CKD G3b, and **f** CKD G4b. Quantitative disease risk scores are derived based on all phecodes (PheRS, LPC, PheNorm), or pre-selected feature phecodes (PheRS.SEL, LPC.SEL, PheNorm.SEL).

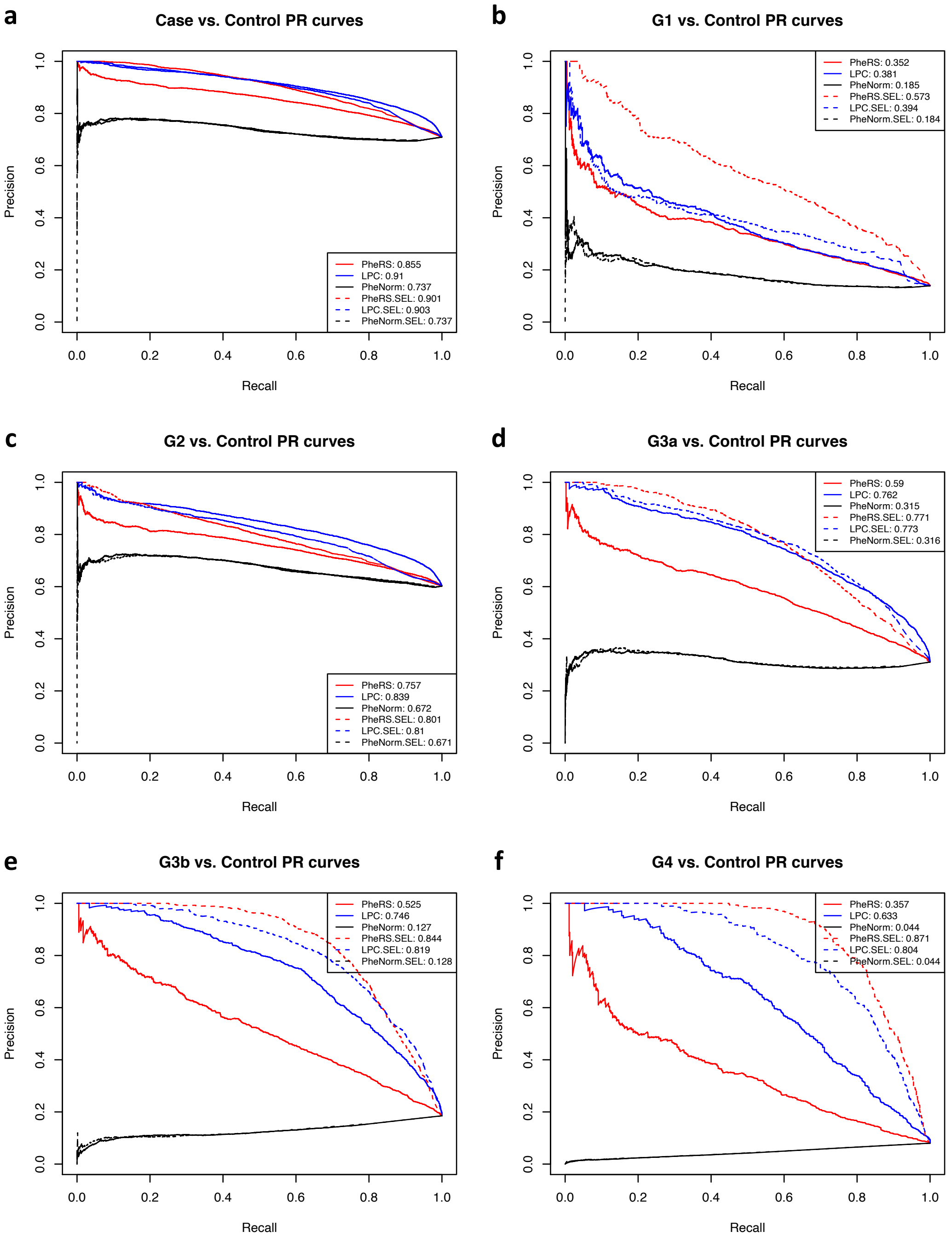

Supplementary Figure 3: **PR curves for CKD cases vs. controls, and CKD cases at different G stages vs. controls.** PR curves of six quantitative disease risk scores along with their AUPRCs for **a** CKD cases (cases G1, G2, G3a/b, and G4 stages), **b** CKD G1, **c** CKD G2, **d** CKD G3a, **e** CKD G3b, and **f** CKD G4b. Quantitative disease risk scores are derived based on all phecodes (PheRS, LPC, PheNorm), or pre-selected feature phecodes (PheRS.SEL, LPC.SEL, PheNorm.SEL).

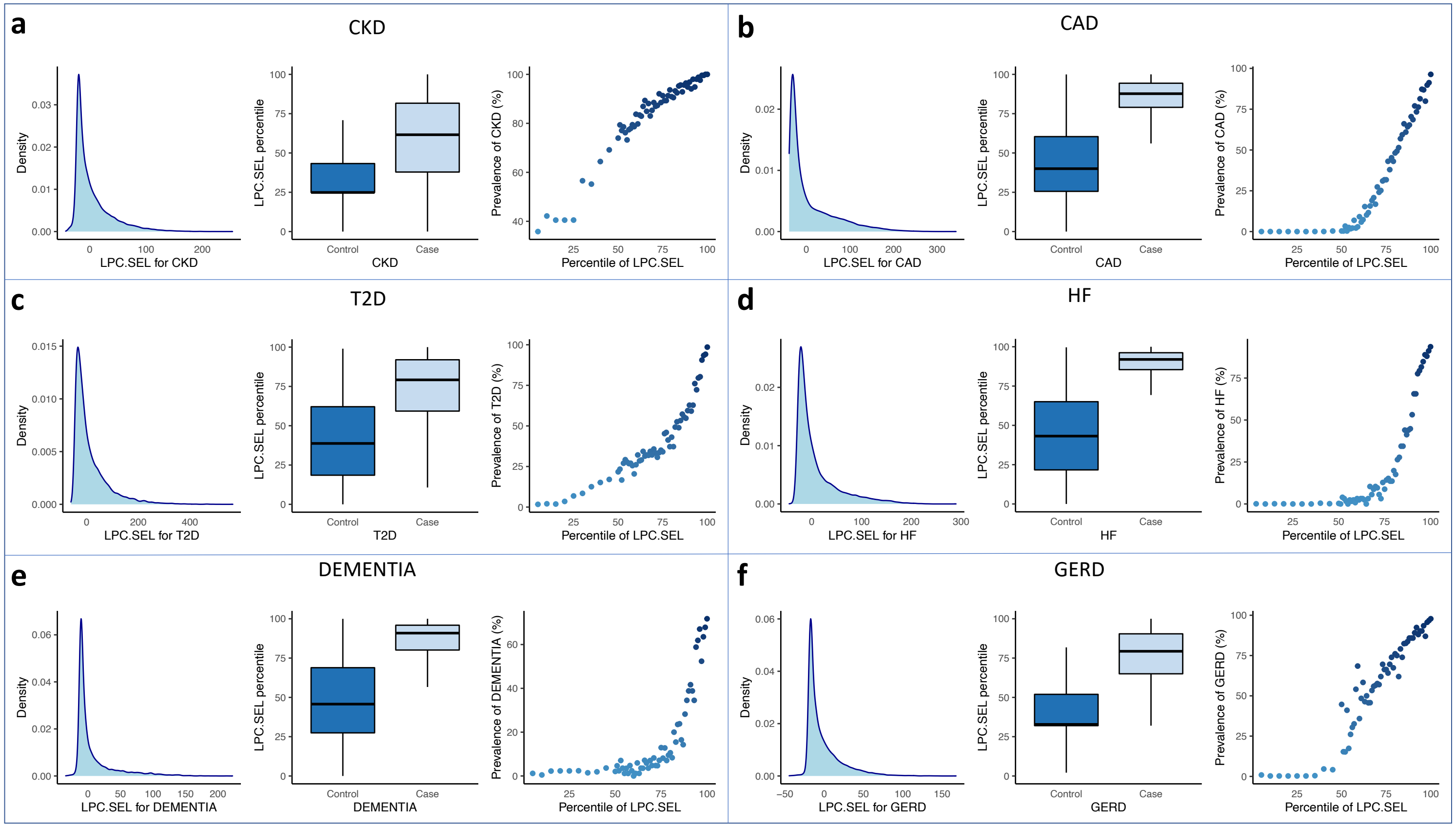

Supplementary Figure 4: **Distribution of final LPC scores for six phenotypes in the test set.** LPC risk scores are derived based on selected phecodes. Estimated density and distribution of LPC risk scores cases vs. controls for **a** CKD (cases including G1, G2, G3a/b, and G4 stages), **b** CAD, **c** T2D, **d** HF, **e** Dementia, and **f** GERD. For each phenotype: Left, distribution of LPC risk scores in the test set. Middle, LPC risk score percentiles among cases vs. controls. Right, case prevalence in 60 bins according to the percentiles of LPC risk scores.

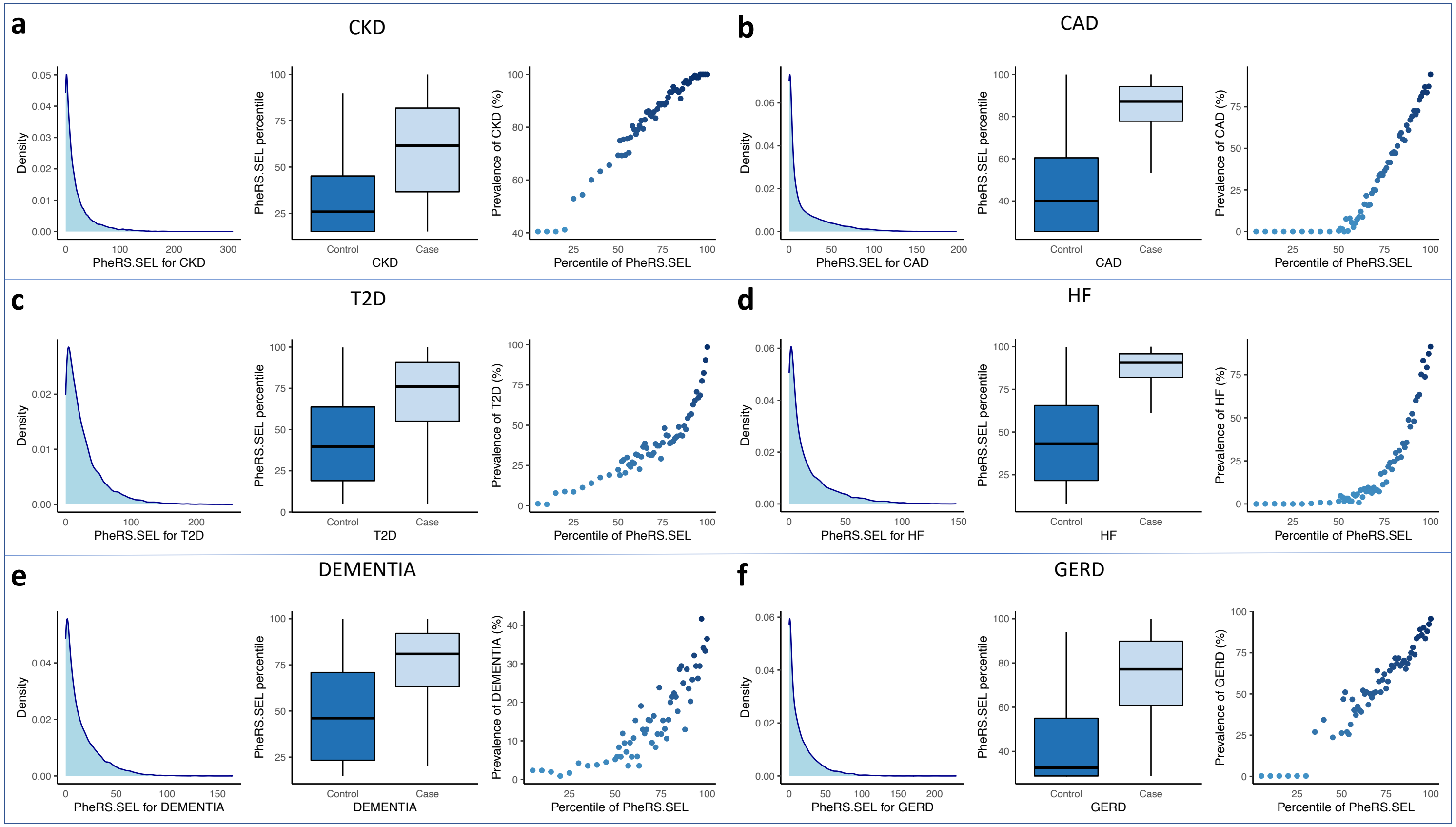

Supplementary Figure 5: **Distribution of PheRS scores for six phenotypes in the test set.** PheRS risk scores are derived based on selected phecodes. Estimated density and distribution of PheRS risk scores cases vs. controls for **a** CKD (cases including G1, G2, G3a/b, and G4 stages), **b** CAD, **c** T2D, **d** HF, **e** Dementia, and **f** GERD. For each phenotype: Left, distribution of PheRS in the test set. Middle, PheRS percentiles among cases vs. controls. Right, prevalence of phenotype in 60 bins according to the percentiles of PheRS.

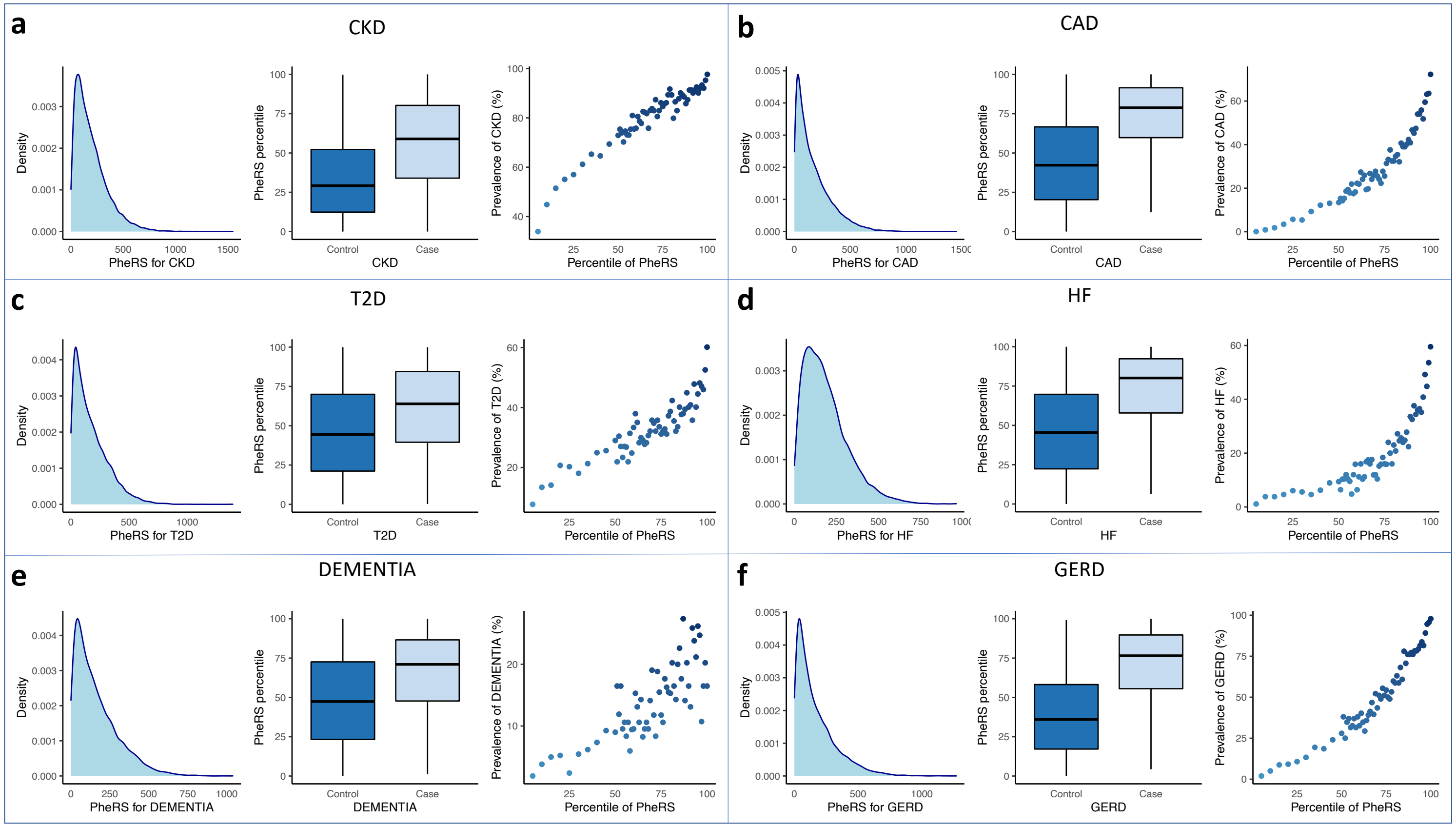

Supplementary Figure 6: **Distribution of PheRS scores for six phenotypes in the test set.** PheRS risk scores are derived based on **all** phecodes. Estimated density and distribution of PheRS risk scores cases vs. controls for **a** CKD (cases including G1, G2, G3a/b, and G4 stages), **b** CAD, **c** T2D, **d** HF, **e** Dementia, and **f** GERD. For each phenotype: Left, distribution of PheRS in the test set. Middle, PheRS percentiles among cases vs. controls. Right, prevalence of phenotype in 60 bins according to the percentiles of PheRS.

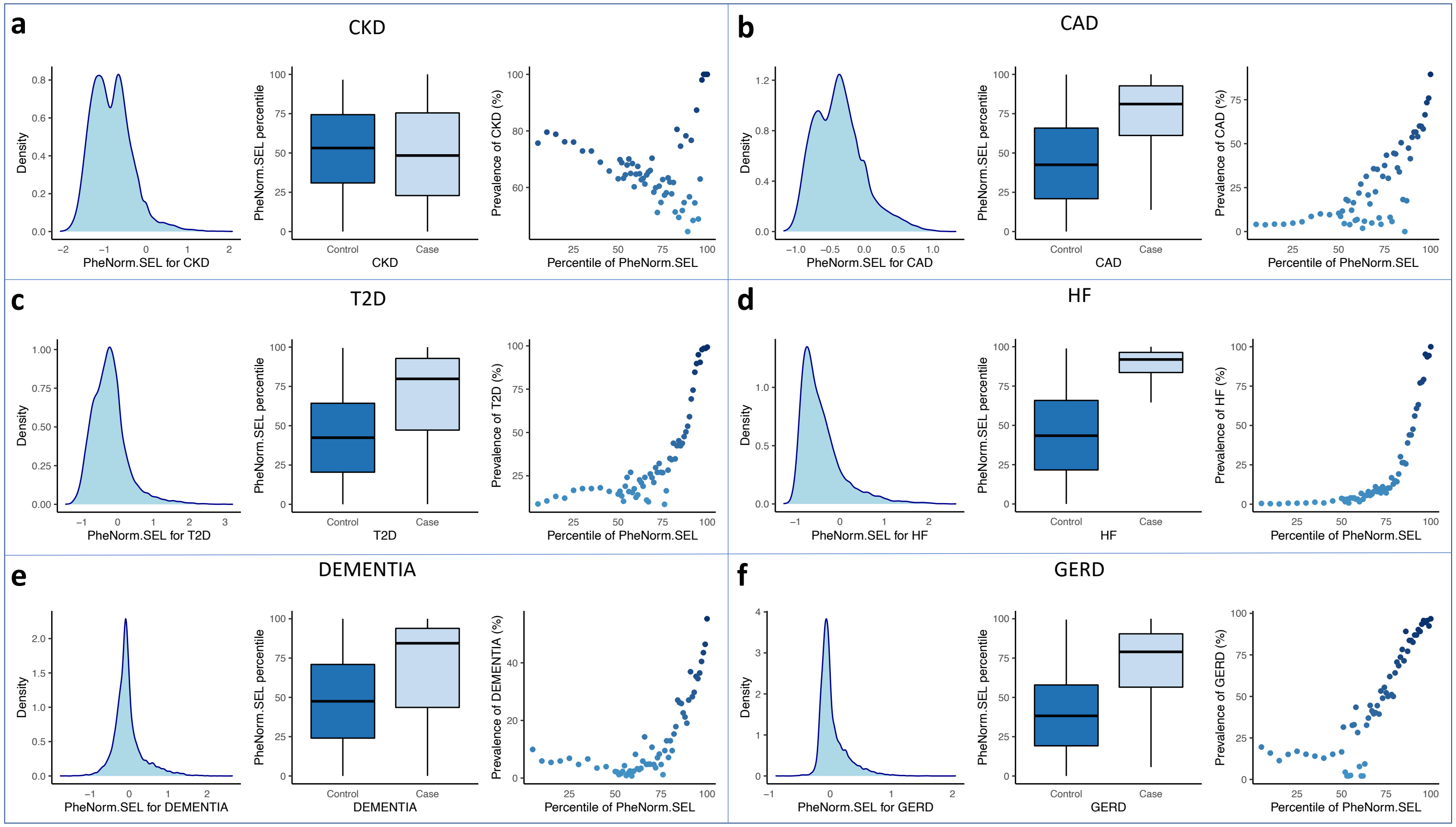

Supplementary Figure 7: **Distribution of PheNorm scores for six phenotypes in the test set.** PheNorm risk scores are derived based on selected phecodes. Estimated density and distribution of PheNorm risk scores cases vs. controls for **a** CKD (cases including G1, G2, G3a/b, and G4 stages), **b** CAD, **c** T2D, **d** HF, **e** Dementia, and **f** GERD. For each phenotype: Left, distribution of PheNorm in the test set. Middle, PheNorm percentiles among cases vs. controls. Right, prevalence of phenotype in 60 bins according to the percentiles of PheNorm.

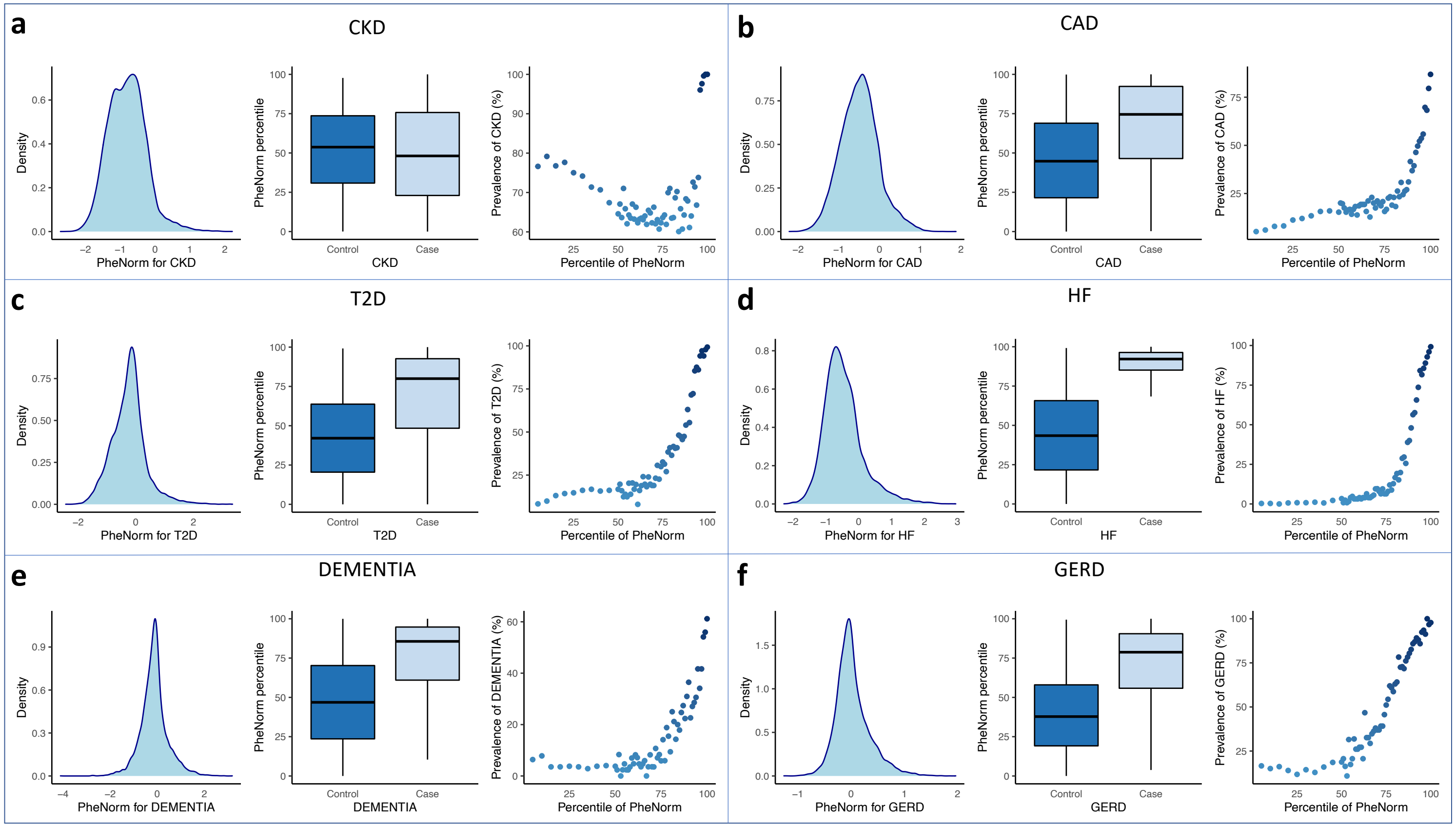

Supplementary Figure 8: **Distribution of PheNorm scores for six phenotypes in the test set.** PheNorm risk scores are derived based on **all** phecodes. Estimated density and distribution of PheNorm risk scores cases vs. controls for **a** CKD (cases including G1, G2, G3a/b, and G4 stages), **b** CAD, **c** T2D, **d** HF, **e** Dementia, and **f** GERD. For each phenotype: Left, distribution of PheNorm in the test set. Middle, PheNorm percentiles among cases vs. controls. Right, prevalence of phenotype in 60 bins according to the percentiles of PheNorm.

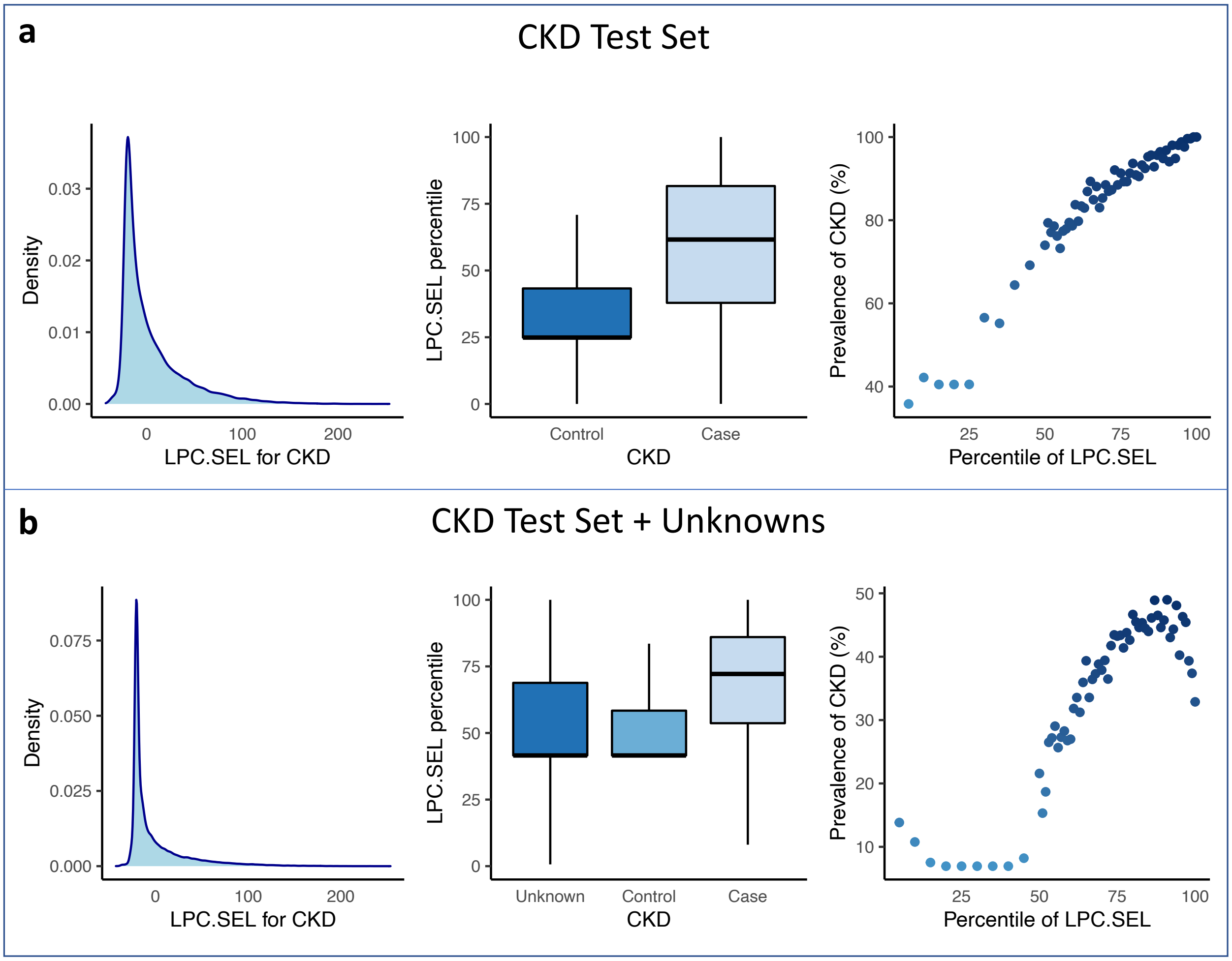

Supplementary Figure 9: **Distribution of CKD LPC risk scores in the test set vs. test set + individuals with unknown status.** Estimated density and distribution of LPC risk scores, **a** cases vs. controls in CKD test set, and **b** cases vs. controls vs. unknowns in CKD test set and individuals with unknown status. LPC risk scores are derived based on **pre-selected** phecodes. Left, distribution of LPC risk scores. Middle, LPC risk score percentiles among cases vs. controls. Right, prevalence of phenotype in 60 bins according to the percentiles of LPC risk scores.

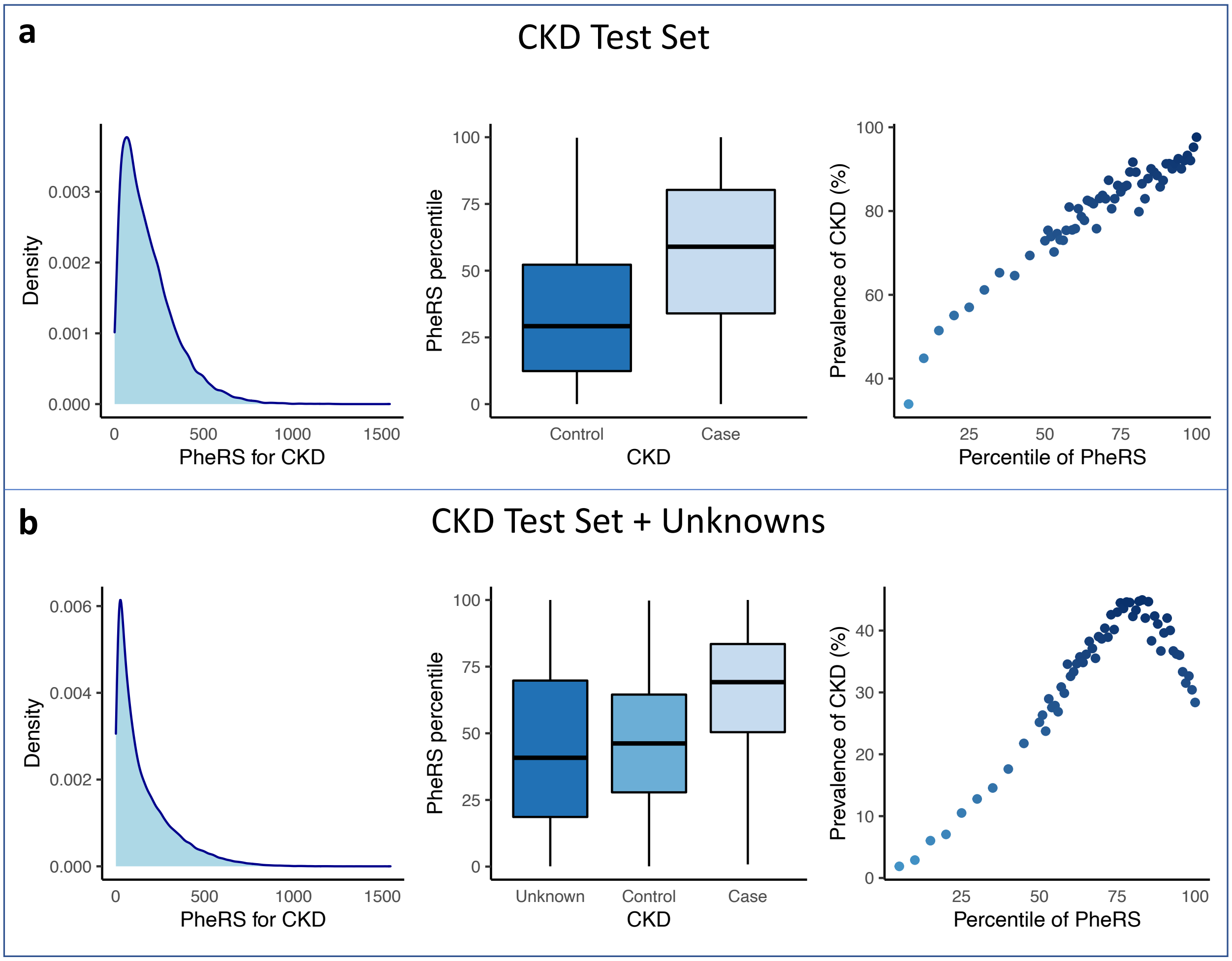

Supplementary Figure 10: **Distribution of CKD PheRS risk scores in the test set vs. test set + individuals with unknown status.** Estimated density and distribution of PheRS risk scores, **a** cases vs. controls in CKD test set, and **b** cases vs. controls vs. unknowns in CKD test set and individuals with unknown status. PheRS risk scores are derived based on **all** phecodes. Left, distribution of PheRS risk scores. Middle, PheRS risk score percentiles among cases vs. controls. Right, prevalence of phenotype in 60 bins according to the percentiles of PheRS risk scores.

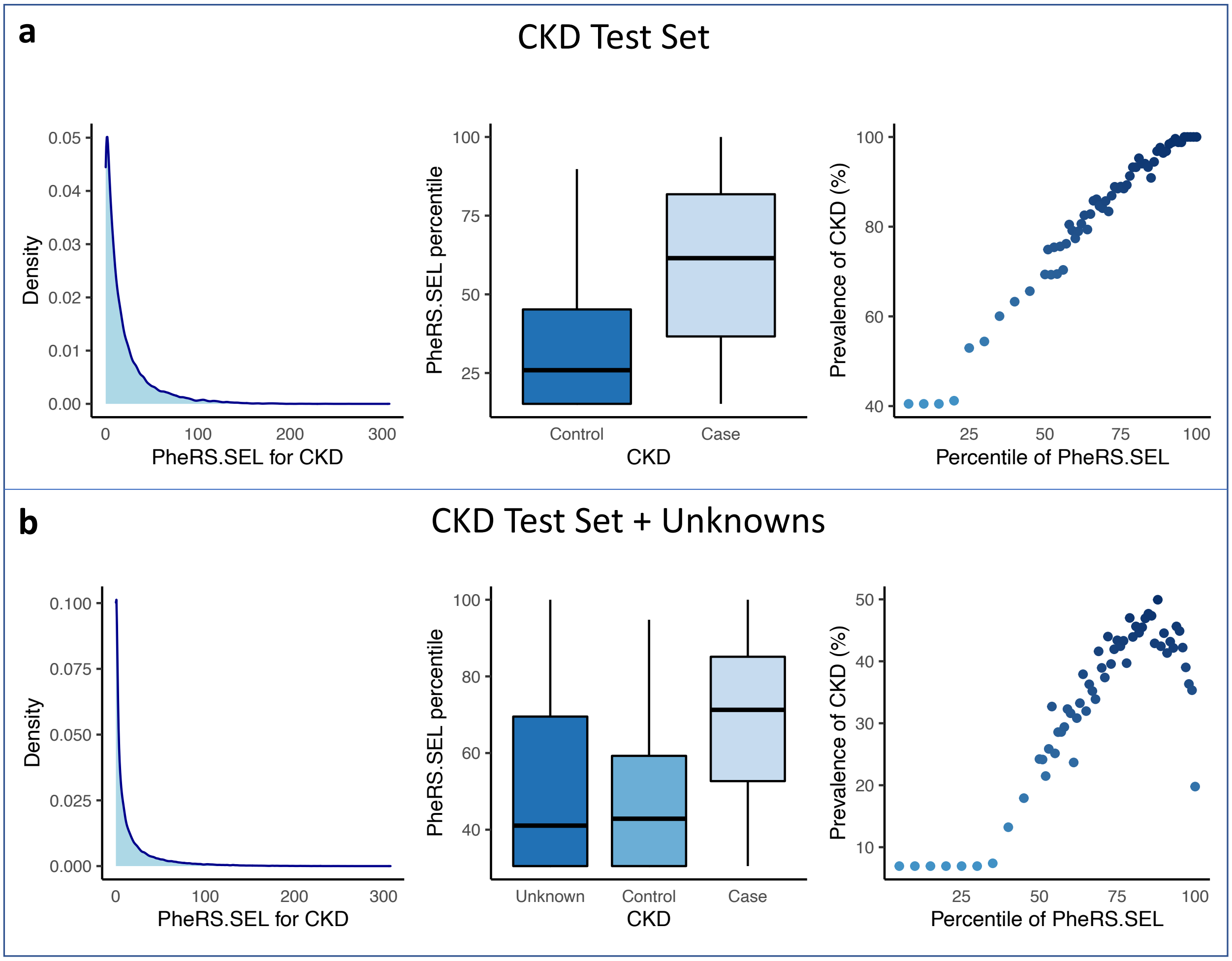

Supplementary Figure 11: **Distribution of CKD PheRS risk scores in the test set vs. test set + individuals with unknown status.** Estimated density and distribution of PheRS risk scores, **a** cases vs. controls in CKD test set, and **b** cases vs. controls vs. unknowns in CKD test set and individuals with unknown status. PheRS risk scores are derived based on **pre-selected** phecodes. Left, distribution of PheRS risk scores. Middle, PheRS risk score percentiles among cases vs. controls. Right, prevalence of phenotype in 60 bins according to the percentiles of PheRS risk scores.

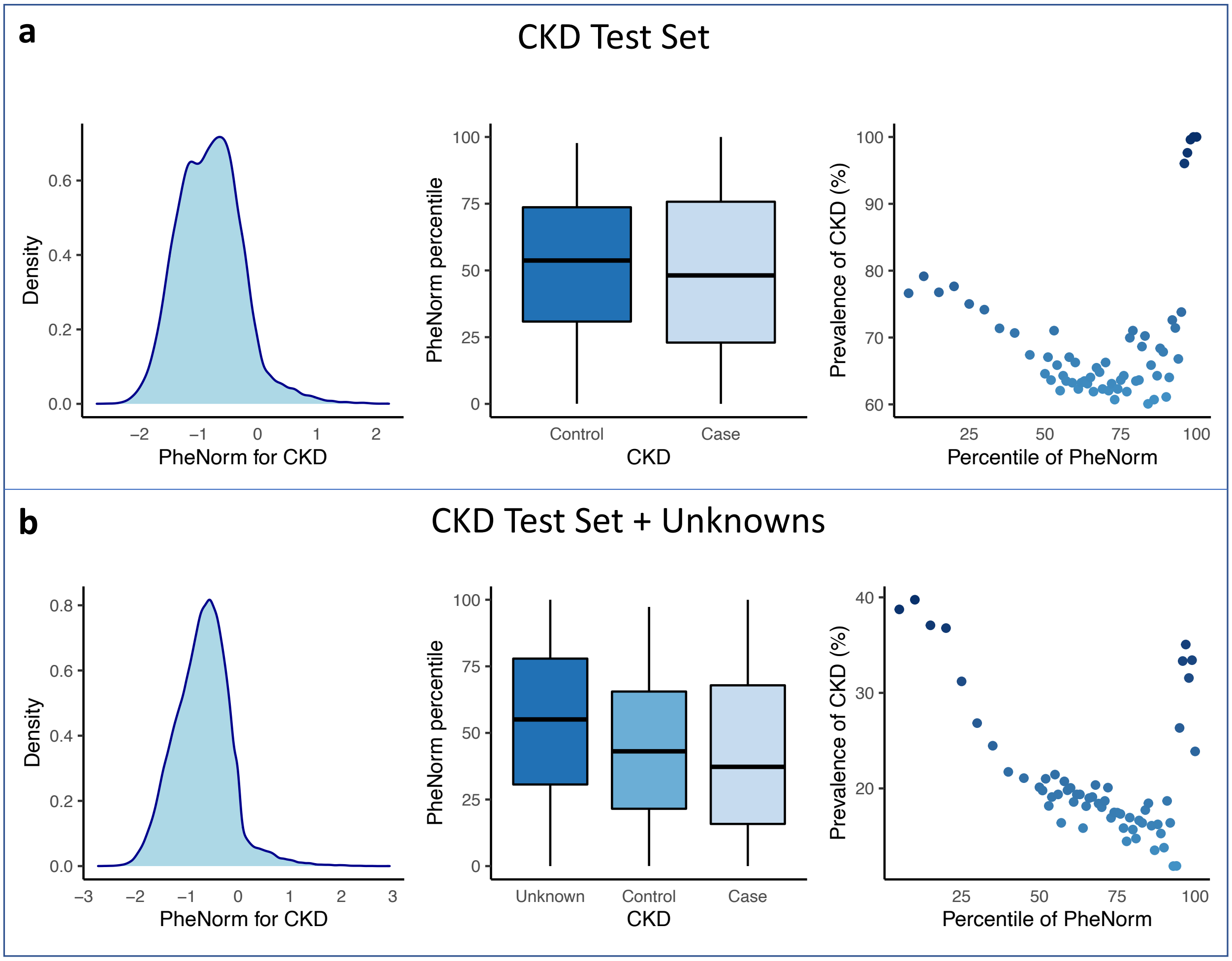

Supplementary Figure 12: **Distribution of CKD PheNorm risk scores in the test set vs. test set + individuals with unknown status.** Estimated density and distribution of PheNorm risk scores, **a** cases vs. controls in CKD test set, and **b** cases vs. controls vs. unknowns in CKD test set and individuals with unknown status. PheNorm risk scores are derived based on **all** phecodes. Left, distribution of PheNorm risk scores. Middle, PheNorm risk score percentiles among cases vs. controls. Right, prevalence of phenotype in 60 bins according to the percentiles of PheNorm risk scores.

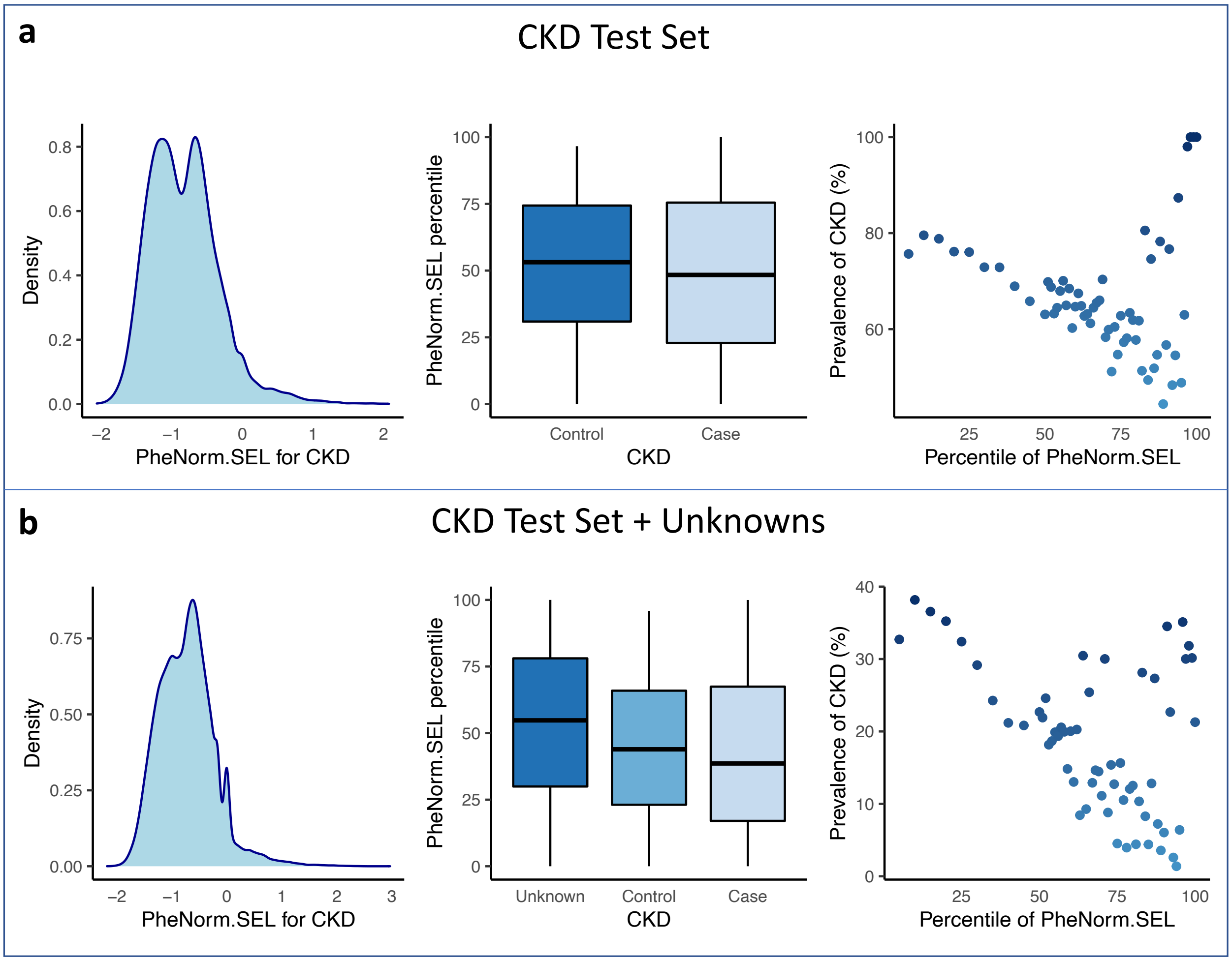

Supplementary Figure 13: **Distribution of CKD PheNorm risk scores in the test set vs. test set + individuals with unknown status.** Estimated density and distribution of PheNorm risk scores, **a** cases vs. controls in CKD test set, and **b** cases vs. controls vs. unknowns in CKD test set and individuals with unknown status. PheNorm risk scores are derived based on **pre-selected** phecodes. Left, distribution of PheNorm risk scores. Middle, PheNorm risk score percentiles among cases vs. controls. Right, prevalence of phenotype in 60 bins according to the percentiles of PheNorm risk scores.

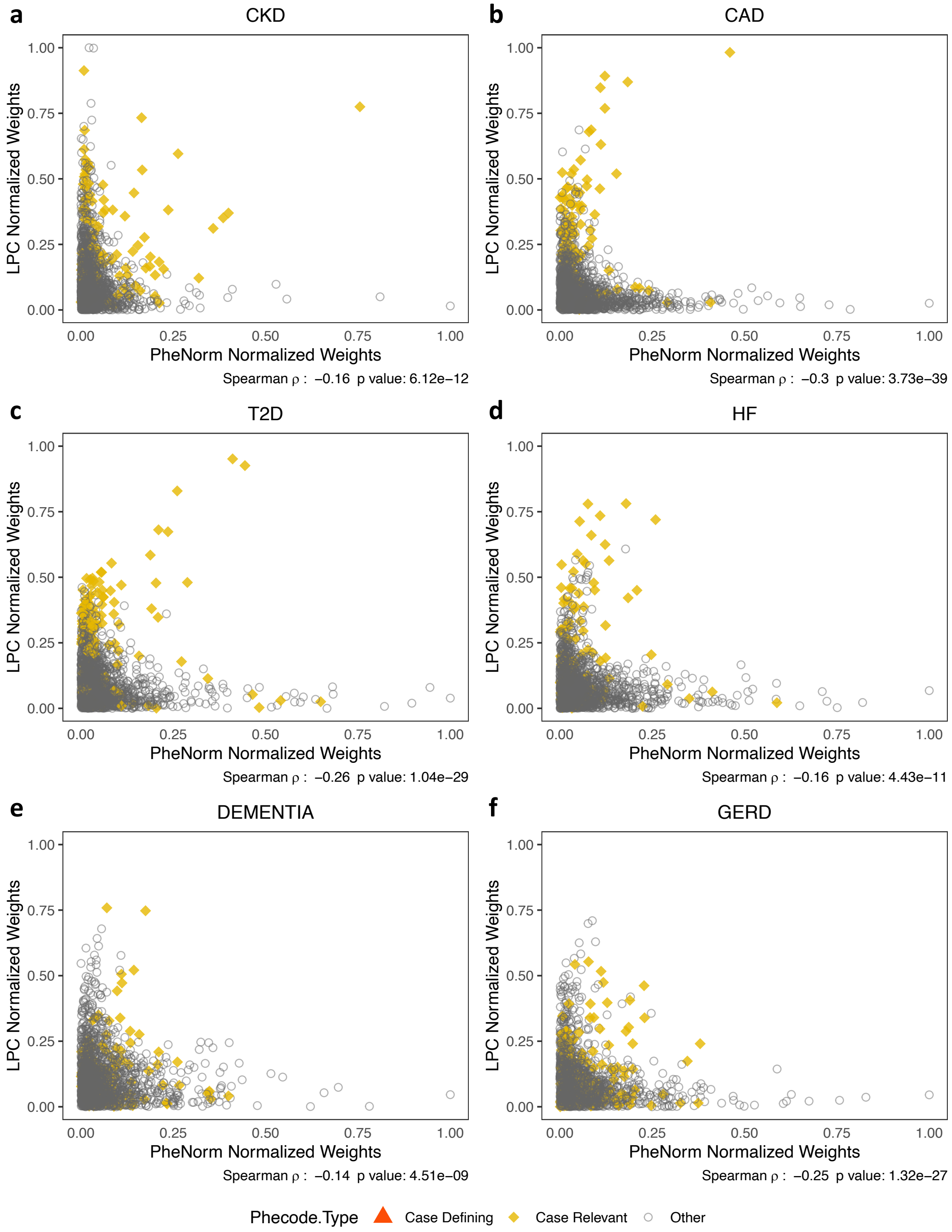

Supplementary Figure 14: **Weights for all phecodes used to build PheNorm and LPC for 6 phenotypes.** Scatter plots of weights for **a** CKD (cases including G1, G2, G3a/b, and G4 stages), **b** CAD, **c** T2D, **d** HF, **e** Dementia, and **f** GERD. The weights for the case defining and pre-selected phecodes are highlighted.

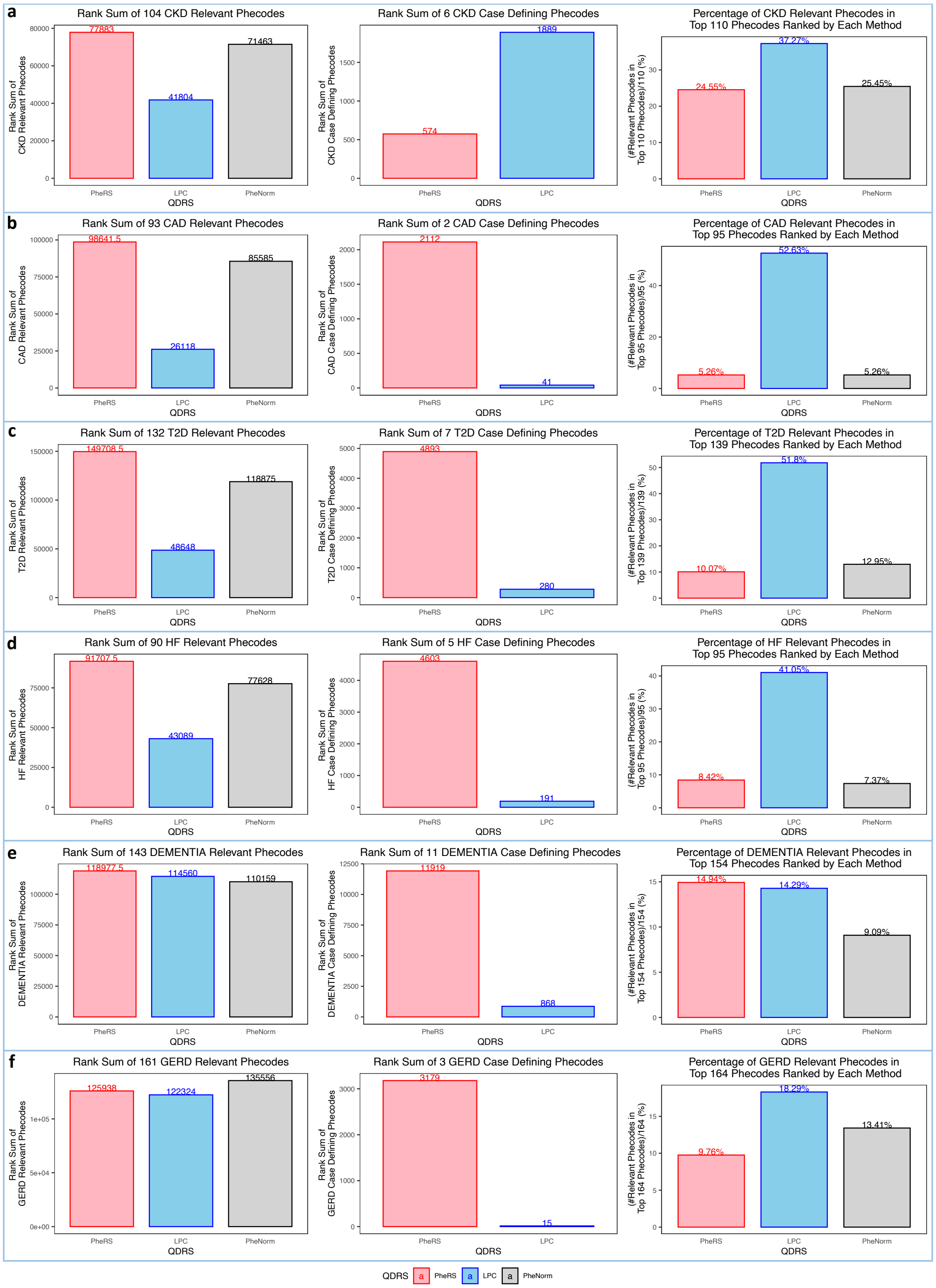

Supplementary Figure 15: **Rank sum of relevant phecodes (excluding case defining phecodes), case defining phecodes and percentage of relevant phecodes among top phecodes ranked by PheRS, LPC and PheNorm. a** CKD, **b** CAD, **c** T2D, **d** HF, **e** Dementia, and **f** GERD.

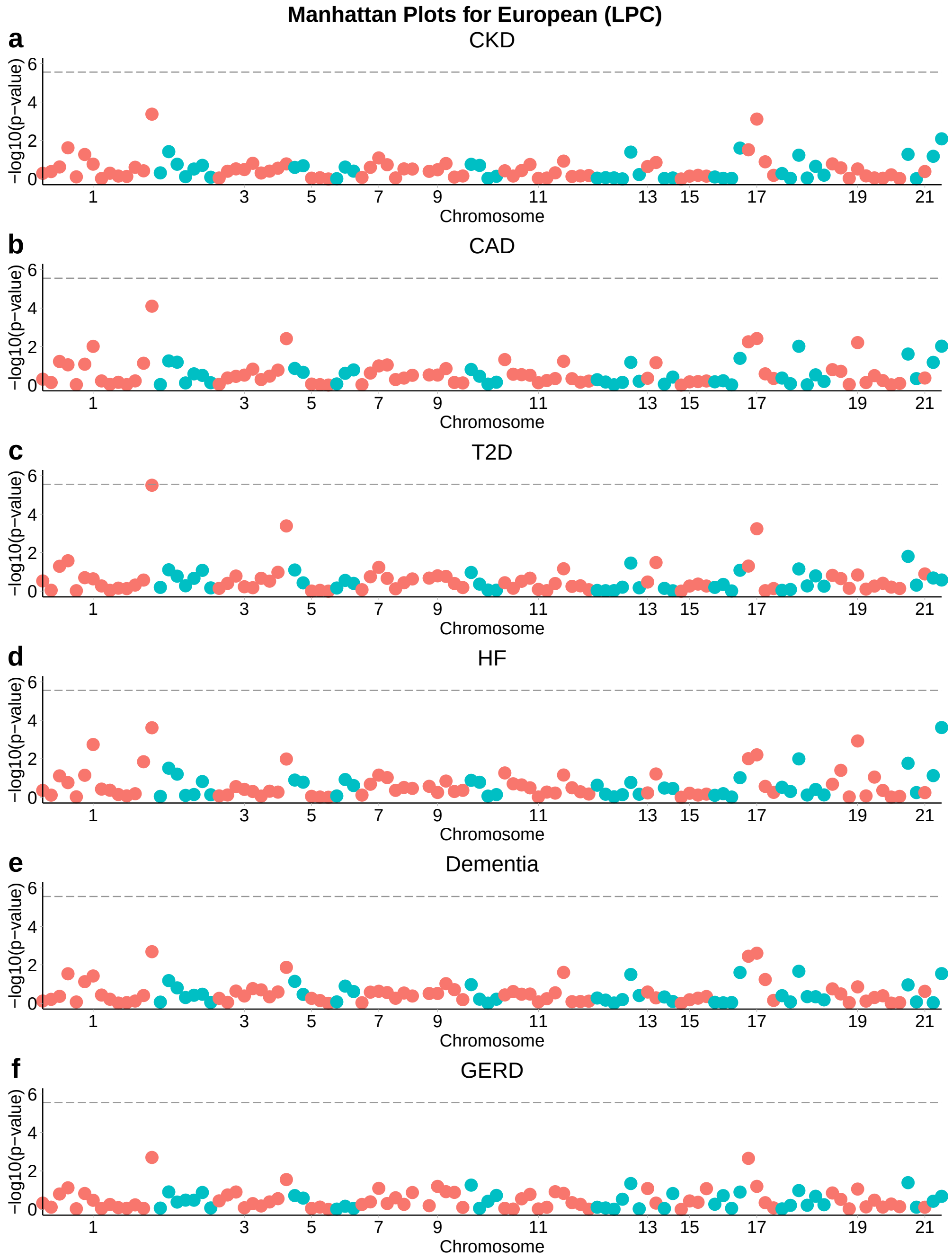

Supplementary Figure 16: **Gene-based test results for the European dataset for 107 autosomal genes on the eMERGE-seq panel using LPC with all phecodes for six diseases.** Manhattan plots for **a** CKD, **b** CAD, **c** T2D, **d** HF, **e** Dementia, and **f** GERD. The horizontal line corresponds to exome-wide significance level.

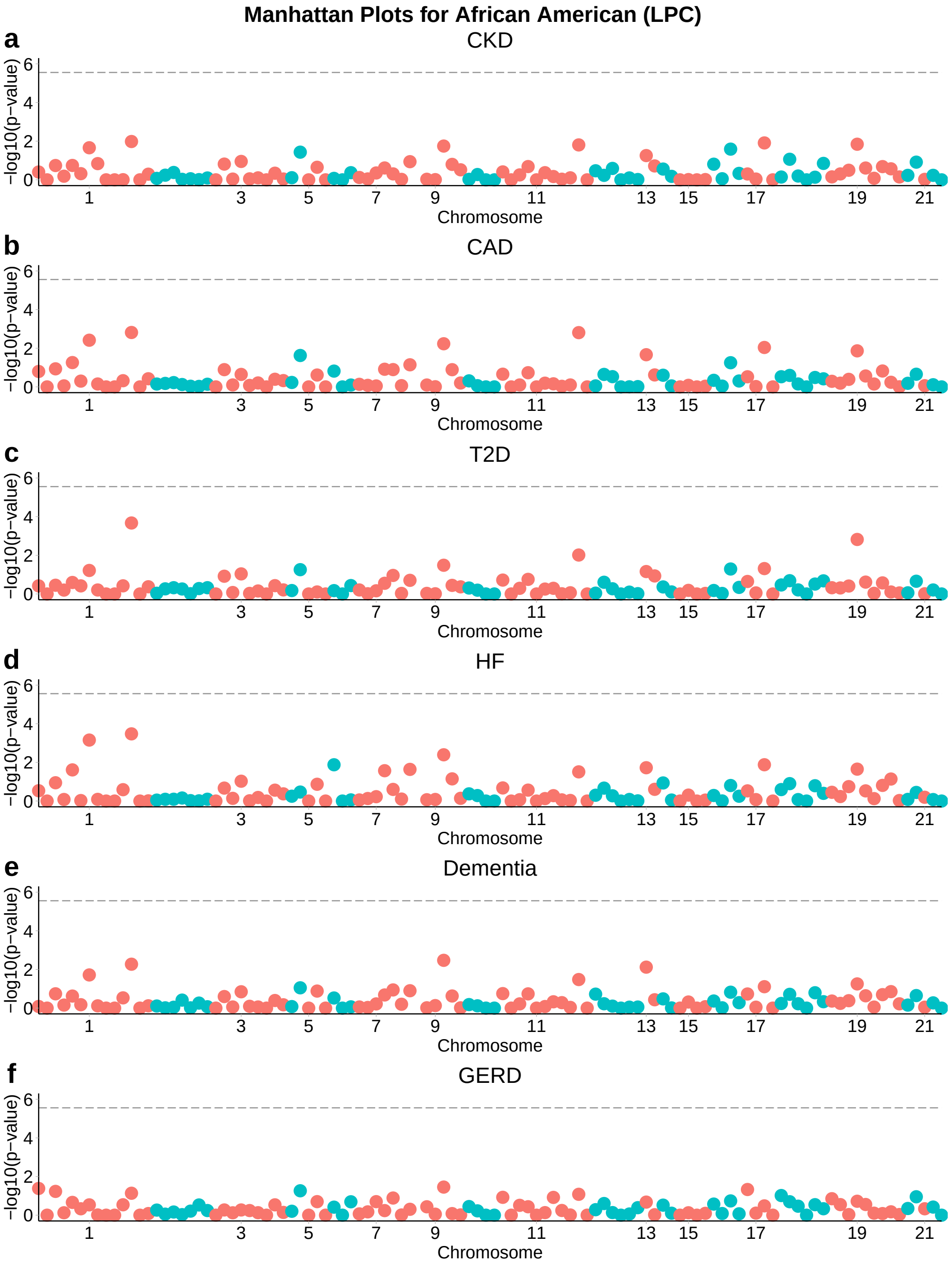

Supplementary Figure 17: **Gene-based test results for the African American dataset for 107 autosomal genes on the eMERGE-seq panel using LPC with all phecodes for six diseases.** Manhattan plots for **a** CKD, **b** CAD, **c** T2D, **d** HF, **e** Dementia, and **f** GERD. The horizontal line corresponds to exome-wide significance level.

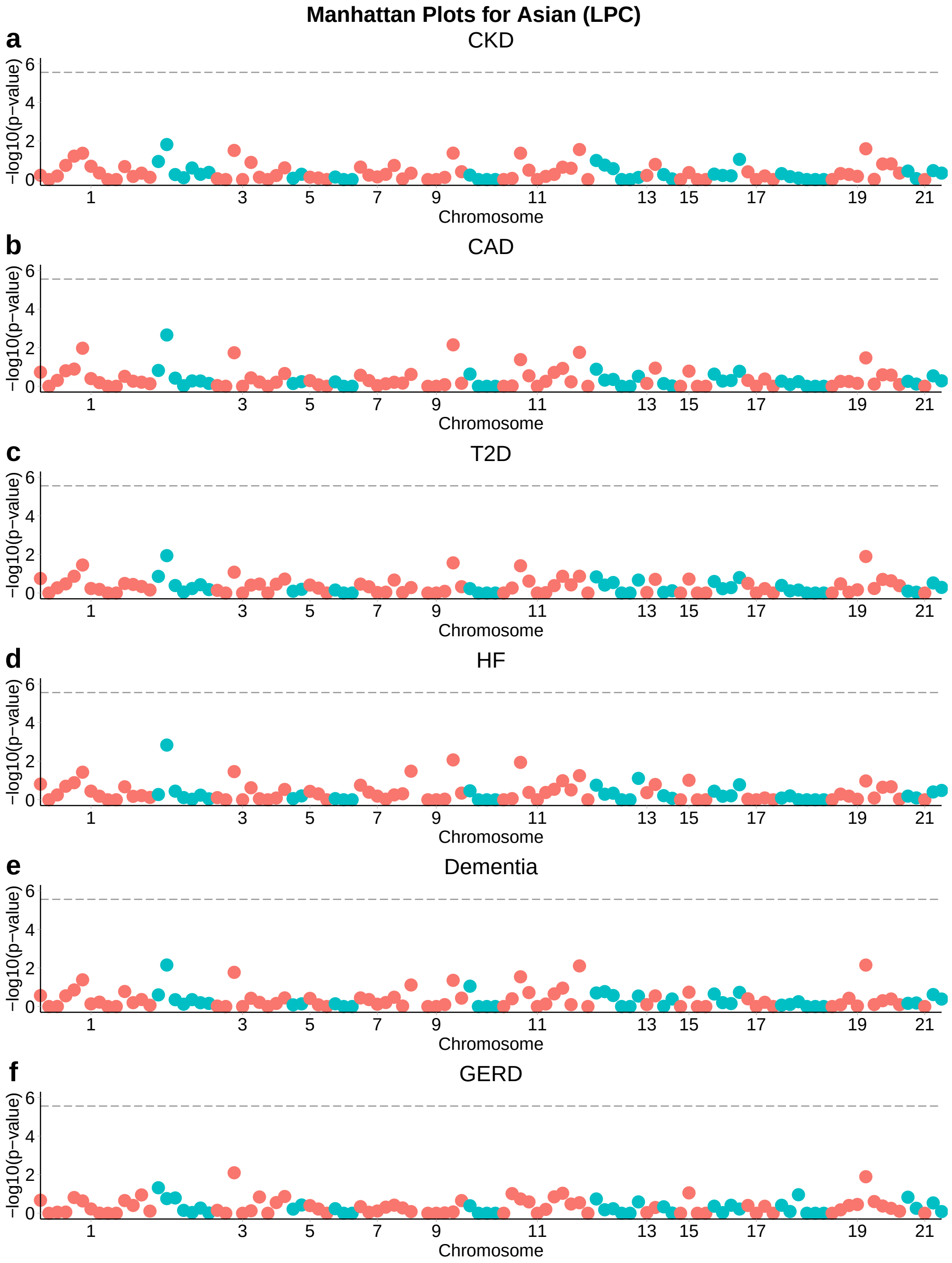

Supplementary Figure 18: **Gene-based test results for the Asian dataset for 107 autosomal genes on the eMERGE-seq panel using LPC with all phecodes for six diseases.** Manhattan plots for **a** CKD, **b** CAD, **c** T2D, **d** HF, **e** Dementia, and **f** GERD. The horizontal line corresponds to exome-wide significance level.

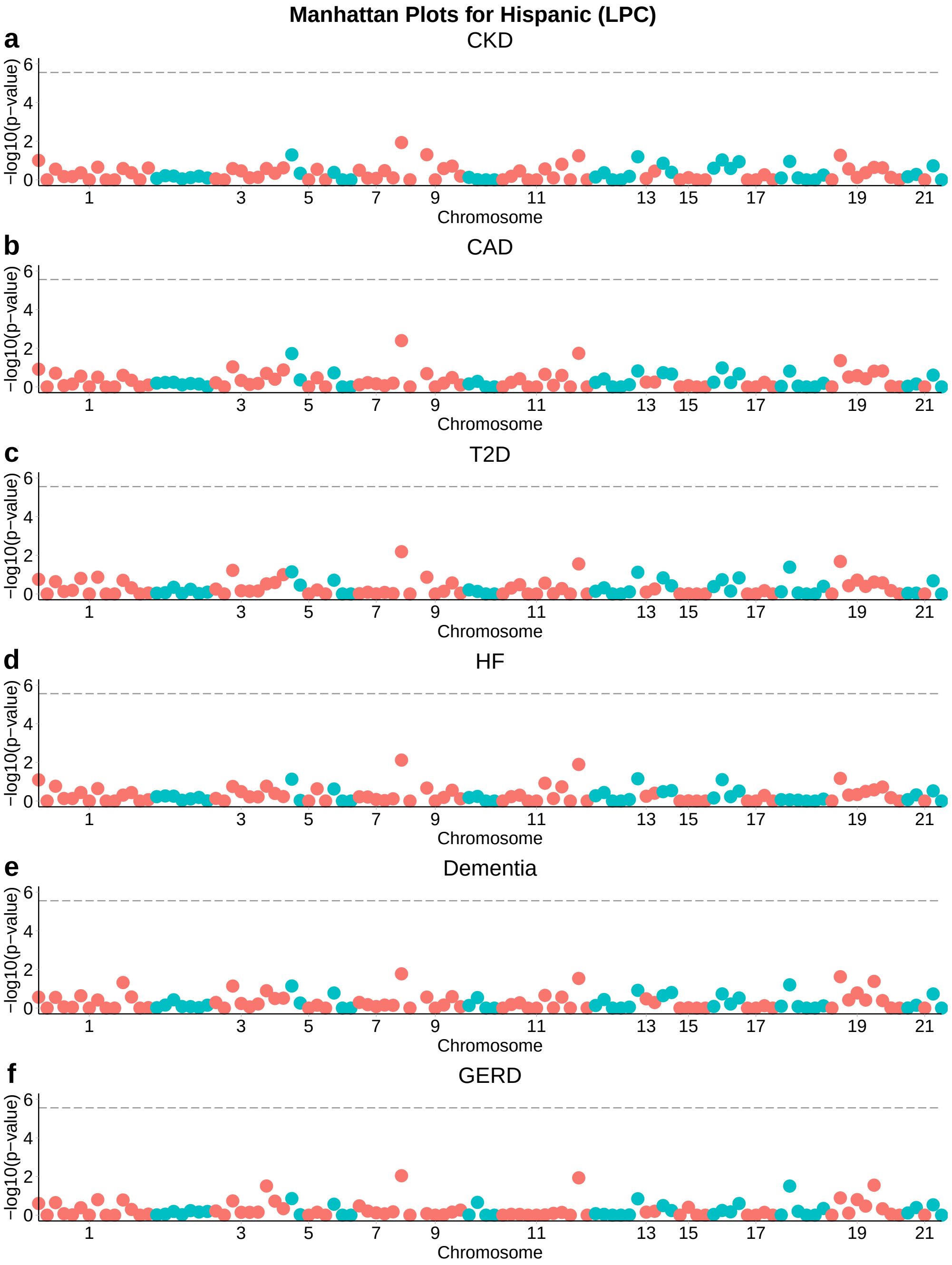

Supplementary Figure 19: **Gene-based test results for the Hispanic dataset for 107 autosomal genes on the eMERGE-seq panel using LPC with all phecodes for six diseases.** Manhattan plots for **a** CKD, **b** CAD, **c** T2D, **d** HF, **e** Dementia, and **f** GERD. The horizontal line corresponds to exome-wide significance level.

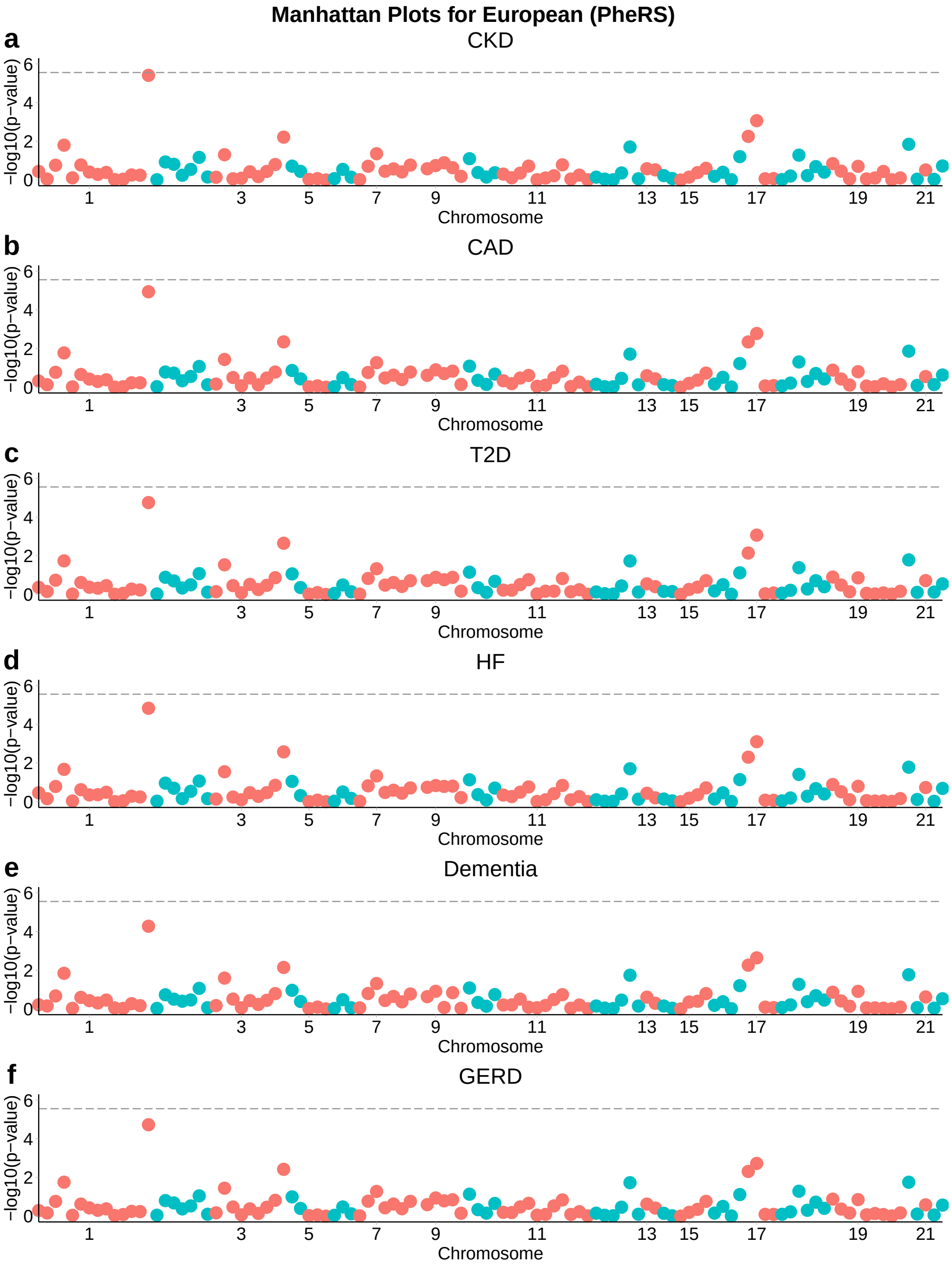

Supplementary Figure 20: **Gene-based test results for the European dataset for 107 autosomal genes on the eMERGE-seq panel using PheRS with all phecodes for six diseases.** Manhattan plots for **a** CKD, **b** CAD, **c** T2D, **d** HF, **e** Dementia, and **f** GERD. The horizontal line corresponds to exome-wide significance level.

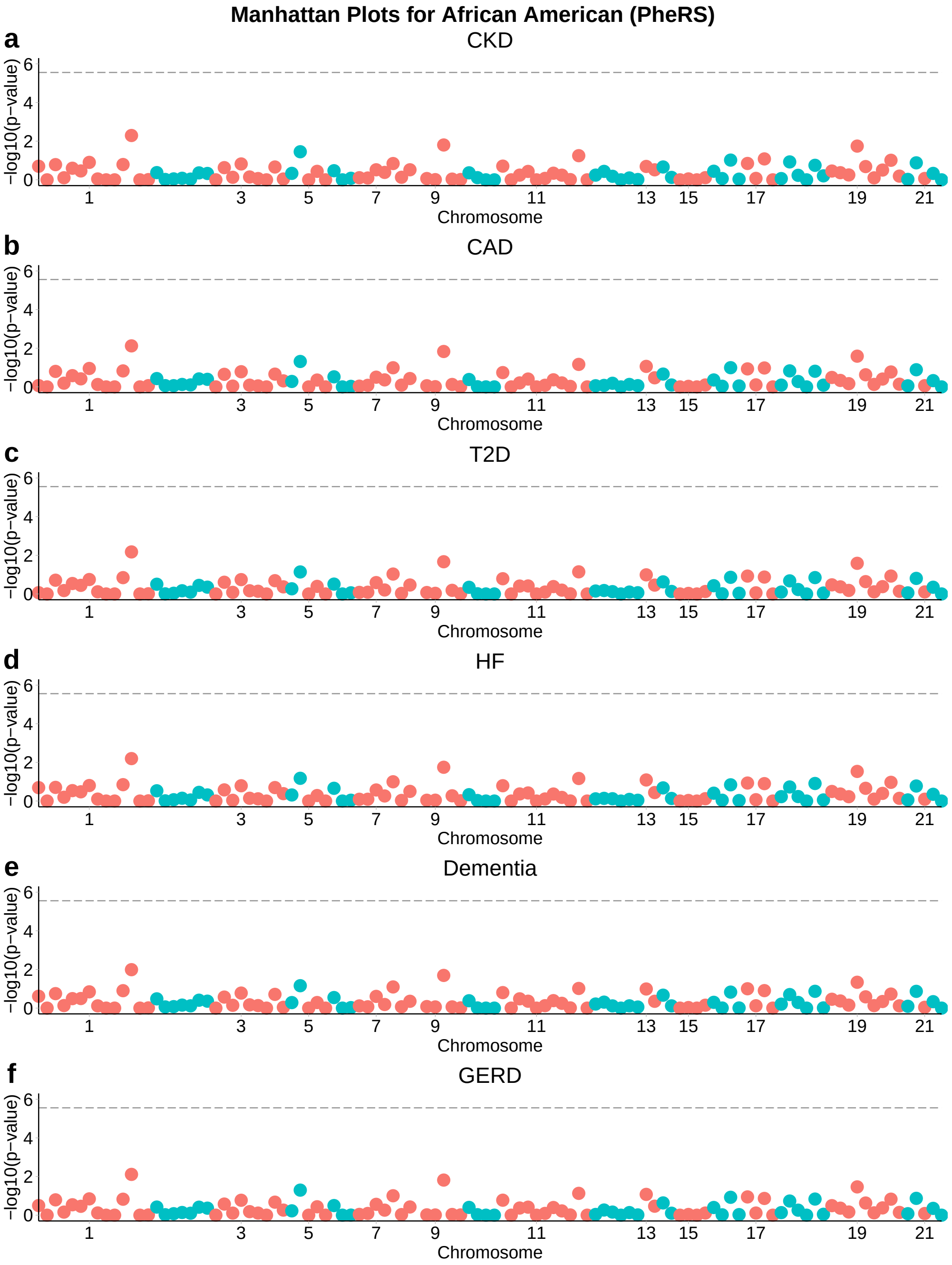

Supplementary Figure 21: **Gene-based test results for the African American dataset for 107 autosomal genes on the eMERGE-seq panel using PheRS with all phecodes for six diseases.** Manhattan plots for **a** CKD, **b** CAD, **c** T2D, **d** HF, **e** Dementia, and **f** GERD. The horizontal line corresponds to exome-wide significance level.

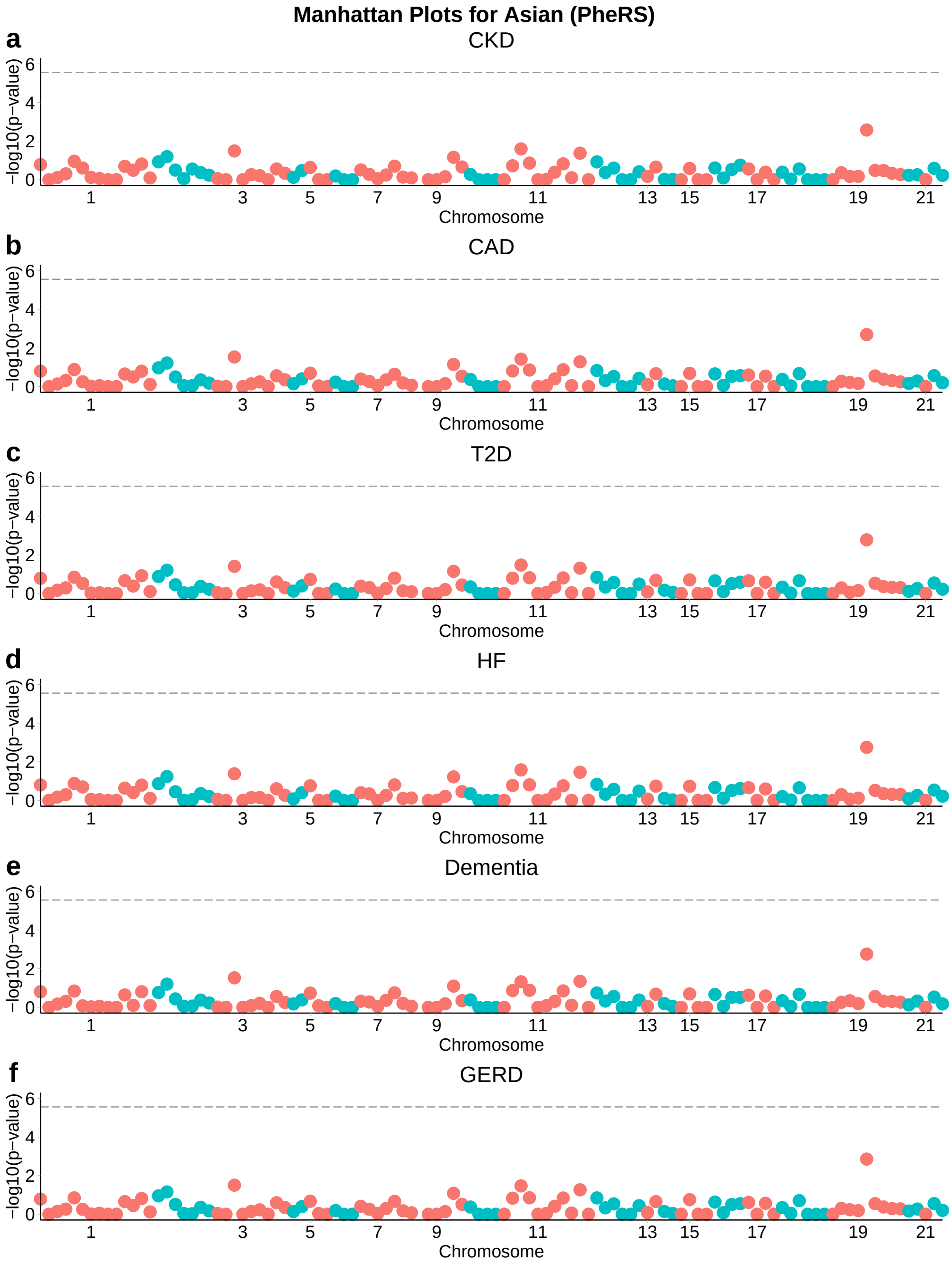

Supplementary Figure 22: **Gene-based test results for the Asian dataset for 107 autosomal genes on the eMERGE-seq panel using PheRS with all phecodes for six diseases.** Manhattan plots for **a** CKD, **b** CAD, **c** T2D, **d** HF, **e** Dementia, and **f** GERD. The horizontal line corresponds to exome-wide significance level.

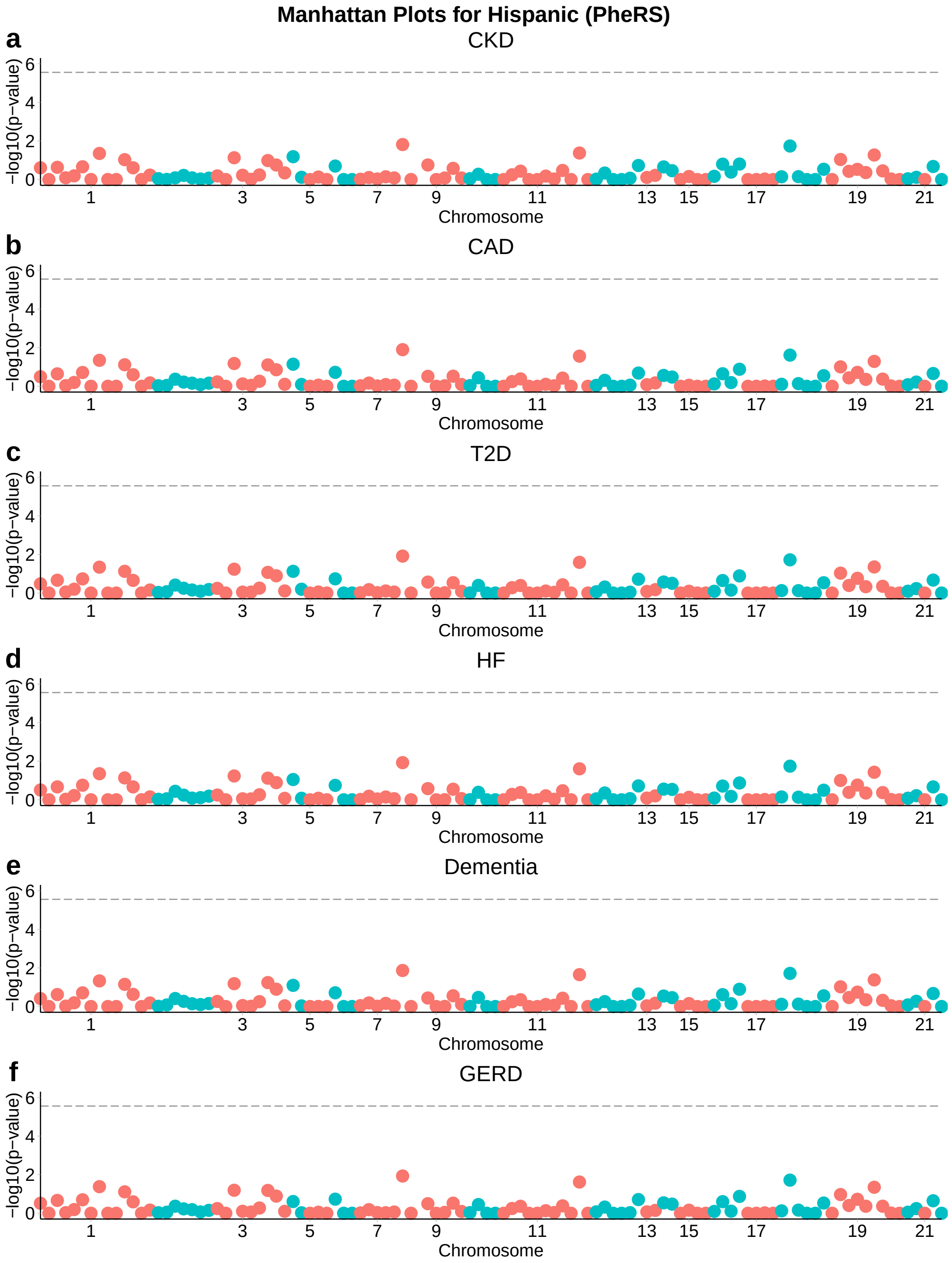

Supplementary Figure 23: **Gene-based test results for the Hispanic dataset for 107 autosomal genes on the eMERGE-seq panel using PheRS with all phecodes for six diseases.** Manhattan plots for **a** CKD, **b** CAD, **c** T2D, **d** HF, **e** Dementia, and **f** GERD. The horizontal line corresponds to exome-wide significance level.

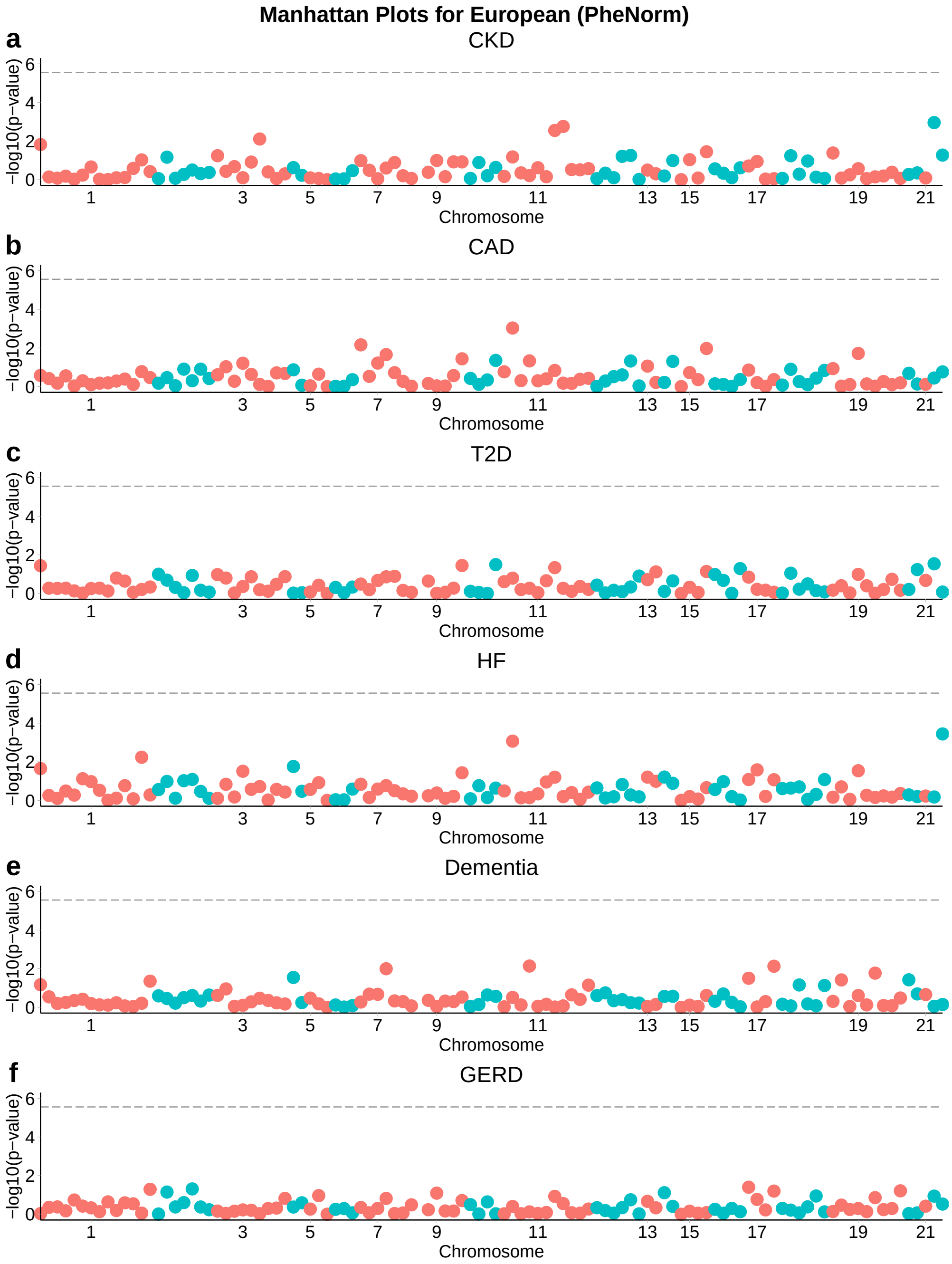

Supplementary Figure 24: **Gene-based test results for the European dataset for 107 autosomal genes on the eMERGE-seq panel using PheNorm with all phecodes for six diseases.** Manhattan plots for **a** CKD, **b** CAD, **c** T2D, **d** HF, **e** Dementia, and **f** GERD. The horizontal line corresponds to exome-wide significance level.

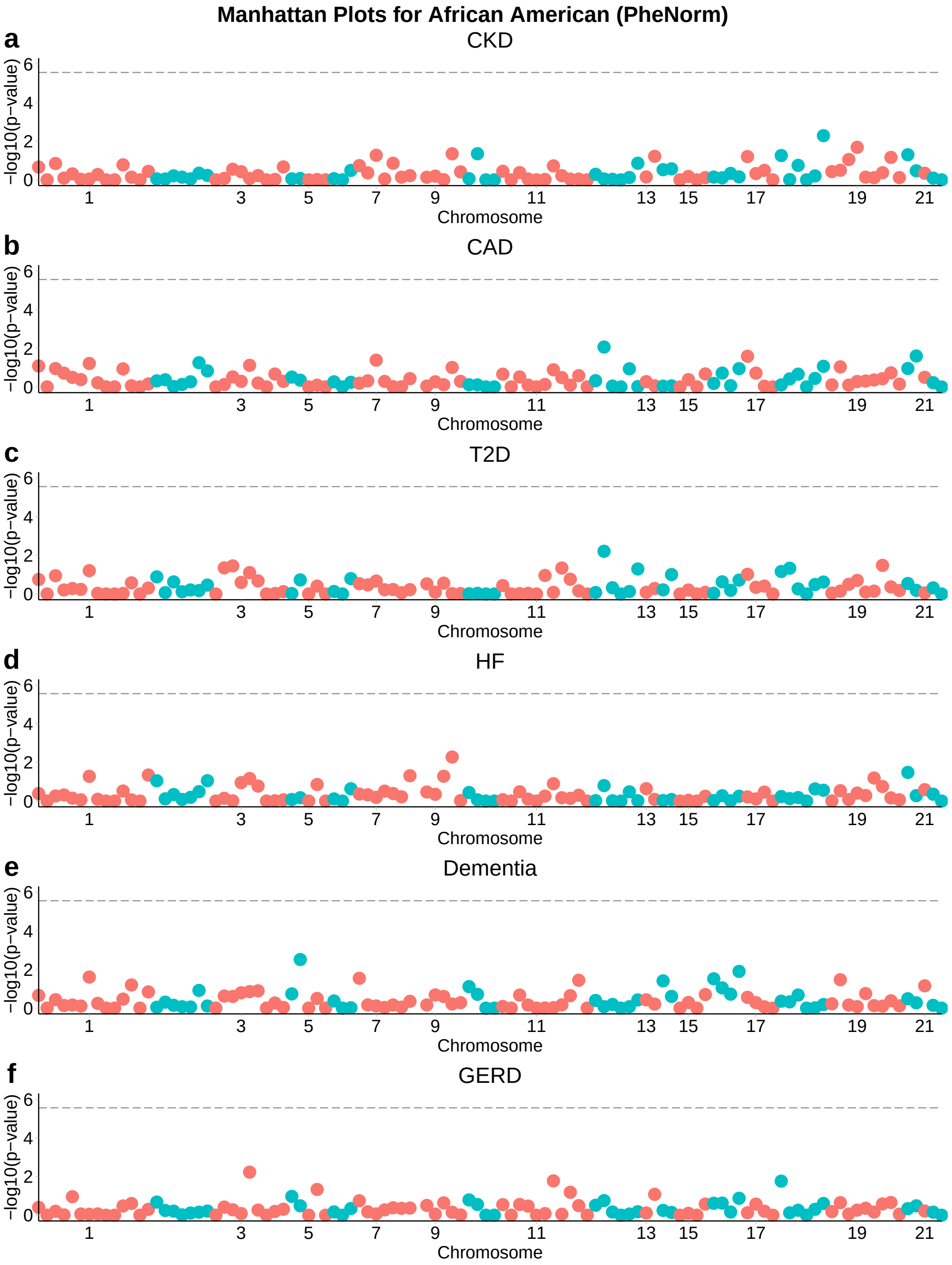

Supplementary Figure 25: **Gene-based test results for the African American for 107 autosomal genes on the eMERGE-seq panel using PheNorm with all phecodes for six diseases.** Manhattan plots for **a** CKD, **b** CAD, **c** T2D, **d** HF, **e** Dementia, and **f** GERD. The horizontal line corresponds to exome-wide significance level.

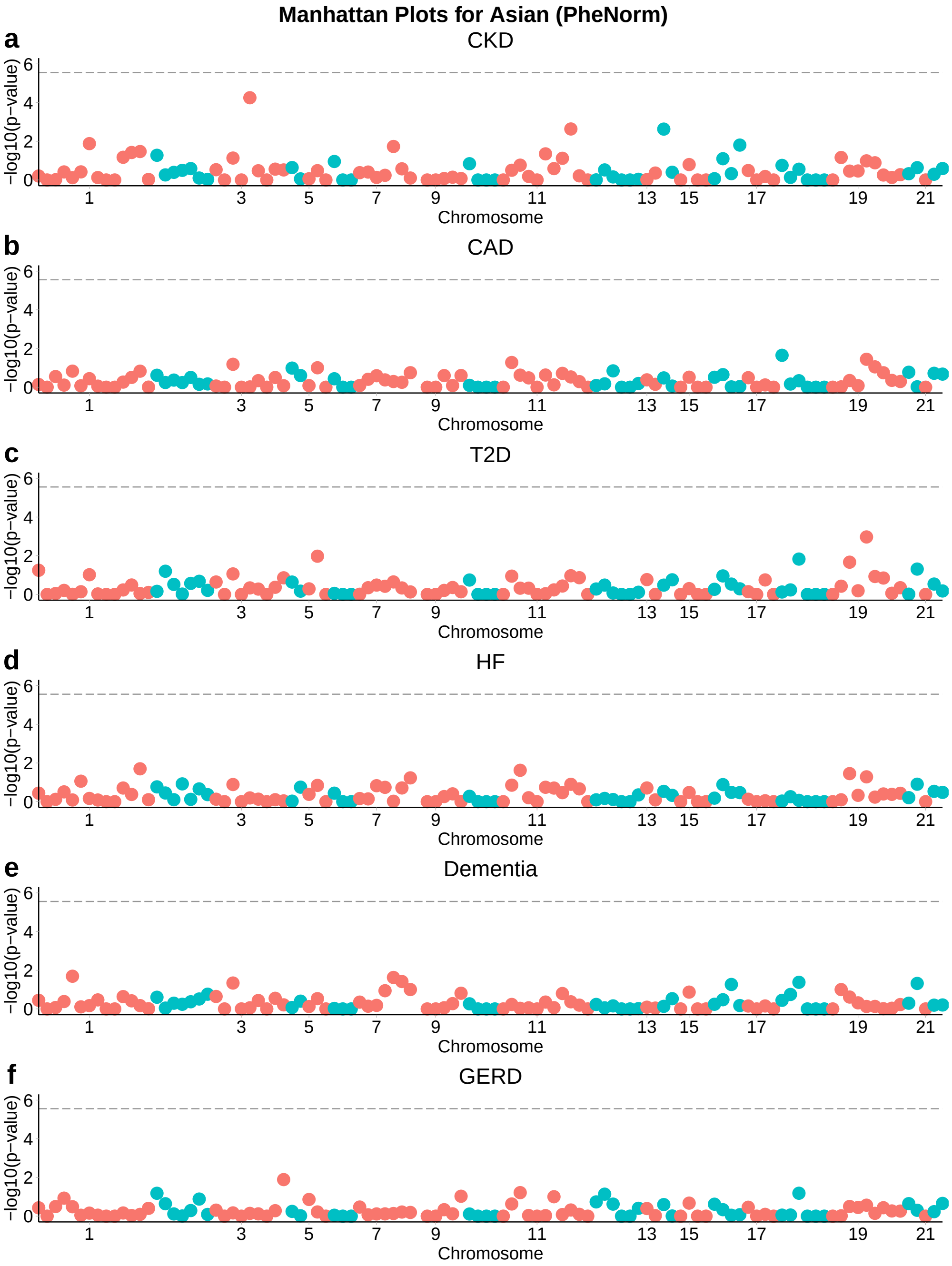

Supplementary Figure 26: **Gene-based test results for the Asian dataset for 107 autosomal genes on the eMERGE-seq panel using PheNorm with all phecodes for six diseases.** Manhattan plots for **a** CKD, **b** CAD, **c** T2D, **d** HF, **e** Dementia, and **f** GERD. The horizontal line corresponds to exome-wide significance level.

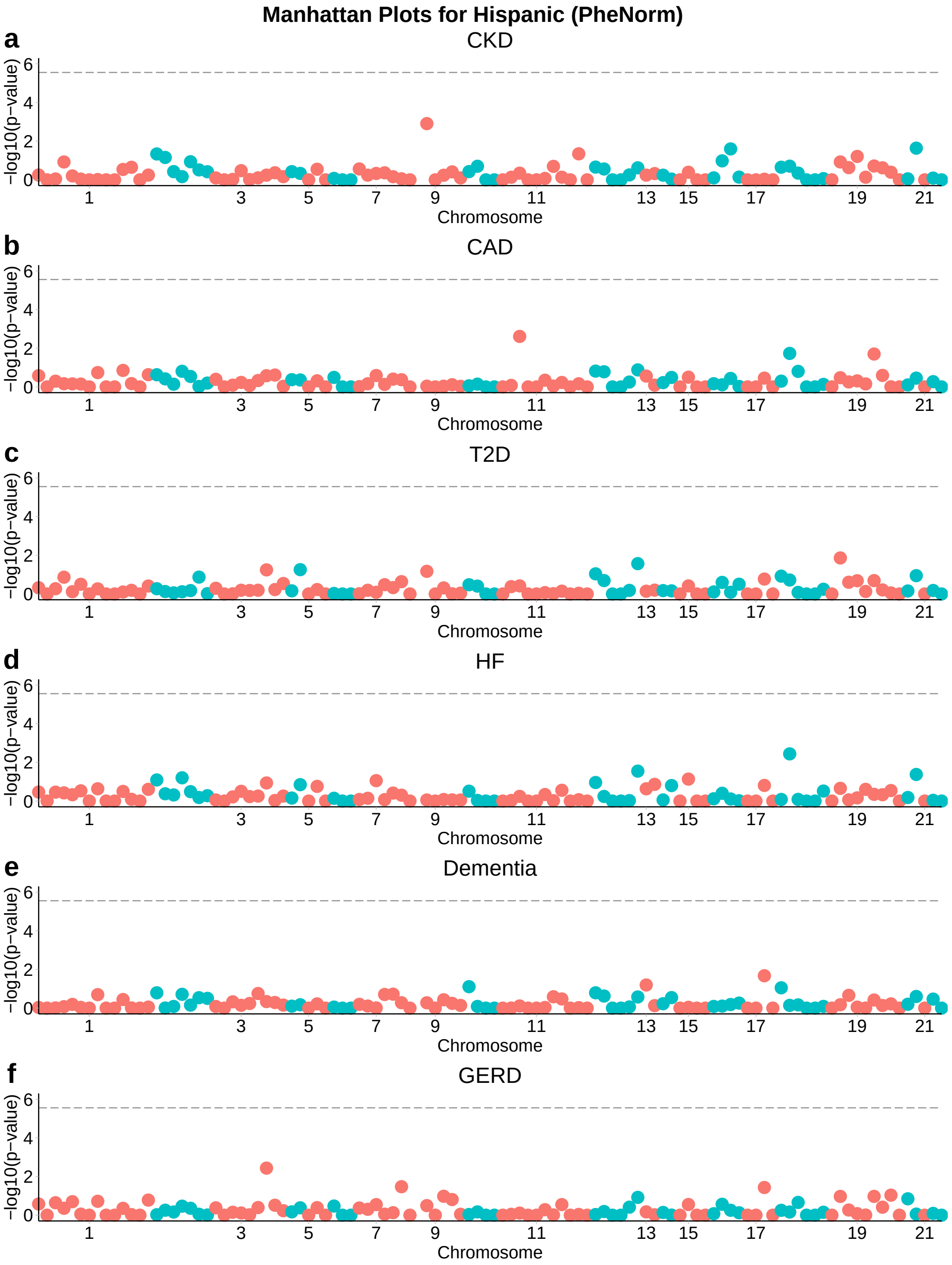

Supplementary Figure 27: **Gene-based test results for the Hispanic dataset for 107 autosomal genes on the eMERGE-seq panel using PheNorm with all phecodes for six diseases.** Manhattan plots for **a** CKD, **b** CAD, **c** T2D, **d** HF, **e** Dementia, and **f** GERD. The horizontal line corresponds to exome-wide significance level.

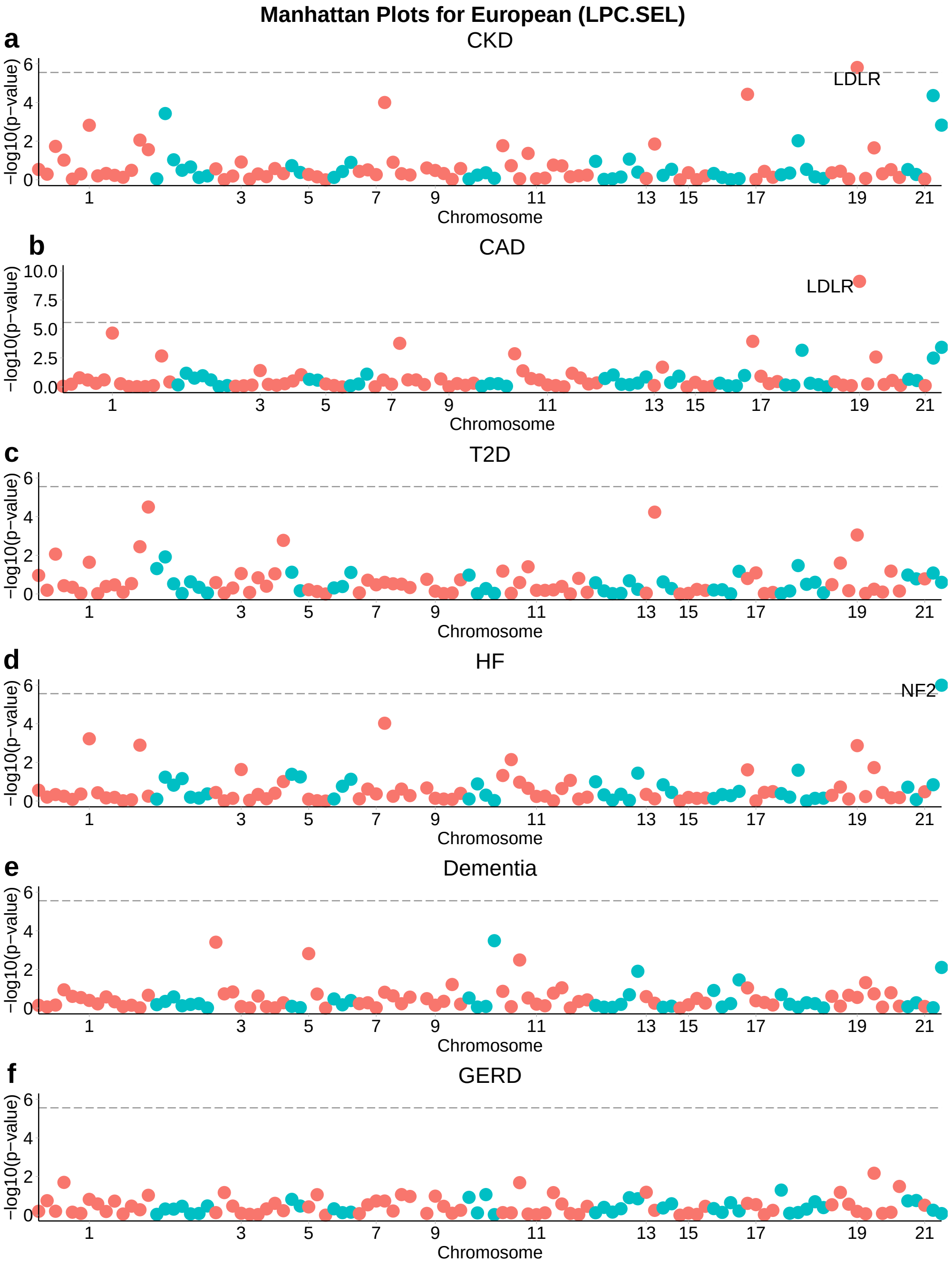

Supplementary Figure 28: **Gene-based test results for the European dataset for 107 autosomal genes on the eMERGE-seq panel using LPC with pre-selected phecodes for six diseases.** Manhattan plots for **a** CKD, **b** CAD, **c** T2D, **d** HF, **e** Dementia, and **f** GERD. The horizontal line corresponds to exome-wide significance level.

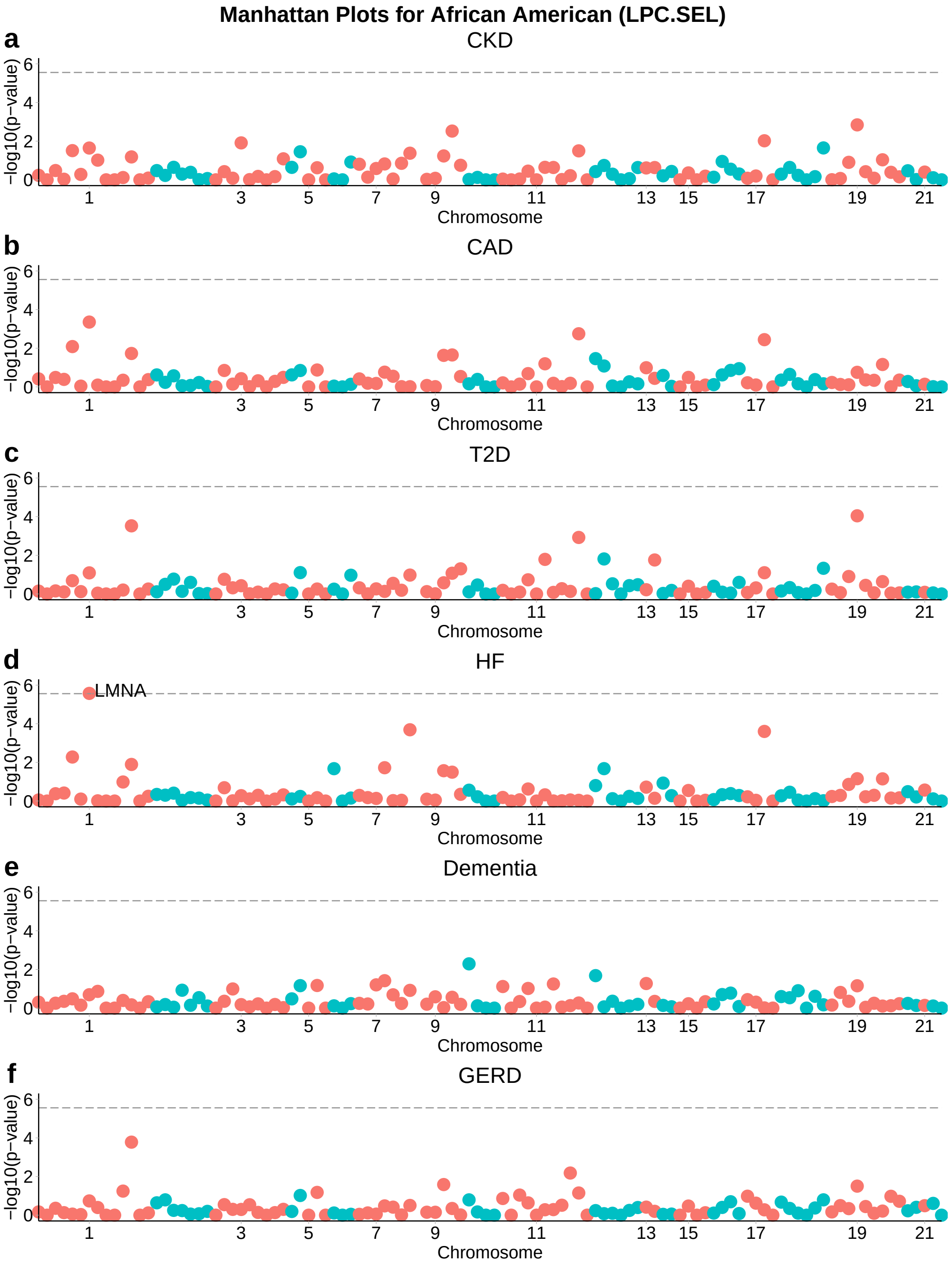

Supplementary Figure 29: **Gene-based test results for the African American dataset for 107 autosomal genes on the eMERGE-seq panel using LPC with pre-selected phecodes for six diseases.** Manhattan plots for **a** CKD, **b** CAD, **c** T2D, **d** HF, **e** Dementia, and **f** GERD. The horizontal line corresponds to exome-wide significance level.

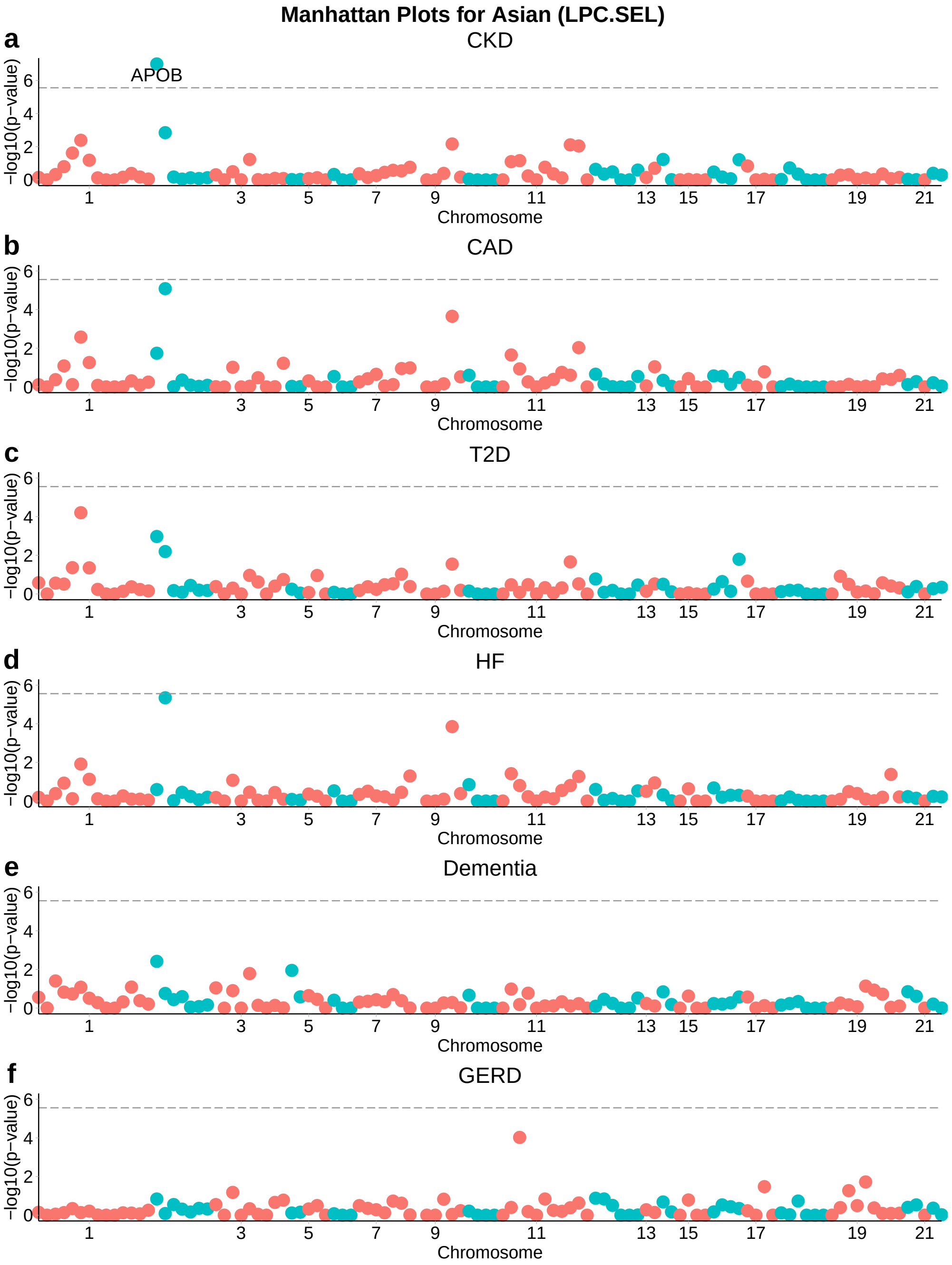

Supplementary Figure 30: **Gene-based test results for the Asian dataset for 107 autosomal genes on the eMERGE-seq panel using LPC with pre-selected phecodes for six diseases.** Manhattan plots for **a** CKD, **b** CAD, **c** T2D, **d** HF, **e** Dementia, and **f** GERD. The horizontal line corresponds to exome-wide significance level.

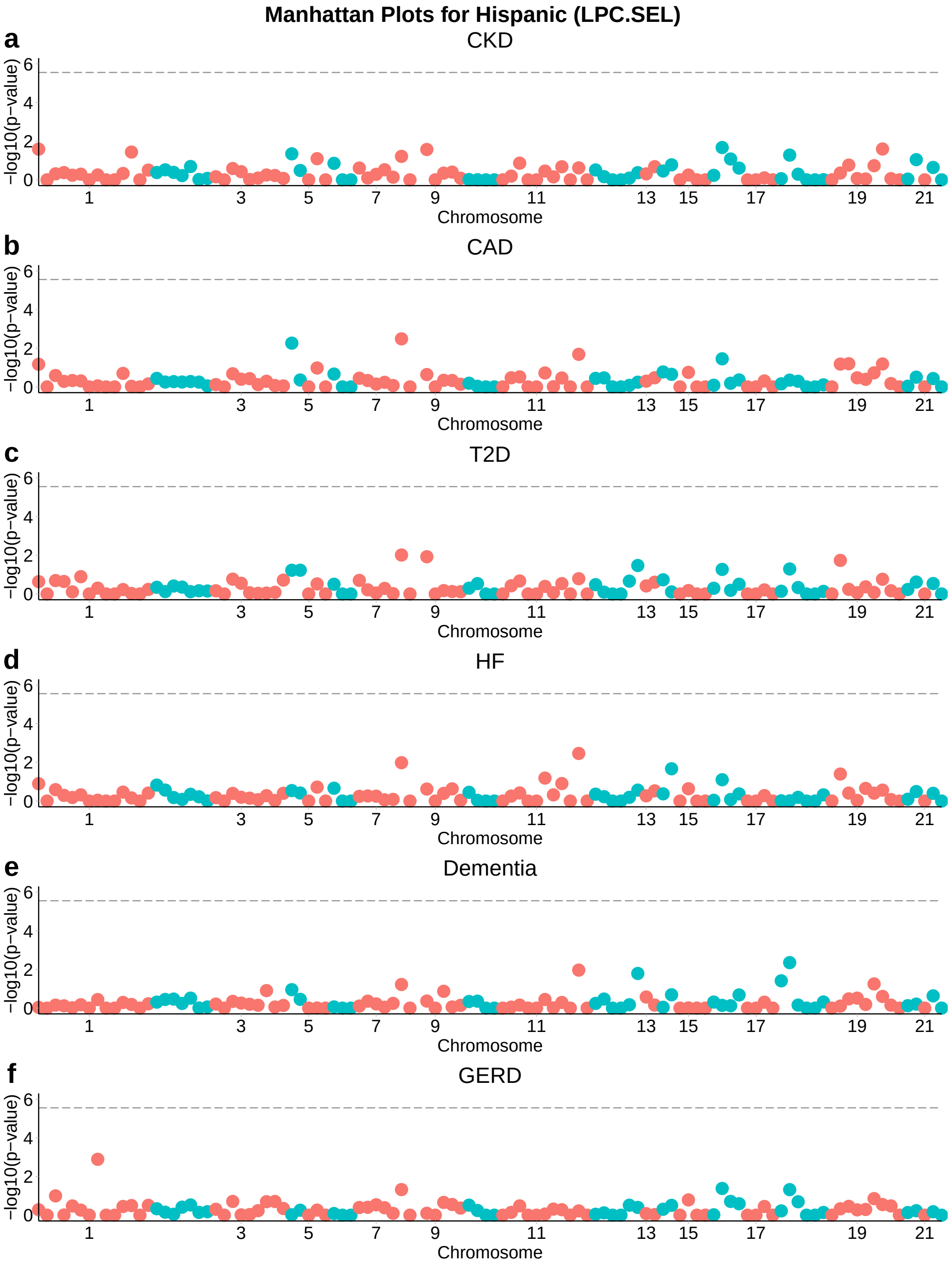

Supplementary Figure 31: **Gene-based test results for the Hispanic dataset for 107 autosomal genes on the eMERGE-seq panel using LPC with pre-selected phecodes for six diseases.** Manhattan plots for **a** CKD, **b** CAD, **c** T2D, **d** HF, **e** Dementia, and **f** GERD. The horizontal line corresponds to exome-wide significance level.

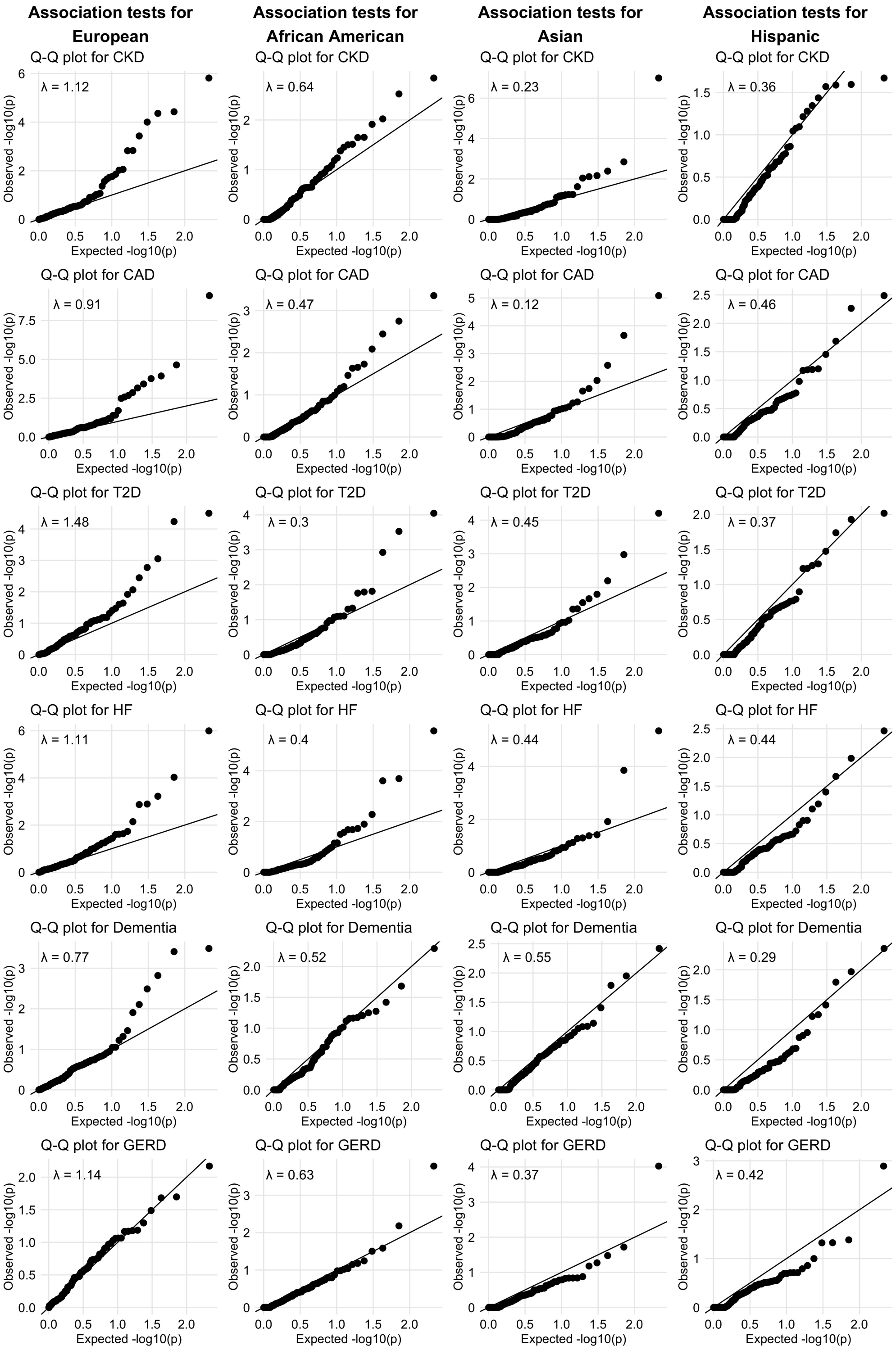

Supplementary Figure 32: **QQ plots for gene-based tests for 107 autosomal genes on the eMERGE-seq panel using LPC with pre-selected phecodes for six diseases.** Results for four ethnic groups are shown.

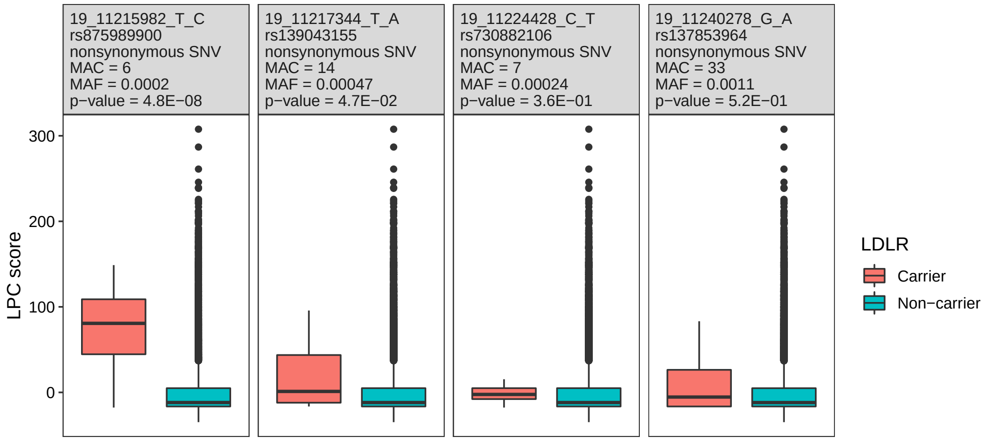

Supplementary Figure 33: **Boxplot of CKD LPC scores for carriers vs. non-carriers of rare variants in *LDLR* (Europeans).**

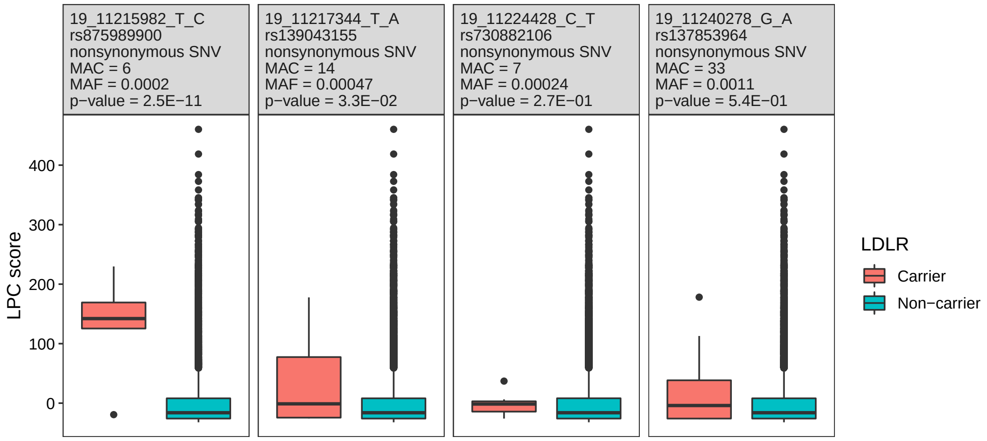

Supplementary Figure 34: **Boxplot of CAD LPC scores for carriers vs. non-carriers of rare variants in *LDLR* (Europeans).**

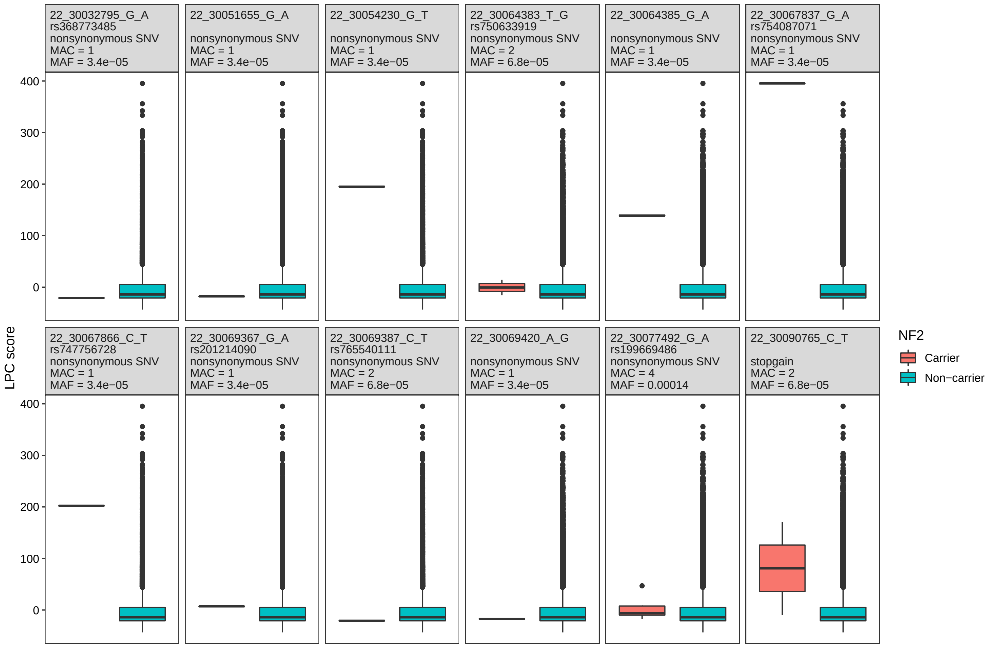

Supplementary Figure 35: **Boxplot of HF LPC scores for carriers vs. non-carriers of ultra-rare variants in *NF2* (Europeans).**

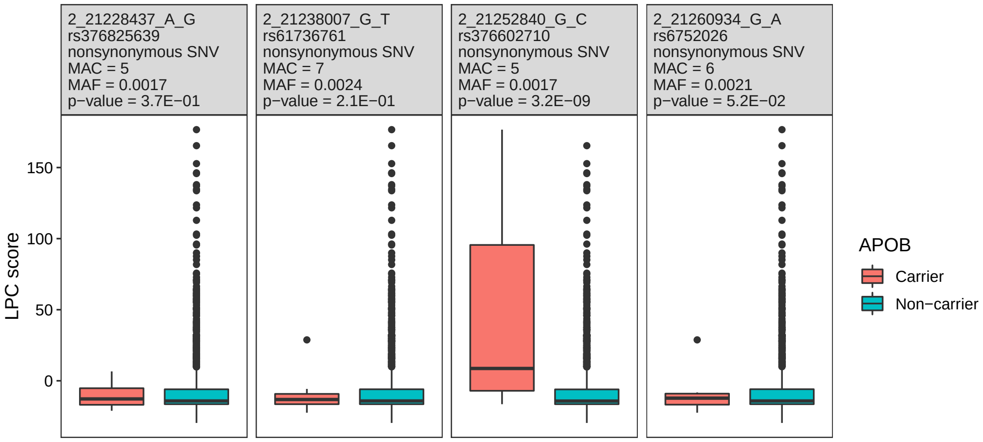

Supplementary Figure 36: **Boxplot of CKD LPC scores for carriers vs. non-carriers of rare variants in *APOB* (Asians).**

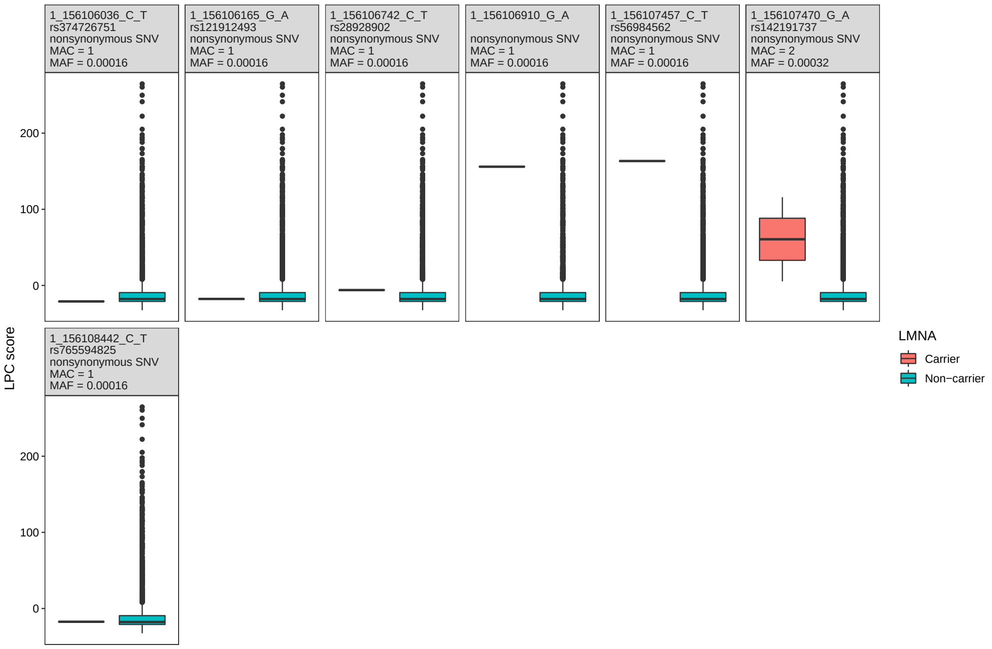

Supplementary Figure 37: **Boxplot of HF LPC scores for carriers vs. non-carriers of ultra-rare variants in *LMNA* (African American).**

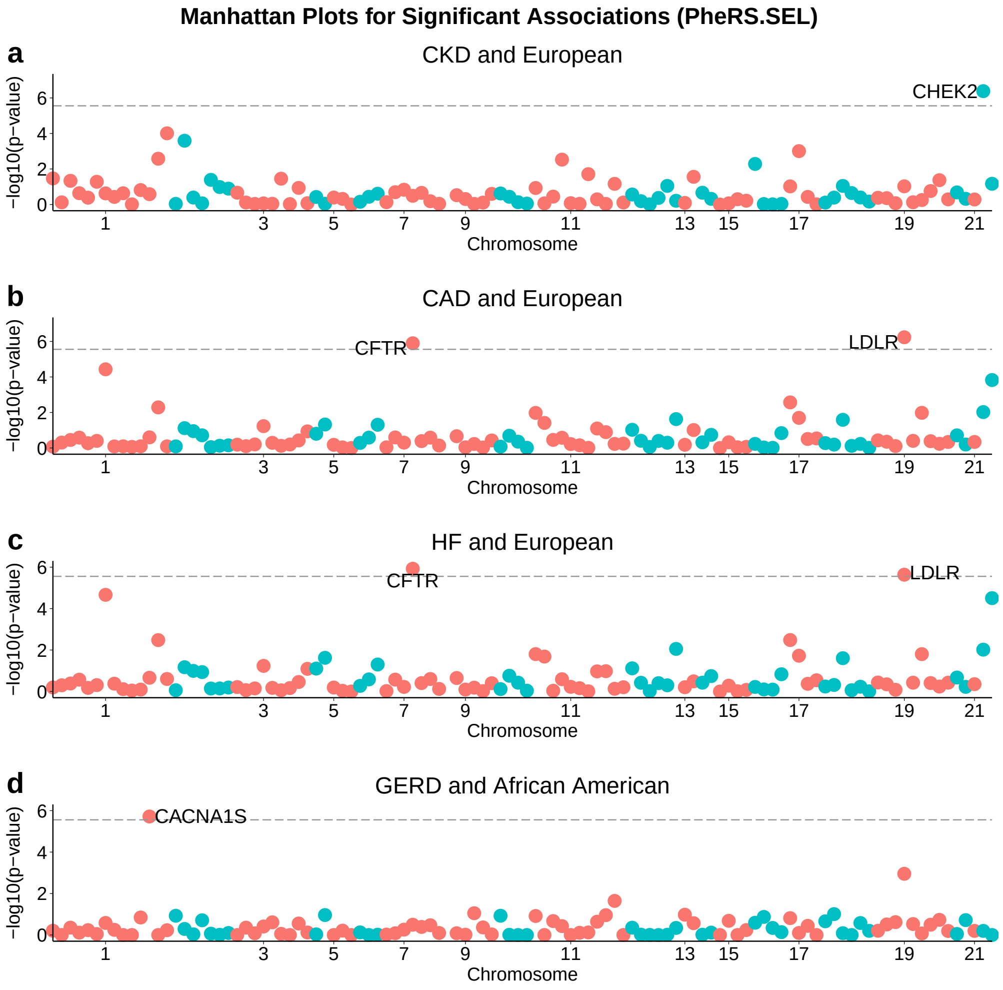

Supplementary Figure 38: **Exome-wide significant gene-based test results for 107 autosomal genes on the eMERGE-seq panel using PheRS with pre-selected phecodes for six diseases.** Results are shown for those phenotypes and ethnic groups with at least one exome-wide significant result. Manhattan plots for **a** CKD, **b** CAD, **c** T2D, **d** HF, **e** Dementia, and **f** GERD. The horizontal line corresponds to exome-wide significance level.

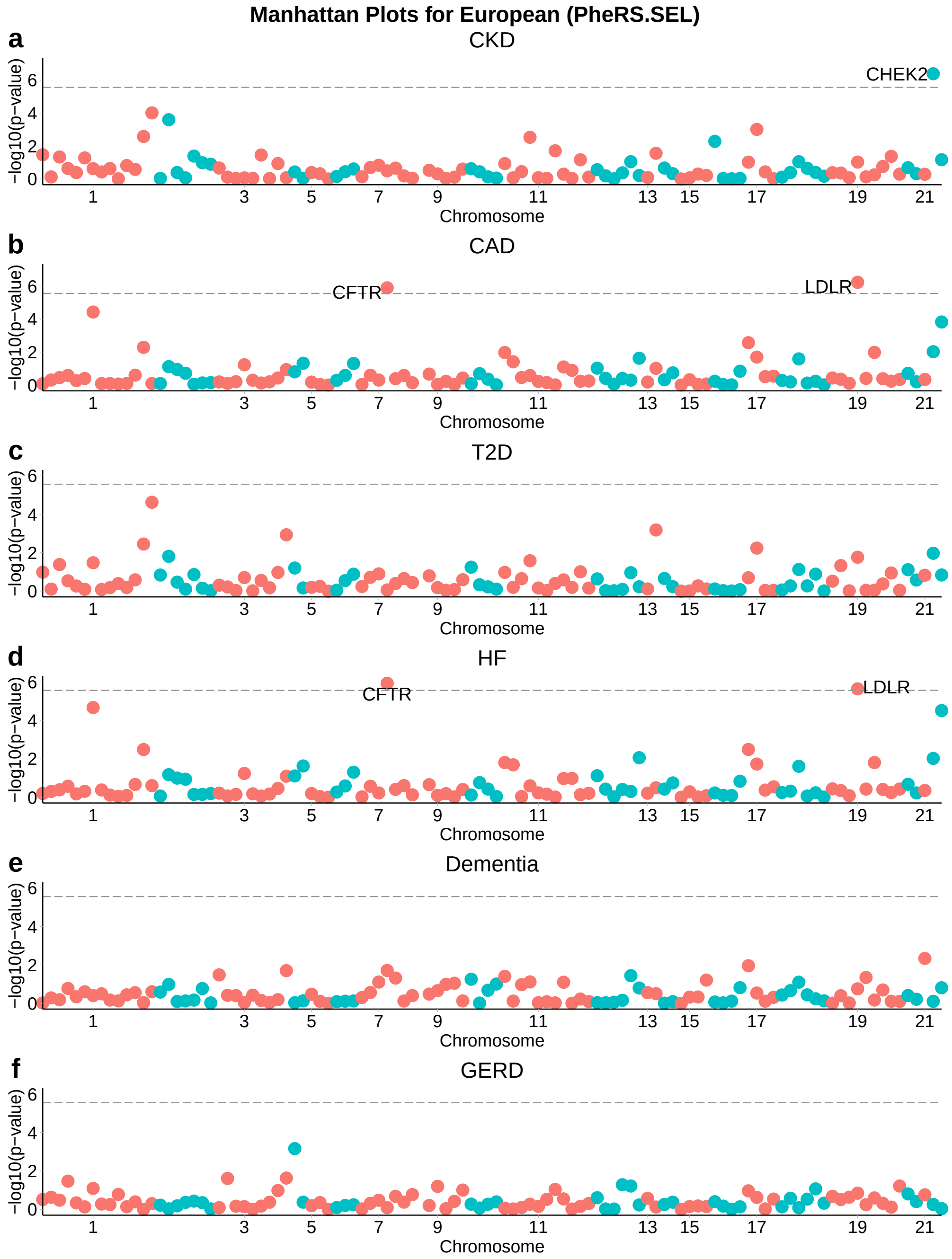

Supplementary Figure 39: **Gene-based test results for the European dataset for 107 autosomal genes on the eMERGE-seq panel using PheRS with pre-selected phecodes for six diseases.** Manhattan plots for **a** CKD, **b** CAD, **c** T2D, **d** HF, **e** Dementia, and **f** GERD. The horizontal line corresponds to exome-wide significance level.

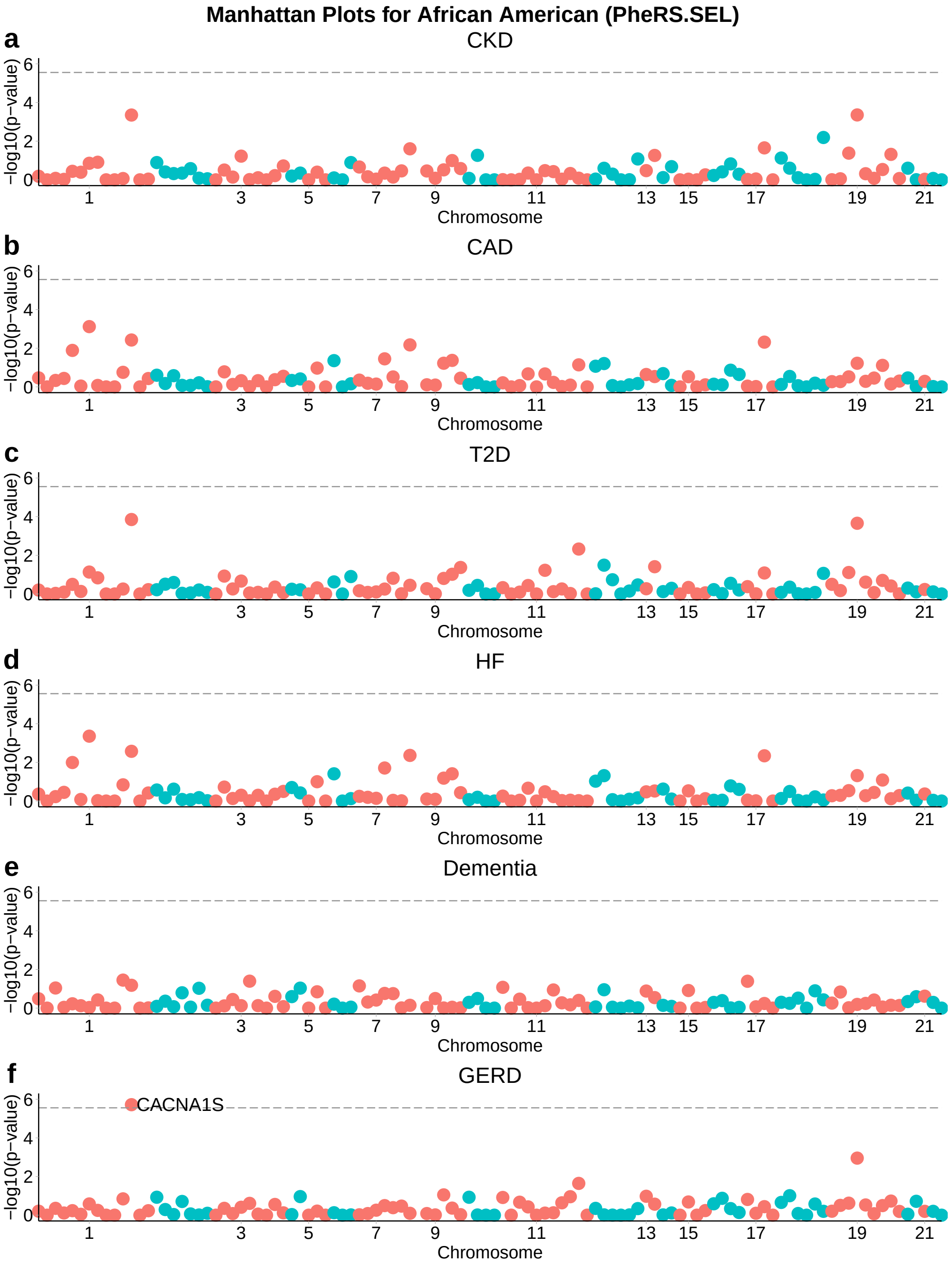

Supplementary Figure 40: **Gene-based test results for the African American dataset for 107 autosomal genes on the eMERGE-seq panel using PheRS with pre-selected phecodes for six diseases.** Manhattan plots for **a** CKD, **b** CAD, **c** T2D, **d** HF, **e** Dementia, and **f** GERD. The horizontal line corresponds to exome-wide significance level.

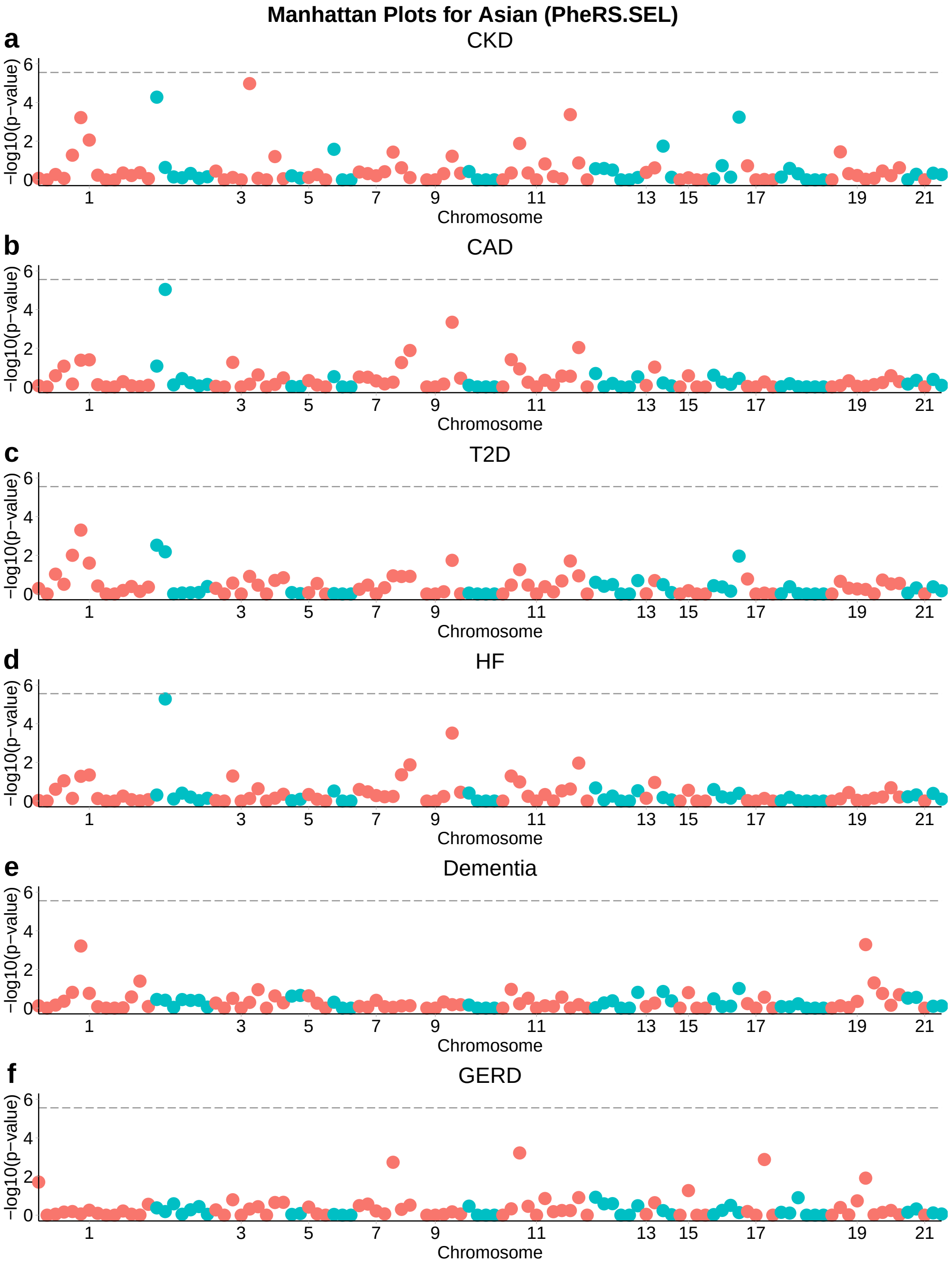

Supplementary Figure 41: **Gene-based test results for the Asian dataset for 107 autosomal genes on the eMERGE-seq panel using PheRS with pre-selected phecodes for six diseases.** Manhattan plots for **a** CKD, **b** CAD, **c** T2D, **d** HF, **e** Dementia, and **f** GERD. The horizontal line corresponds to exome-wide significance level.

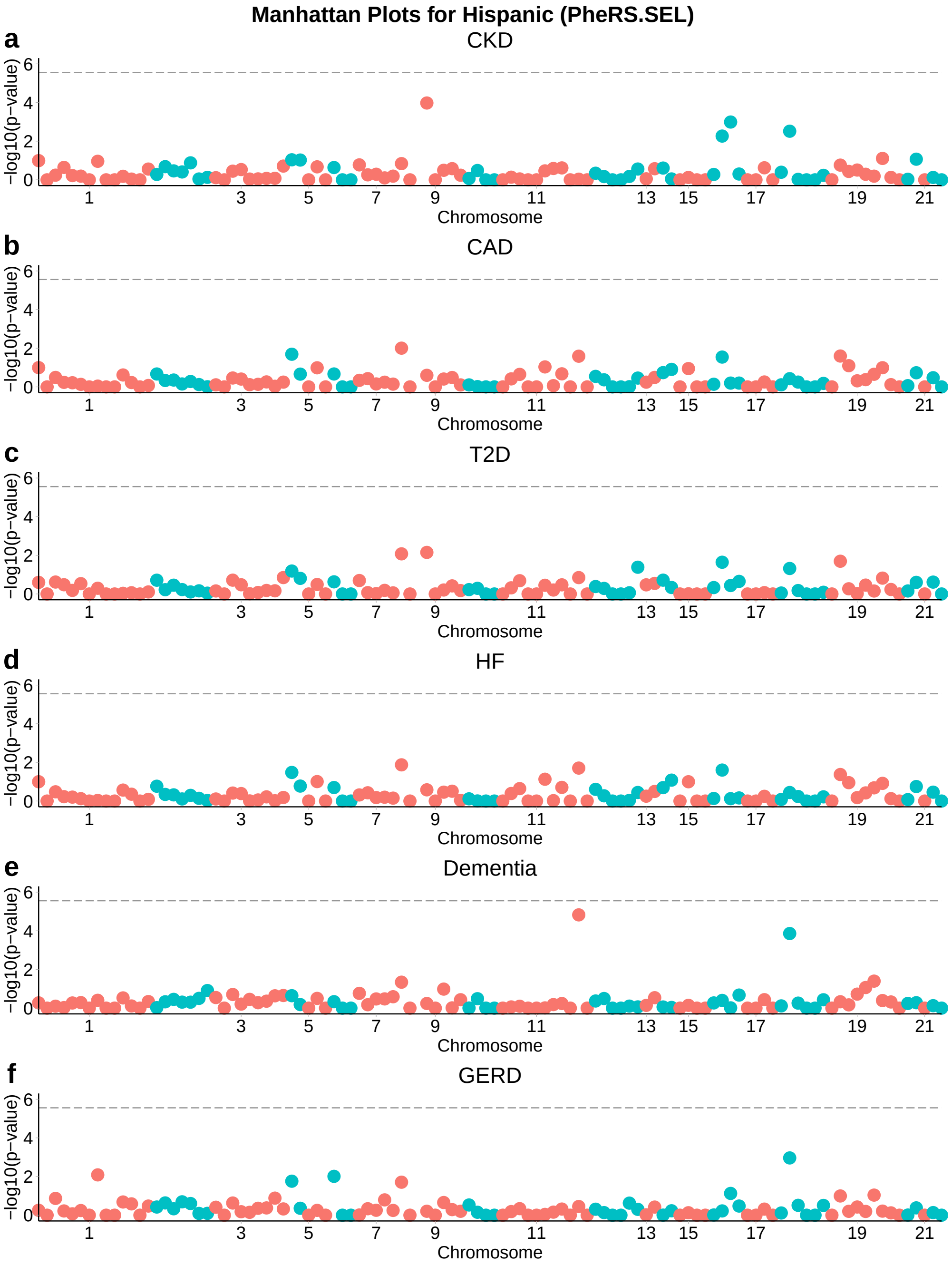

Supplementary Figure 42: **Gene-based test results for the Hispanic dataset for 107 autosomal genes on the eMERGE-seq panel using PheRS with pre-selected phecodes for six diseases.** Manhattan plots for **a** CKD, **b** CAD, **c** T2D, **d** HF, **e** Dementia, and **f** GERD. The horizontal line corresponds to exome-wide significance level.

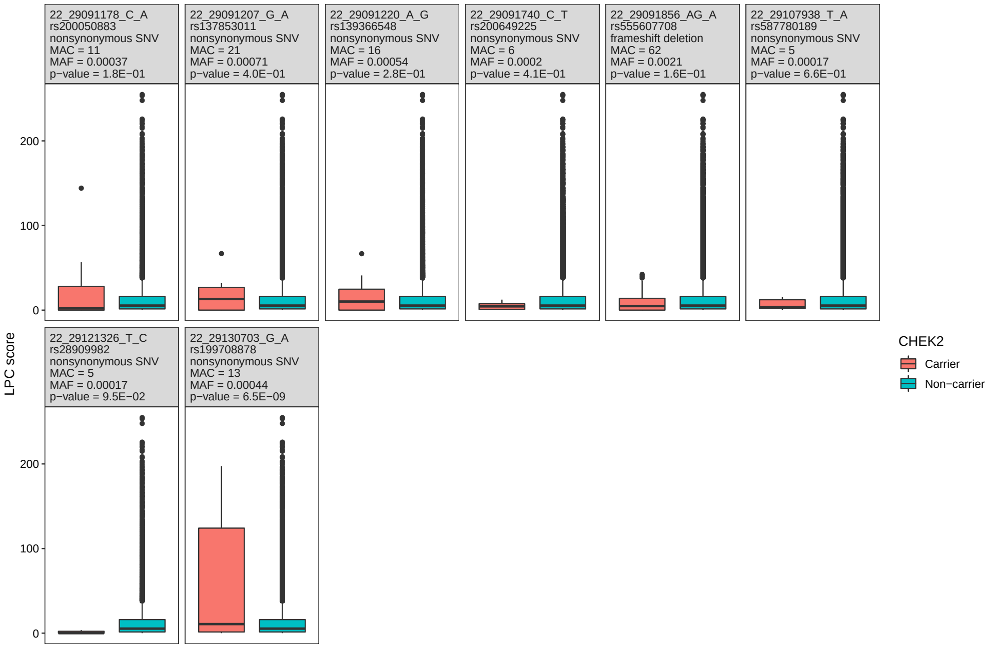

Supplementary Figure 43: **Boxplot of CKD LPC scores for carriers vs. non-carriers of rare variants in *CHEK2* (Europeans).**

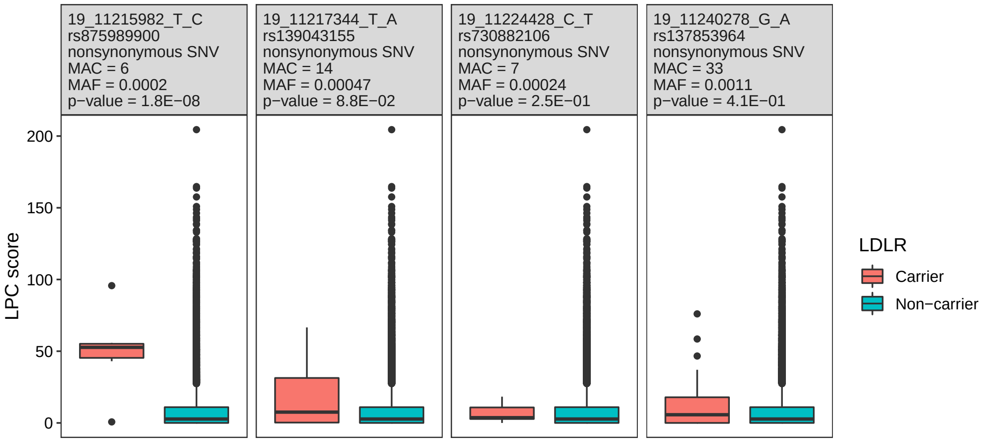

Supplementary Figure 44: **Boxplot of CAD LPC scores for carriers vs. non-carriers of rare variants in *LDLR* (Europeans).**

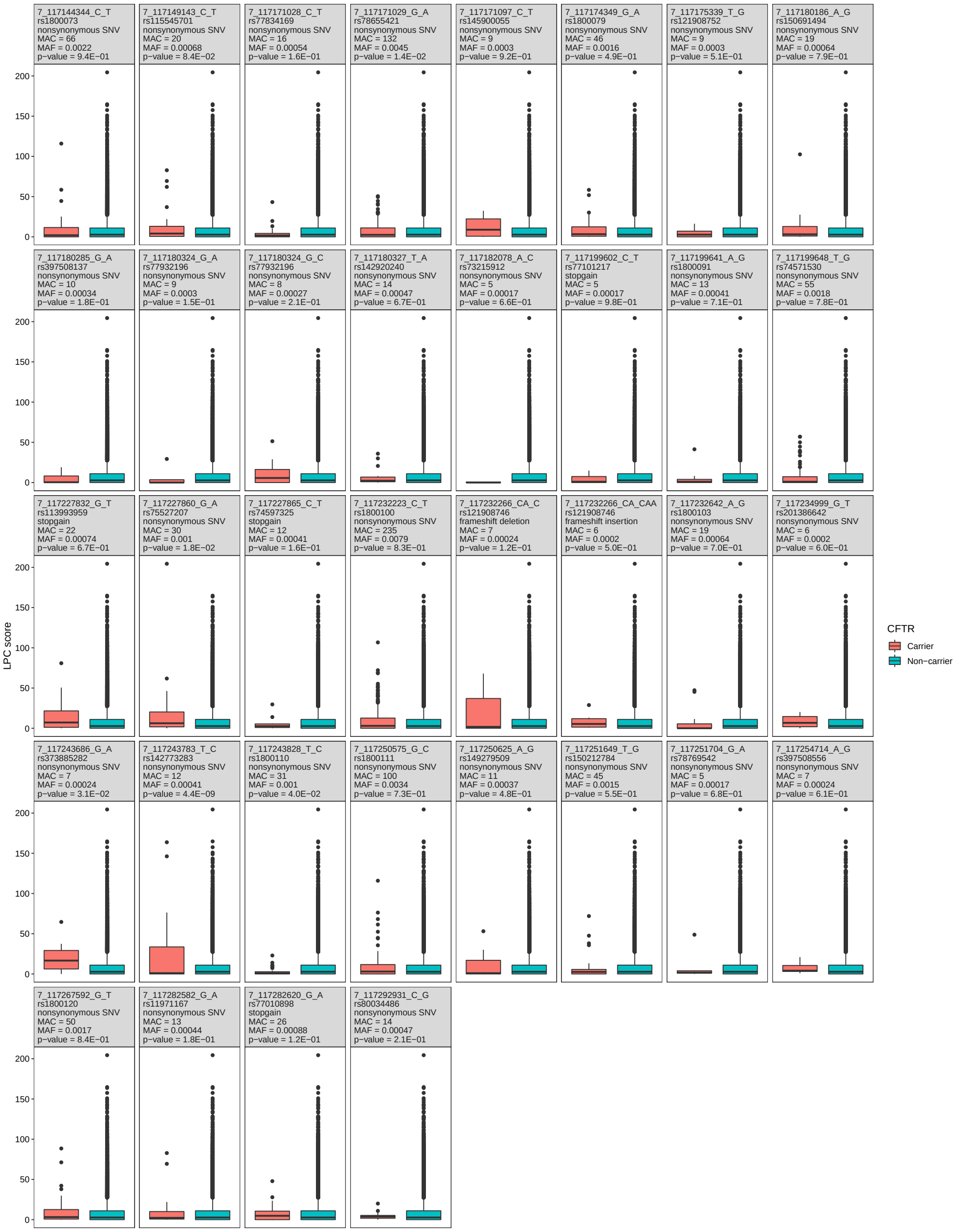

Supplementary Figure 45: **Boxplot of CAD LPC scores for carriers vs. non-carriers of rare variants in *CFTR* (Europeans).**

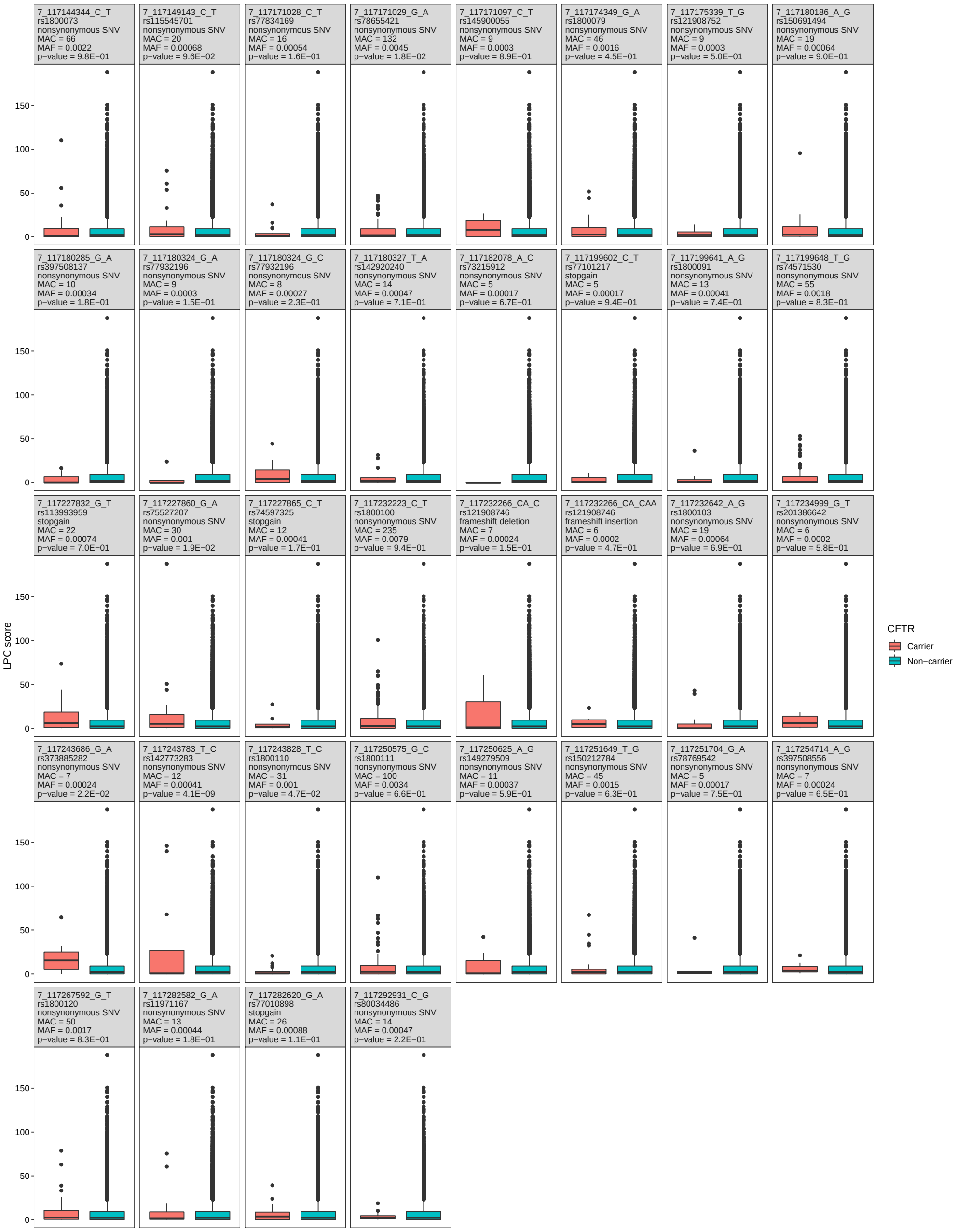

Supplementary Figure 46: **Boxplot of HF LPC scores for carriers vs. non-carriers of rare variants in *CFTR* (Europeans).**

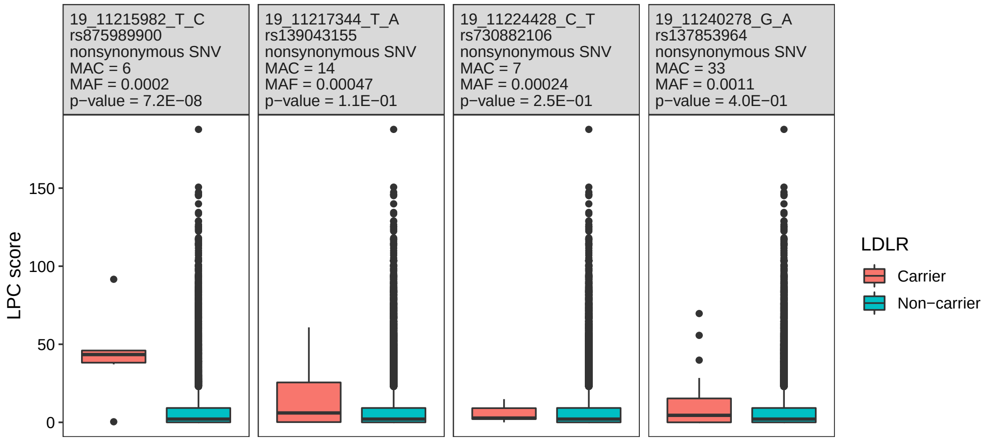

Supplementary Figure 47: **Boxplot of HF LPC scores for carriers vs. non-carriers of rare variants in *LDLR* (Europeans).**

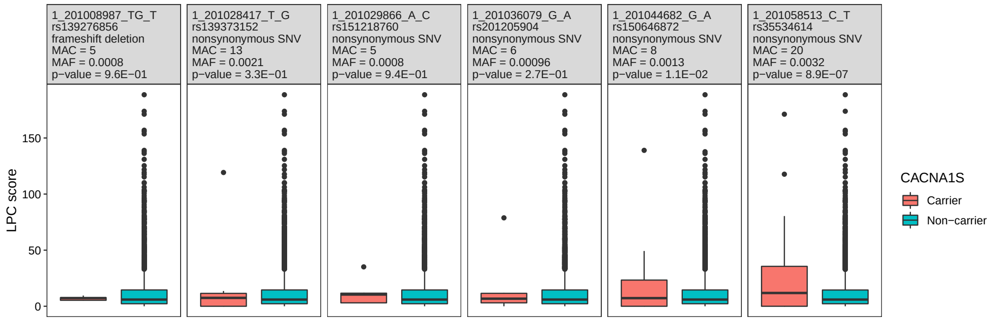

Supplementary Figure 48: **Boxplot of GERD LPC scores for carriers vs. non-carriers of ultra-rare variants in *CACNA1S* (African American).**

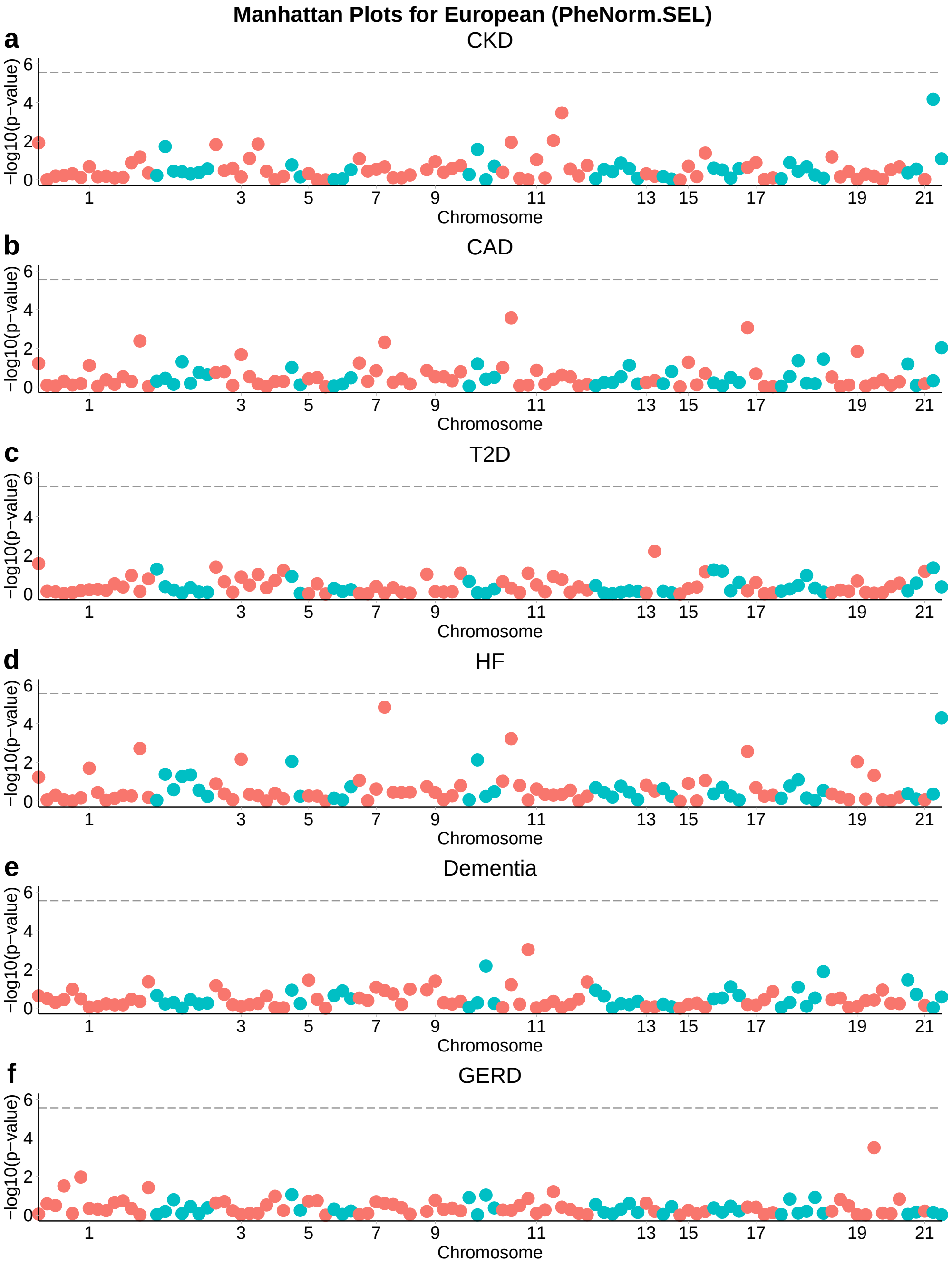

Supplementary Figure 49: **Gene-based test results for the European dataset for 107 autosomal genes on the eMERGE-seq panel using PheNorm with pre-selected phecodes for six diseases.** Manhattan plots for **a** CKD, **b** CAD, **c** T2D, **d** HF, **e** Dementia, and **f** GERD. The horizontal line corresponds to exome-wide significance level.

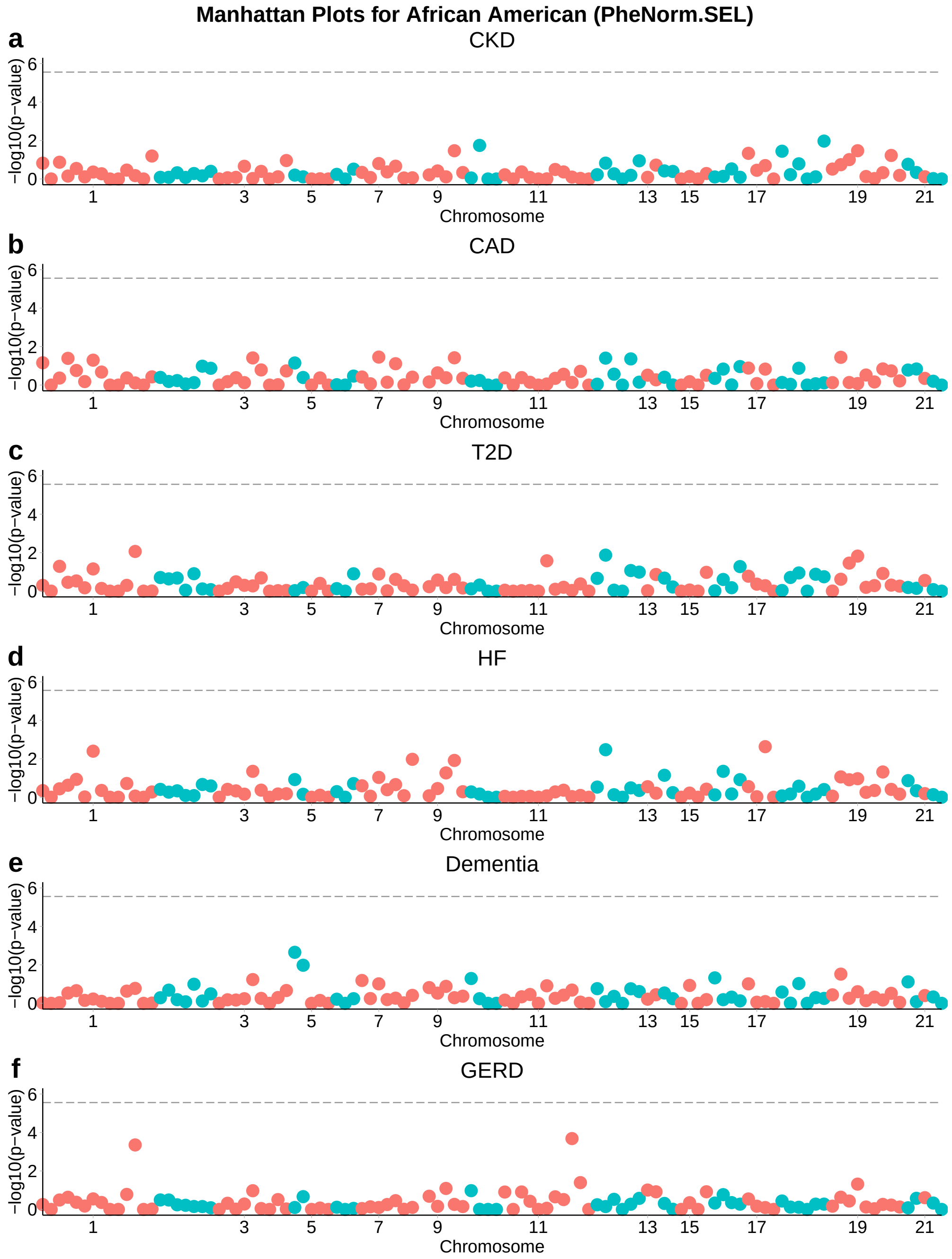

Supplementary Figure 50: **Gene-based test results for the African American for 107 autosomal genes on the eMERGE-seq panel using PheNorm with pre-selected phecodes for six diseases.** Manhattan plots for **a** CKD, **b** CAD, **c** T2D, **d** HF, **e** Dementia, and **f** GERD. The horizontal line corresponds to exome-wide significance level.

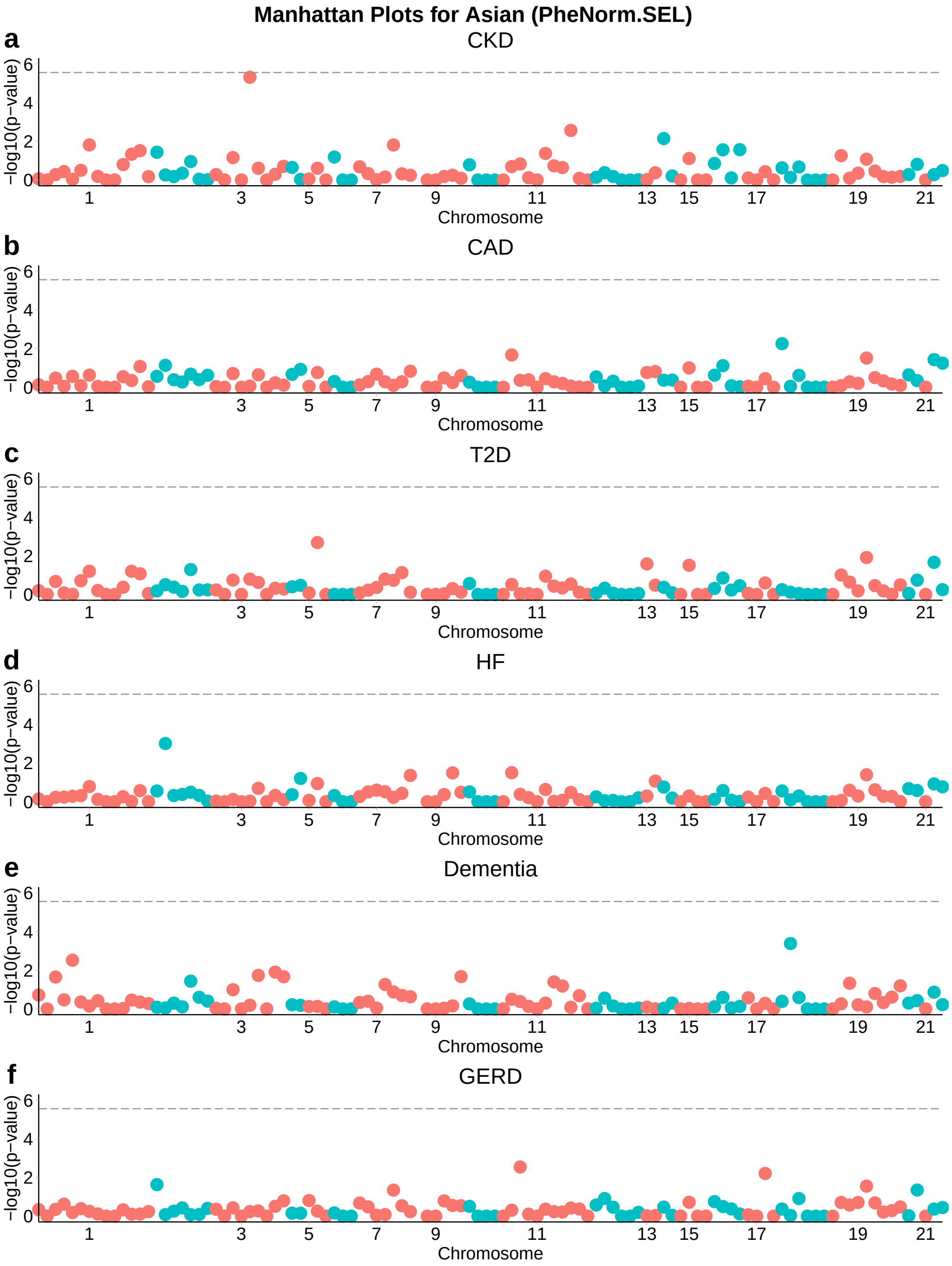

Supplementary Figure 51: **Gene-based test results for the Asian dataset for 107 autosomal genes on the eMERGE-seq panel using PheNorm with pre-selected phecodes for six diseases.** Manhattan plots for **a** CKD, **b** CAD, **c** T2D, **d** HF, **e** Dementia, and **f** GERD. The horizontal line corresponds to exome-wide significance level.

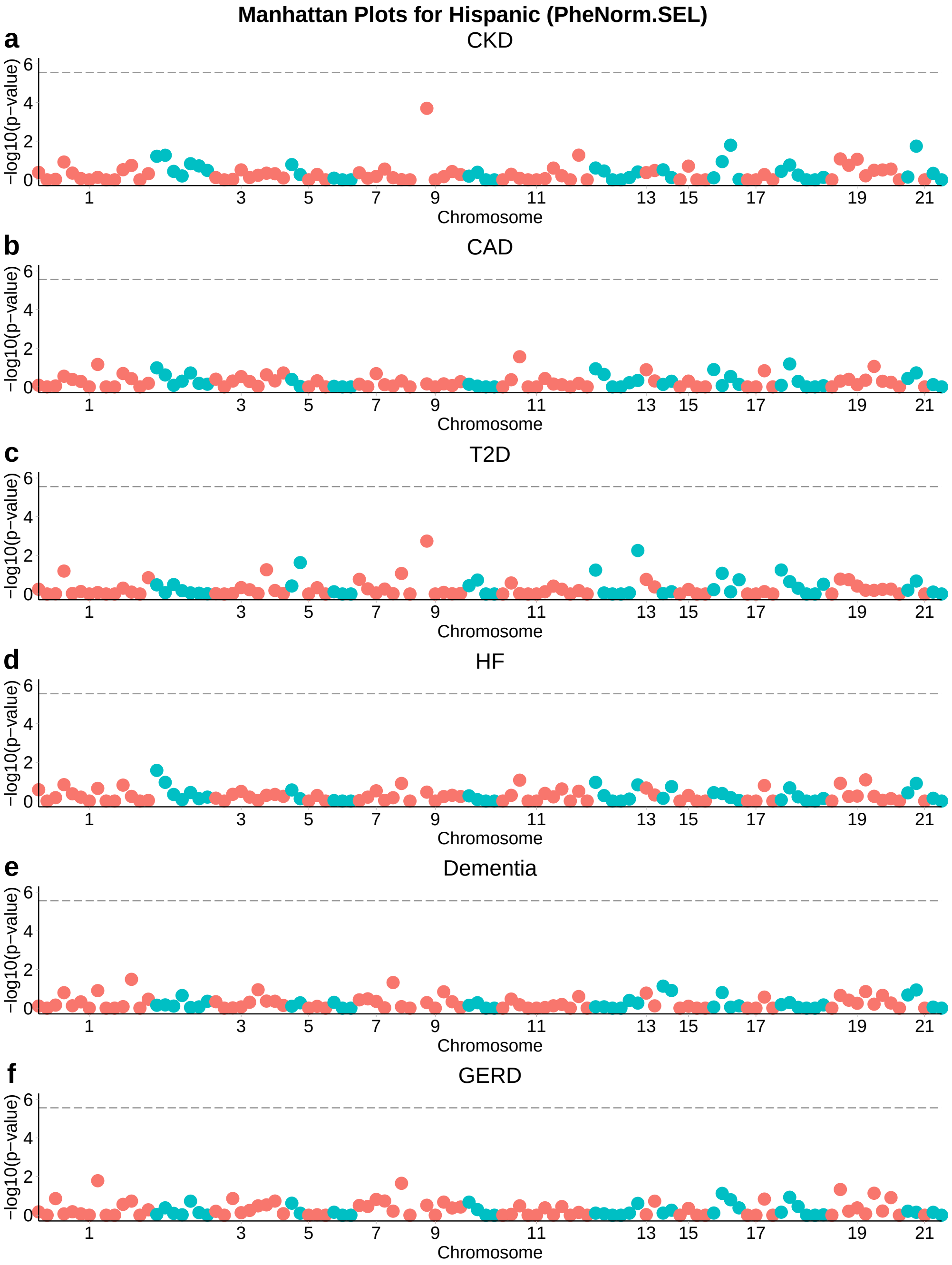

Supplementary Figure 52: **Gene-based test results for the Hispanic dataset for 107 autosomal genes on the eMERGE-seq panel using PheNorm with pre-selected phecodes for six diseases.** Manhattan plots for **a** CKD, **b** CAD, **c** T2D, **d** HF, **e** Dementia, and **f** GERD. The horizontal line corresponds to exome-wide significance level.

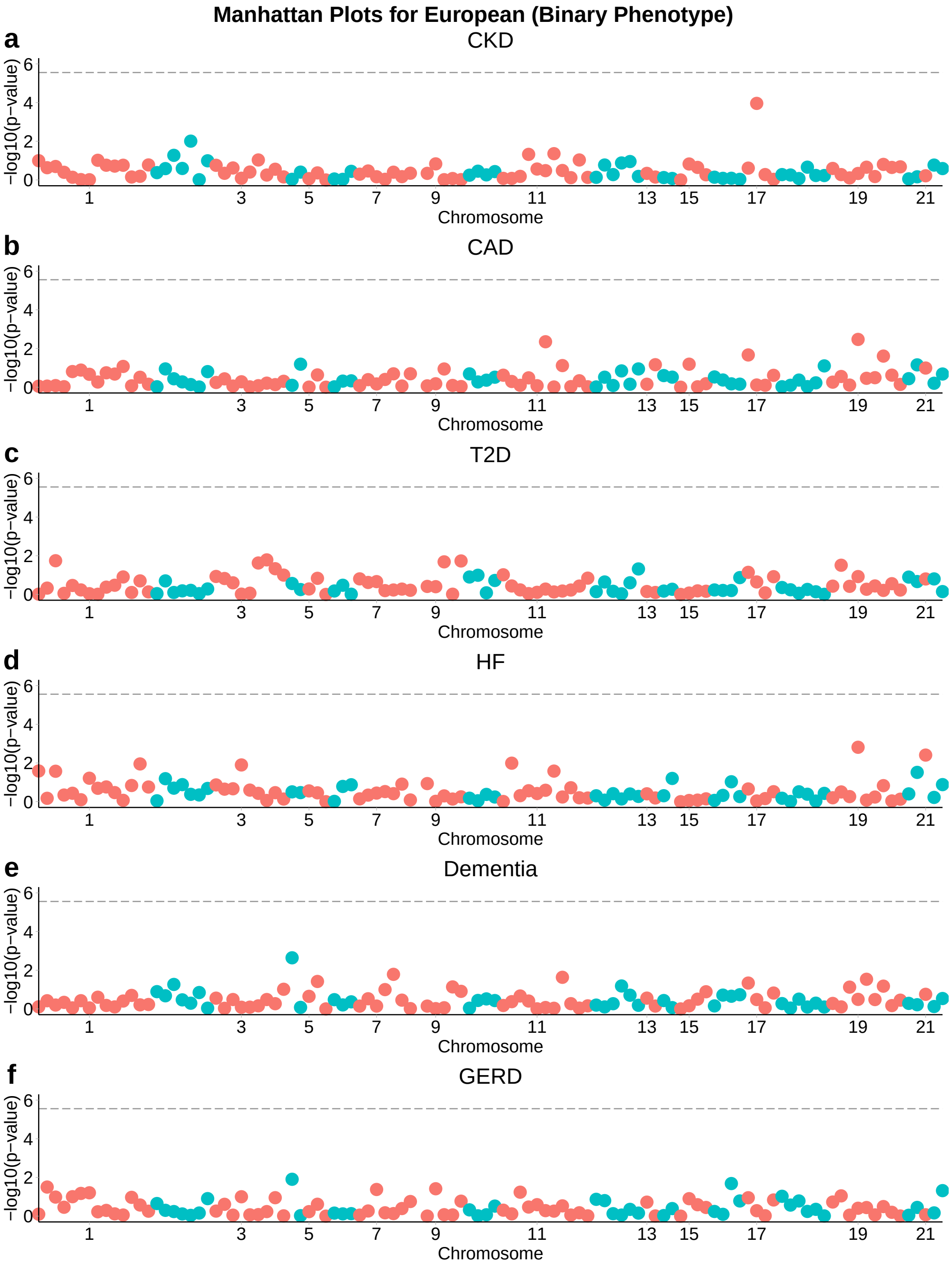

Supplementary Figure 53: **Gene-based test results for the European dataset for 107 autosomal genes on the eMERGE-seq panel using a binary trait defined by phecodes for six diseases.** Manhattan plots for **a** CKD, **b** CAD, **c** T2D, **d** HF, **e** Dementia, and **f** GERD. The horizontal line corresponds to exome-wide significance level.

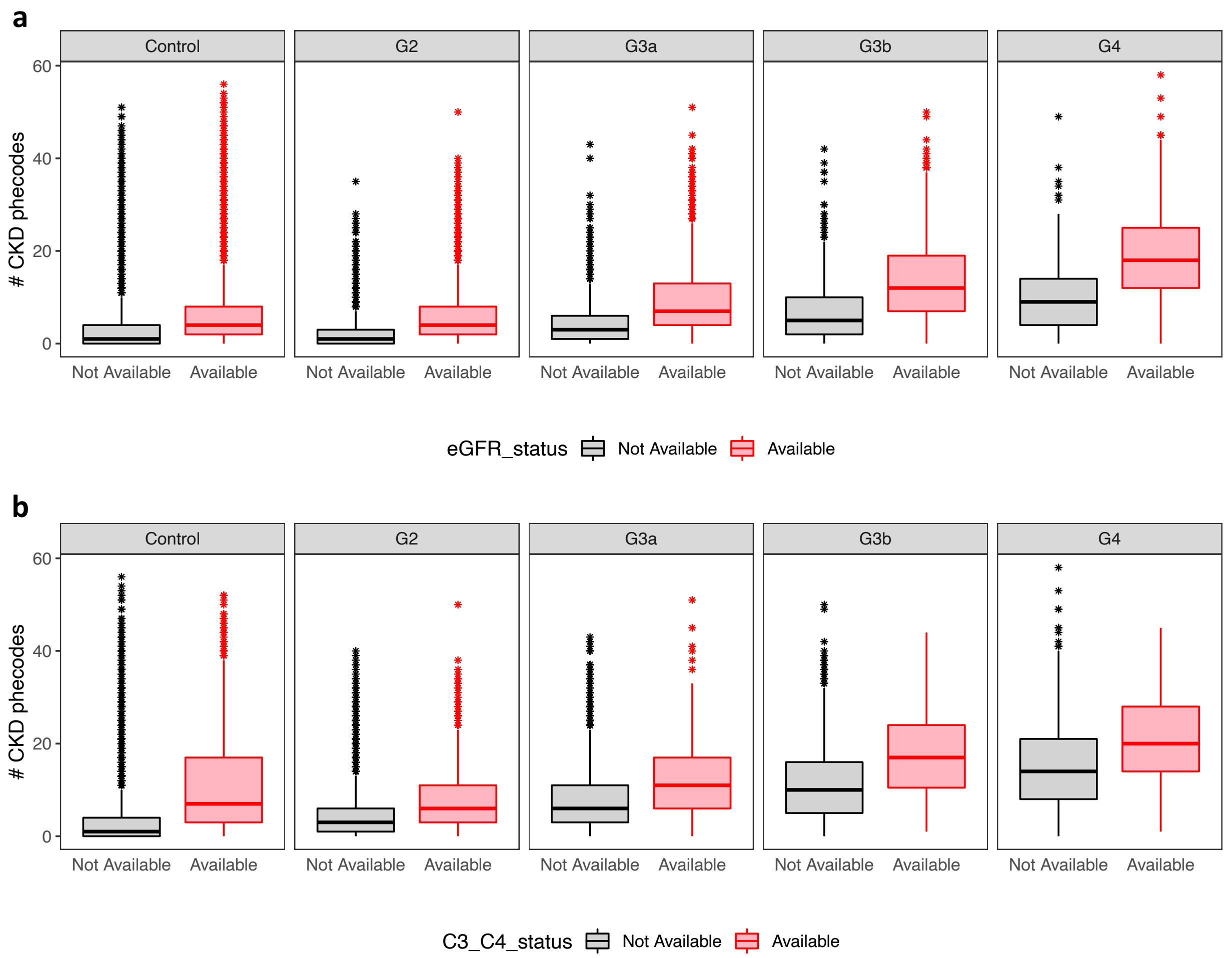

Supplementary Figure 54: **CKD related phecodes and presence of laboratory values. a** Burden of CKD related phecodes in individuals with eGFR slope available vs. not available; bf b Burden of CKD related phecodes in individuals with C3/C4 available vs. not available.

Supplementary Tables

Supplementary Table 1: **The number of phecodes with non-zero prevalence in the eMERGE dataset for each category.**

Category	# phecodes
Genitourinary	175
Circulatory system	172
Endocrine/metabolic	169
Digestive	166
Neoplasms	141
Musculoskeletal	132
Sense organs	128
Injuries & poisonings	126
Dermatologic	96
Respiratory	85
Neurological	84
Mental disorders	76
Hematopoietic	62
Pregnancy complications	58
Congenital anomalies	56
Symptoms	50
Infectious diseases	23
NULL	18

Supplementary Table 2: **Phecodes that are used to define cases for phenotypes (at least one occurrence) in eMERGE dataset.** These phecodes are excluded from the derivation of the LPC scores, when evaluating the predictive performance on test data.

Phenotype	Phecode	Description
CKD	585.3	Chronic renal failure [CKD]
	585.31	Renal dialysis
	585.32	End stage renal disease
	585.33	Chronic Kidney Disease, Stage III
	585.34	Chronic Kidney Disease, Stage IV
	585.4	Chronic kidney disease, Stage I or II
CAD	411.2	Myocardial infarction
	414	Other forms of chronic heart disease
T2D	250	Diabetes mellitus
	250.2	Type 2 diabetes
	250.21	Type 2 diabetes with ketoacidosis
	250.22	Type 2 diabetes with renal manifestations
	250.23	Type 2 diabetes with ophthalmic manifestations
	250.24	Type 2 diabetes with neurological manifestations
	250.25	Diabetes type 2 with peripheral circulatory disorders
HF	428	Congestive heart failure; nonhypertensive
	428.1	Congestive heart failure (CHF) NOS
	428.2	Heart failure NOS
	428.3	Heart failure with reduced EF [Systolic or combined heart failure]
	428.4	Heart failure with preserved EF [Diastolic heart failure]
Dementia	290	Delirium dementia and amnestic and other cognitive disorders
	290.1	Dementias
	290.11	Alzheimer's disease
	290.12	Dementia with cerebral degenerations
	290.13	Senile dementia
	290.16	Vascular dementia
	290.2	Delirium due to conditions classified elsewhere
	317.1	Alcoholism
	292.2	Mild cognitive impairment
	317	Alcohol-related disorders
	290.3	Other persistent mental disorders due to conditions classified elsewhere
GERD	530.1	Esophagitis, GERD and related diseases
	530.11	GERD
	530.14	Reflux esophagitis

Supplementary Table 3: **The choice of 'relevant' phecodes.** AUROC and AUPRC for different sets of T2D feature phecodes that include (1) 152 phecodes with p-value $< 10^{-5}$ in logistic regression on PRS, (2) 140 phecodes that are manually selected by experts, (3) 132 phecodes are in the intersection of the previous two sets. The case defining phecodes are excluded.

	AUROC			AUPRC		
# phecodes	152	140	132	152	140	132
PheRS.SEL	0.7377	0.7473	0.7492	0.5397	0.5533	0.5563
LPC.SEL	0.7765	0.7658	0.7644	0.5974	0.5863	0.5904
PheNorm.SEL	0.7470	0.7592	0.7599	0.6512	0.6653	0.6650

Supplementary Table 4: **P-values for the replication of discovered variants in different populations.** The nominally significant p-values from replication cohorts are highlighted in bold.

Ethnicity	Phenotype	Gene	European	African American	Asian	Hispanic
LPC with pre-selected phecodes						
European	CKD	LDLR	1.530E-06	**1.431E-03**	9.341E-01	8.565E-01
European	CAD	LDLR	7.852E-10	1.760E-01	9.822E-01	3.386E-01
European	HF	NF2	1.009E-06	NA	6.118E-01	NA
African American	HF	LMNA	**5.987E-04**	2.680E-06	7.488E-02	NA
Asian	CKD	APOB	8.904E-01	3.329E-01	1.006E-07	4.180E-01
PheRS with pre-selected phecodes						
Ethnicity	Phenotype	Gene	p.European	p.African	p.Asian	p.Hispanic
European	CKD	CHEK2	4.13E-07	8.49E-01	4.53E-01	7.52E-01
European	CAD	CFTR	1.27E-06	**3.53E-02**	7.03E-01	5.71E-01
European	CAD	LDLR	5.82E-07	5.98E-02	9.31E-01	4.85E-01
European	HF	CFTR	1.19E-06	**1.95E-02**	6.00E-01	6.34E-01
European	HF	LDLR	2.31E-06	**4.81E-02**	9.10E-01	6.65E-01
African American	GERD	CACNA1S	4.07E-01	1.91E-06	8.89E-01	2.60E-01

Supplementary Table 5: **Number of individuals (cases vs. controls) for four ethnic groups in the eMERGE-seq dataset.** Cases/Controls are defined based on presence/absence of specific phecodes as explained in the text.

Population	Phenotype	Total	Case	Control	Control/Case Imbalance
European	CKD	14,813	1,806	13,007	7.2
	CAD	14,813	1,565	13,248	8.5
	T2D	14,813	2,567	12,246	4.8
	HF	14,813	1,328	13,485	10.2
	Dementia	14,813	1,442	13,371	9.3
	GERD	14,813	5,487	9,326	1.7
African American	CKD	3,110	356	2,754	7.7
	CAD	3,110	164	2,946	18
	T2D	3,110	427	2,683	6.3
	HF	3,110	189	2,921	15.5
	Dementia	3,110	194	2,916	15
	GERD	3,110	850	2,260	2.7
Asian	CKD	1,437	145	1,292	8.9
	CAD	1,437	65	1,372	21.1
	T2D	1,437	210	1,227	5.8
	HF	1,437	47	1,390	29.6
	Dementia	1,437	41	1,396	34
	GERD	1,437	371	1,066	2.9
Hispanic	CKD	1,301	284	1,017	3.6
	CAD	1,301	128	1,173	9.2
	T2D	1,301	340	961	2.8
	HF	1,301	143	1,158	8.1
	Dementia	1,301	122	1,179	9.7
	GERD	1,301	468	833	1.8

Supplementary Table 6: **Genes showing significant association with at least one disease using LPC and PheRS with pre-selected phecodes.** The p values from the combined test (p.all), the tests including only common variants (p.common), and tests including only rare variants (p.rare), as well as individual tests in the overall combined test are also reported, where the smallest individual test p value for each gene is in bold. No exome-wide significant results were identified for PheNorm.

Ethnicity	Disease	Gene	p.all	p.common	p.rare	p.burden					p.dispersion					p.singleCauchy	
						MAC< 5	MAF< 0.01&MAC≥ 5 &Beta	MAF< 0.01&MAC≥ 5 &CADD	MAF< 0.01&MAC≥ 5 &Polyphen	MAF≥ 0.01 &Beta	MAC< 5	MAF< 0.01&MAC≥ 5 &Beta	MAF< 0.01&MAC≥ 5 &CADD	MAF< 0.01&MAC≥ 5 &Polyphen	MAF≥ 0.01 &Beta	MAF< 0.01&MAC≥ 5	MAF≥ 0.01
LPC with pre-selected phecodes																	
European	CKD	LDLR	1.530E-06	NA	1.530E-06	1.108E-02	5.219E-02	NA	8.189E-02	5.604E-02	NA	2.097E-02	NA	2.551E-02	2.228E-02	**1.912E-07**	NA
European	CAD	LDLR	7.852E-10	NA	7.852E-10	1.620E-04	1.856E-02	NA	3.238E-02	2.042E-02	NA	1.440E-03	NA	3.078E-03	1.783E-03	**9.815E-11**	NA
European	HF	NF2	1.009E-06	NA	1.009E-06	**1.009E-06**	NA	NA	NA	NA	NA	NA	NA	NA	NA	NA	NA
African American	HF	LMNA	2.680E-06	NA	2.680E-06	**3.351E-07**	2.582E-01	NA	2.361E-01	2.576E-01	NA	1.377E-01	NA	9.463E-02	1.364E-01	4.369E-01	NA
Asian	CKD	APOB	1.006E-07	6.146E-01	7.317E-08	3.807E-01	3.581E-01	6.071E-01	2.646E-01	3.721E-01	NA	3.576E-07	6.291E-01	3.877E-08	4.843E-07	**1.271E-08**	6.071E-01
PheRS with pre-selected phecodes																	
European	CKD	CHEK2	4.128E-07	NA	4.128E-07	2.352E-01	9.822E-01	NA	7.871E-01	9.446E-01	NA	2.288E-03	NA	2.044E-02	1.122E-02	**5.160E-08**	NA
European	CAD	LDLR	5.816E-07	NA	5.816E-07	2.605E-03	1.003E-01	NA	1.458E-01	1.068E-01	NA	2.143E-02	NA	2.430E-02	2.265E-02	**7.271E-08**	NA
European	CAD	CFTR	1.266E-06	NA	1.266E-06	8.737E-01	7.333E-01	NA	5.974E-01	7.019E-01	NA	3.906E-02	NA	8.787E-02	6.020E-02	**1.582E-07**	NA
European	HF	CFTR	1.189E-06	NA	1.189E-06	8.866E-01	7.233E-01	NA	5.785E-01	6.883E-01	NA	5.001E-02	NA	1.056E-01	7.486E-02	**1.487E-07**	NA
European	HF	LDLR	2.306E-06	NA	2.306E-06	2.360E-03	1.379E-01	NA	1.925E-01	1.459E-01	NA	2.577E-02	NA	2.800E-02	2.696E-02	**2.883E-07**	NA
African American	GERD	CACNA1S	1.907E-06	2.455E-01	1.387E-06	5.239E-02	4.571E-06	3.367E-01	9.937E-06	3.334E-06	NA	6.032E-07	2.849E-01	6.623E-07	**5.580E-07**	5.321E-06	1.676E-01
